# Factors Affecting the Radiosensitivity of Hexaploid Wheat to γ-Irradiation: Radiosensitivity of Hexaploid Wheat (*Triticum aestivum* L.)

**DOI:** 10.1371/journal.pone.0161700

**Published:** 2016-08-23

**Authors:** Bing Han, Jiayu Gu, Linshu Zhao, Huijun Guo, Yongdun Xie, Shirong Zhao, Xiyun Song, Longzhi Han, Luxiang Liu

**Affiliations:** 1 Academy of Life Science, Qingdao Agricultural University, Qingdao, China; 2 Institute of Crop Sciences, Chinese Academy of Agricultural Sciences /National Key Facility for Crop Gene Resources and Genetic Improvement /National Center of Space Mutagenesis for Crop Improvement, Beijing, China; Tulane University Health Sciences Center, UNITED STATES

## Abstract

Understanding the radiosensitivity of plants, an important factor in crop mutation breeding programs, requires a thorough investigation of the factors that contribute to this trait. In this study, we used the highly radiosensitive wheat (*Triticum aestivum* L.) variety HY1 and J411, a γ-irradiation-insensitive control, which were screened from a natural population, to examine the factors affecting radiosensitivity, including free radical content and total antioxidant capacity, as well as the expression of *TaKu70* and *TaKu80* (DNA repair-related genes) as measured by real-time PCR. We also investigated the alternative splicing of this gene in the wild-type wheat ecotype by sequence analysis. Free radical contents and total antioxidant capacity significantly increased upon exposure of HY1 wheat to γ-irradiation in a dose-dependent manner. By contrast, in J411, the free radical contents exhibited a similar trend, but the total antioxidant capacity exhibited a downward trend upon increasing γ-irradiation. Additionally, we detected dose-dependent increases in *TaKu70* and *TaKu80* expression levels in γ-irradiated HY1, while in J411, *TaKu70* expression levels increased, followed by a decline. We also detected alternative splicing of *TaKu70* mRNA, namely, intron retention, in HY1 but not in J411. Our findings indicate that γ-irradiation induces oxidative stress and DNA damage in hexaploid wheat, resulting in growth retardation of seedlings, and they suggest that *TaKu70* may play a causal role in radiosensitivity in HY1. Further studies are required to exploit these factors to improve radiosensitivity in other wheat varieties.

## Introduction

The induced mutation technique, an important application of nuclear technology in agriculture, has significantly contributed to crop germplasm enhancement and new mutant variety development. Studies investigating radiation sensitivity have played prominent roles in revealing the mechanism of induced mutations. Differences in the radiosensitivity of various crops have been investigated [1–3]. However, the molecular mechanisms responsible for radiosensitivity are currently unclear, making research in this area vital. Investigations in animals and human have revealed a link between cell radiosensitivity and variations in free radicals, oxidative stress, and DNA repair mechanisms [4–10]. Therefore, it is important to further explore radiosensitivity and its molecular determinants. Such information would be useful for predicting and modulating radiosensitivity.

Organisms exposed to irradiation are induced to produce reactive oxygen species (ROS), which can give rise to DNA double-strand breaks (DSBs), which in turn can affect proteins. However, some plants, during the long process of evolution, have developed the ability to withstand ionizing radiation (IR) stress. Under IR stress, such plants can activate ROS removal systems and DNA repair systems. The former includes enzyme and non-enzyme based systems. The enzyme systems utilize amongst others, SOD, GPX and catalase to quickly remove ROS. Non-enzymatic systems utilize, amongst others, the surfhydryl of glutathione (GSH), ascorbic acid and the carotenoqesls to quench oxyradicals [10–16]. Currently, it is widely accepted that oxidative stress is involved in the pathogenesis of many diseases, including various cancers and degenerative disorders in animals [17]. In our study, we assess the effects of ROS on hexaploid wheat, as well as its total antioxidative capacity (T-AOC). DSBs caused by IR is disastrous to both cells and organisms. It can trigger chromosomal rearrangements, aneuploidy and the loss of segments of chromosomes. If not followed by rapid and successful repair, cell and whole organism death can be infamous result [18–21]. In eukaryotes, Non-homologous end-joining (NHEJ) is the most important DSB repair pathway [22–25]. ku70/ku80 heterodimers recognize DSBs and bind to them, forming complexes. These recruit DNA-dependent protein kinase catalytic subunits which initiate the NHEJ repair process [26–29], Errors in or DNA mutations involving this process can lead to failure of repair and so increase the sensitivity of the organism to ionizing radiation [30]. In our study, defects in the *TaKu70* gene may be one reason for the high sensitivity of our HY1 strain. Under normal circumstances, alternative splicing of pre-mature RNA is an important process utilized by eukaryotes to produce all kinds of protein forms from a single gene [31–34]. This can enhance protein diversity and regulates some process in plants [35–37]. Some studies have suggested that the alternative splicing of single genes can create small amounts of protein isoforms in plants. In rice, approximately 68.3% of genes create only one isoform. However, among all splicing types, intron retention is common [38–40]. In wheat, investigations are ongoing. However, with regards mechanisms and function, there seem to be few surprises [41]. Evidence suggests that weak splice sites, shorter introns and lower density splicing enhancers intron retention [42–43]. Therefore, their retention suggests the occurrence of missplicing, caused by problems with the splicing machinery [44]. In addition, alternative splicing regulates the expression of certain critical genes [45–48].

Any defect of alternative splicing can cause severe problems to the organism as a result of changes in their protein composition. Frame shifts are one consequence of miss-splicing [36]. In Arabidopsis 42% of fame shift events create a premature stop codon; in rice, the frequency is 36% [32]. In our study, intron retention was found in the *TaKu70* gene. This may reflect splicing errors in the pre-mRNA splicing process, prematurely stopping the expression of *TaKu70*. Ultimately, defective TaKu70 protein might be produced.

Phenotype studies of ku70-defects in DNA repair mechanisms have been carried out in fungi, yeasts and animals [49]. Disruption of Ku70 in mouse embryonic stem cells results in markedly increased sensitivity to ionizing radiation [50]. Ku70-deficient mice are approximately half the size of control mice, and their fibroblasts are sensitive to ionizing radiation and display premature senescence associated with the accumulation of nondividing cells [51]. In Arabidopsis, *ku* mutants can be associated with telomere elongation [52]. In our study, *TaKu70* defective wheat was highly sensitivity to γ radiation. This produced severe phenotypic changes compared to the controls.

We previously cloned *Ku70* and *Ku80* in wheat, which were designated *TaKu70* and *TaKu80*, respectively [53–55]. The functions of the two genes, as well as the encoded protein, have been investigated [55]. We previously selected the hexaploid wheat variety HY1, which exhibited the highest sensitivity to γ-irradiation among the 63 wheat genotypes examined, whereas wheat variety J411 exhibited insensitivity to γ-irradiation[56]. In this study, the increased radiosensitivity of this variety allowed us to analyze the combined effects of an exogenous agent and IR on plants[14]. The results of this study may help shed light on the mechanism underlying radiosensitivity in wheat.

## Materials and Methods

### γ-irradiation and free radical contents assay

The moisture content of the dry seeds of HY1 and J411 was balanced with glycerin and water ratio of 1:1 to up to 13% and they were then irradiated by gamma rays at dosages of 100, 150 and 250 Gy (7 Gy/min; The Department of Radiation at Peking University, Beijing, China). The free radical contents under each dosage were immediately examined using electron spin resonance apparatus (ESR, E-Scan, BRUKER, SC0340, Germany), and various parameters were obtained, namely Food, Marker, g1-value, g2-value, Frequency. The seeds under each dosage were randomly examined five times. The Food parameters values, which represent variations in free radicals, were normalized using the formula: Foodn = Food × 400000 / Marker. Foodn values can take the place of relative free radical contents values.

### Plant materials and cultivation

All wheat seeds were kindly provided by the Chinese Academy of Agricultural Sciences. Prior to placing them on a hydroponics shelf for seedling growth, the dried seeds were immersed in distilled water for germination for 16 hours with a rate of 50 seeds/15 mL water (using three replicates per dosage). Germinated seeds were placed on a hydroponics shelf under controlled conditions (16 h light/12 h dark, temperature: 25°C, relative humidity: 78%). Leaves were harvested on the day 5 for DNA and RNA extraction.

### Total antioxidative capacity (T-AOC) assay

The irradiated seeds were soaked in distilled water for 16 h (using three replicates per dosage) and transferred to the germination apparatus under constant conditions. Leaf samples (0.05 g) were collected on day 5, instantly placed into 2 mL EP tubes containing 500 μL 0.9% normal saline and ground into a powder using a tissue grinder apparatus (30 Hz/s, 60 s). The samples were centrifuged for 300 s at 5,000 rpm, and 300 μL of supernatant was transferred to a fresh 1.5 mL centrifuge tube (on ice). The T-AOC assay was carried out using a T-AOC reagent kit, and the OD value at 520 nm was measured following the manufacturer’s instructions using the formulaT − AOC = (ODu − ODc) / 0.01 / 30 * N / CProt.

### DNA isolation, PCR amplification and sequencing

Genomic DNA was obtained from a pool of DNA extracted from ten HY1 and J411 leaves using a Caliper workstation and a DNA Secure Plant Kit. To determine the source of the retained fragment in the cDNA, specific primers were designed (25AER: 5’ GGCACTGCTGCGTAAAGG 3’, 25AEF: 5’ TCACCAGCAGATGGCACG 3’) based on the A, B and D genome sequences [55]. For amplification and sequencing of the 879 bp gene containing the 133 bp retention fragment, primers were designed approximately 20 to 50 bp upstream and downstream of the coding region. For analysis of the region, the fragments of accessions were amplified using the proofreading polymerase Phusion (Finnzymes) and subjected to TA clone prior to sequencing. The positive clones were directly used for sequencing.

### RNA isolation, cDNA synthesis and real-time PCR

RNA was isolated with TRNzol^A+^solution. For each accession, three biological replicates were performed, and 1 μg RNA was reversely transcribed using a Transcriptor High Fidelity First-strand cDNA Synthesis Kit (Roche, Version 6, Germany). For the *TaKu70* gene, 10 accessions were selected for RT-PCR and TA cloning. For each accession, 72 positive clones were selected for sequencing and analyses. Primary analyses revealed alternative splicing in the cDNA fragment from the HY1 accessions, which was confirmed using a validation protocol. Data analysis, sequence alignment and were performed with BioEdit version 7.05 for each accession, and all variable sites were checked manually during the construction of a sequence contig. All sequences were manually aligned to the reference sequence. RNA was reversely transcribed and used at 1 μg per real-time-PCR run in a 10 μL reaction volume using SsoFast™ EvaGreen Supermix and a C1000^™^ thermal cycler; each biological replicate included three technical replicates. Expression was normalized to the *Actin* and *18s* genes. The primer sequences are shown in S1 Table. A CFx96^™^ Real-time System was used for analysis.

### Data analysis

Statistical analysis was performed using SPSS 16.0, with one-way ANOVA performed to test the significance of differences when more than two groups were involved. Values were considered significantly different if (P < 0.05). Comprehensive data analysis was performed using Heml1.0 software.

## Results

### Variation in free radical contents

**Fig 1:**
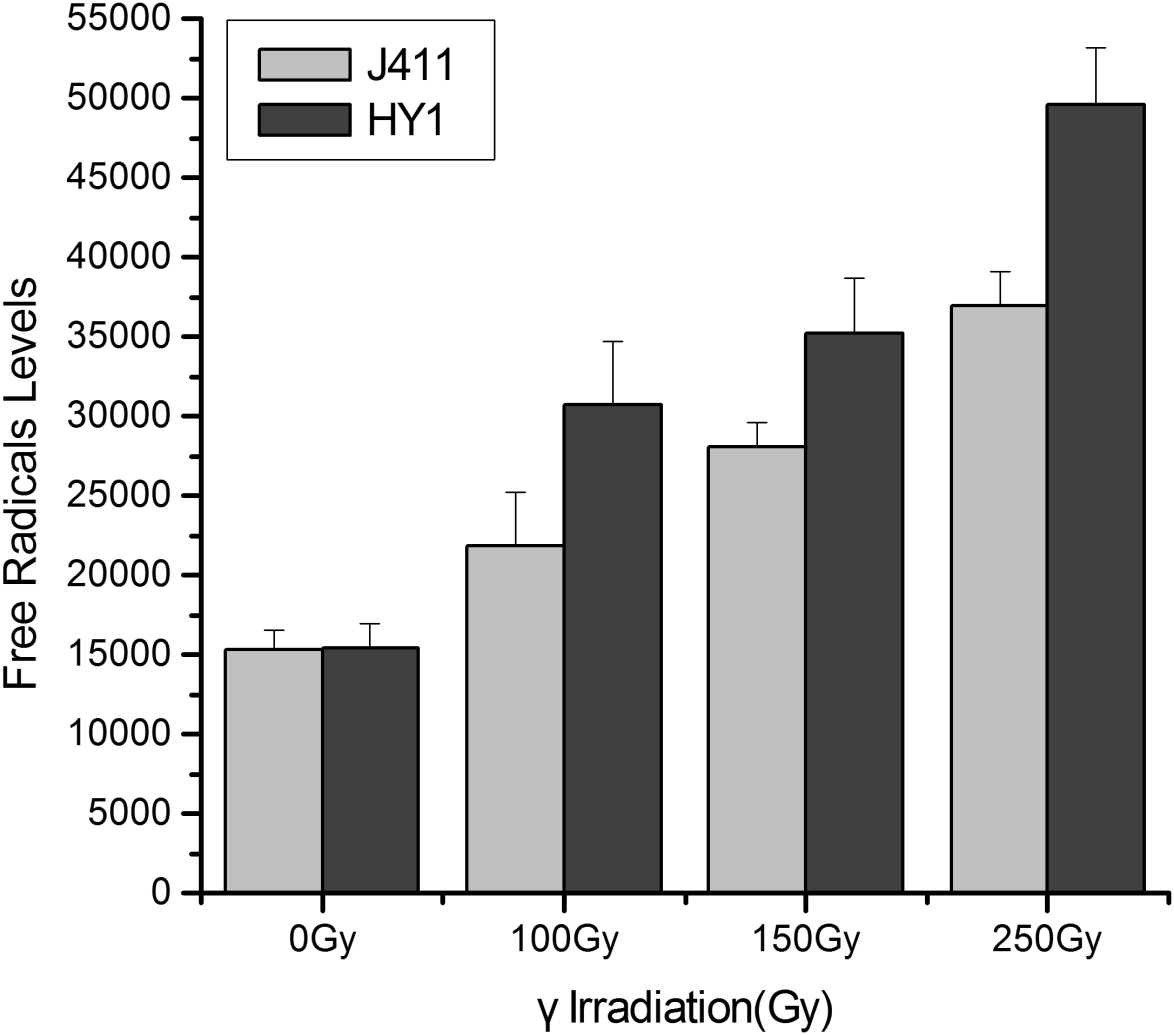
Effect of gamma irradiation on free radical levels. The X-axis represents the treatment dosage, including 0 Gy, 100 Gy, 150 Gy and 250 Gy. The Y-axis represents the free radical levels. Dark gray bars indicate free radical contents in HY1, light gray bars represent free radical contents in the J411 variety. Significant differences between treatment groups and the control groups in the HY1and J411 variety were analyzed by spass 16.0 (P < 0.05).

To investigate the oxidative stress caused by gamma irradiation, we measured the free radical contents in HY1 wheat and the control variety J411 in response to various dosages of IR (Fig 1). The relative free radical content increased significantly in a dose-dependent manner. At 250 Gy, the relative free radical levels were significant increased in these plants, reaching more than 3.0-fold control dosage levels (under 0 Gy treatment). The free radical contents were more than 2.0-fold control levels under 100 Gy treatment and 2.5-fold control levels under 150 Gy treatment. These levels were significantly higher in the treatment groups than in the control dosage group (p < 0.05). In the control variety J411, the relative free radical contents increased in a dose-dependent manner, but the upward trend was slower. The basal levels of free radicals in HY1 and J411 were similar.

### Total antioxidant capacity (T-AOC)

**Fig 2:**
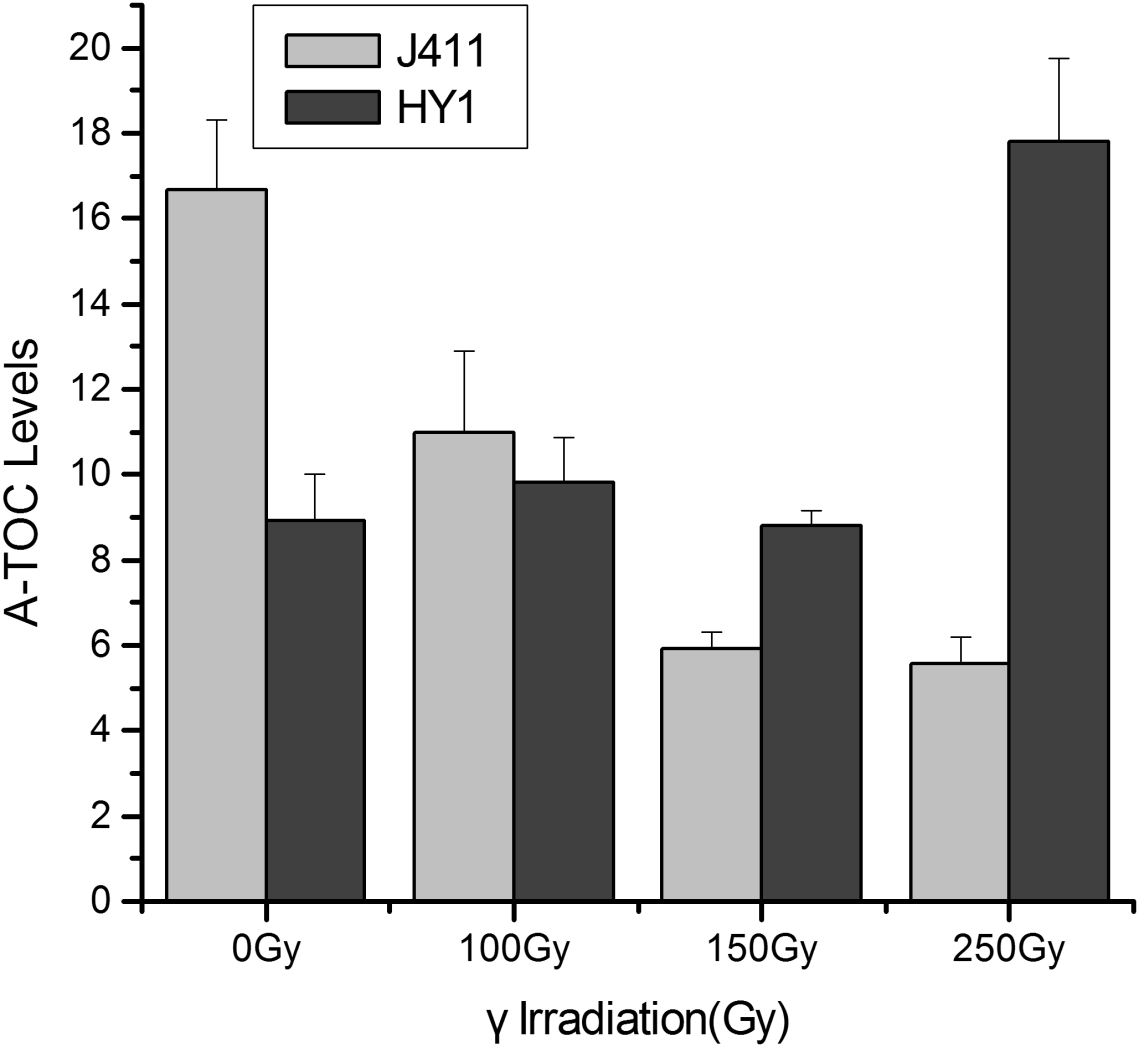
Effect of gamma irradiation on T-AOC in HY1 and J411 wheat. The X-axis represents the treatment dosage, including 0 Gy, 100 Gy, 150 Gy and 250 Gy. The Y-axis represents T-AOC levels. Dark gray bars represent T-AOC values in HY1, and light gray bars represent those in J411. Significant differences between treatment groups and the control groups in the HY1and J411 variety were analyzed by spass 16.0 (P < 0.05).

To analyze the roles of enzymatic and non-enzymatic components in HY1 and the control variety J411, namely, the T-AOC of these plants, we used a total antioxidant capacity kit to measure OD values at 520 nm, which revealed T-AOC values under each dosage of IR (Fig 2). In HY1, the T-AOC values increased significantly with increasing gamma irradiation dosage. Under 250 Gy treatment, T-AOC reached more than two-times the levels measured under the control dosage levels. At lower doses, however, increases in T-AOC values were drastically lower, approaching the values of the control dosage group, The basal T-AOC was higher in the control variety J411 than in HY1, whereas at 100 Gy and 150 Gy, these values were consistent in the two varieties, and at 250 Gy, this value was lower in J411 than the HY1.

### DNA repair-related gene expression

**Fig 3:**
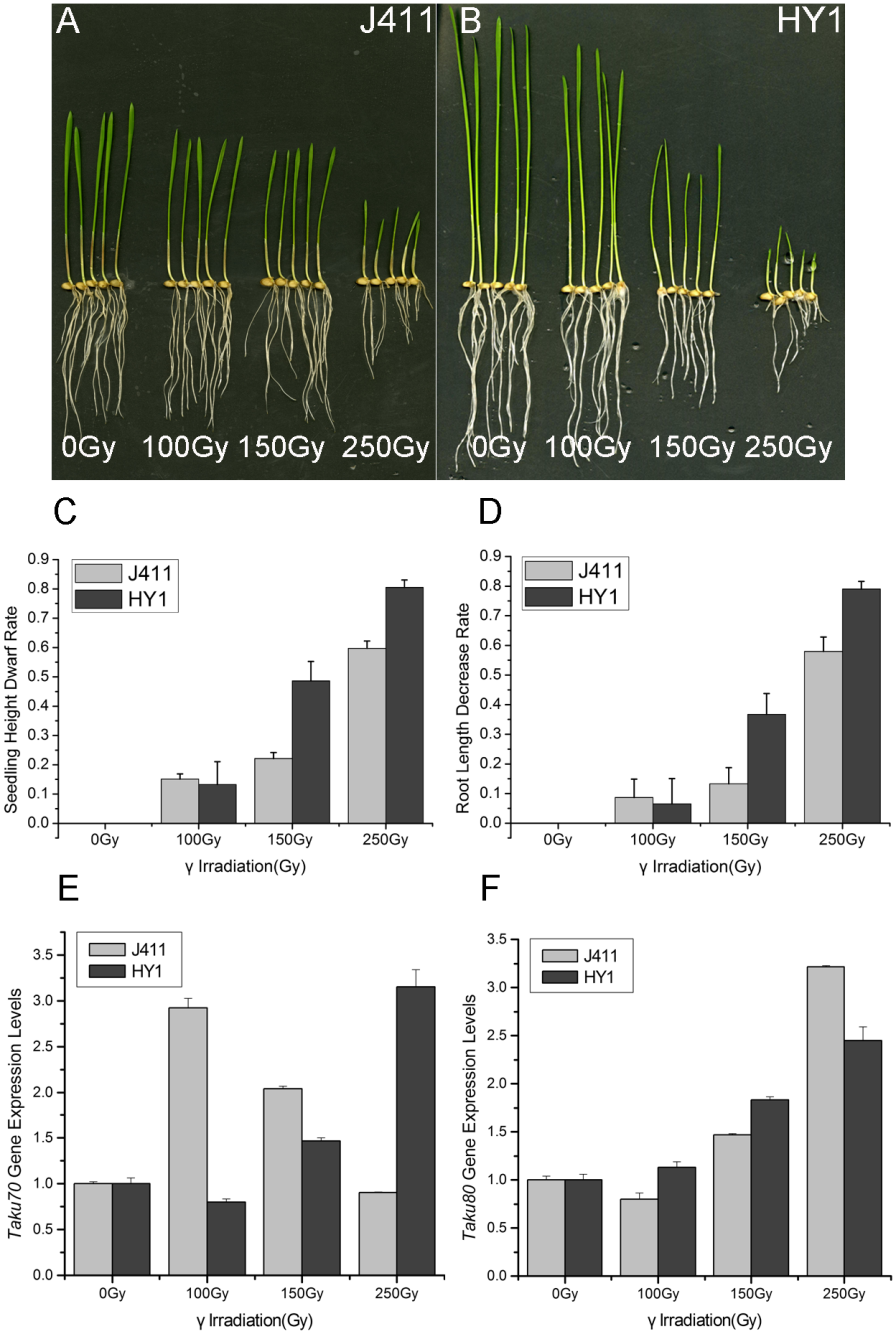
Effect of gamma irradiation on DNA repair-related genes *TaKu70* and *TaKu80 and plant phenotypes*. (A, B) Photographs of HY1 and J411 plants under different dosages of γ-irradiation. Seedling height and root length decreased significantly with increasing gamma irradiation dosage more quickly in HY1 than in J411. (C, D) Histogram analysis of variation rate of root length and seedling height in HY1 and J411. The X-axis represents the treatment dosage, including 0 Gy, 100 Gy, 150 Gy and 250 Gy. The Y-axis represents the variation rate of root length and seedling in HY1 and J411. Significant differences were analyzed by spass 16.0 (P < 0.05) (E, F)The X-axis represents the treatment dosage, including 0 Gy, 100 Gy, 150 Gy and 250 Gy. The Y-axis represents *Taku70* gene expression level. Dark gray bars indicate *Taku70* and *Taku80* gene expression values in HY1, and light gray bars indicate those in J411. Significant differences between treatment groups and the control groups in the HY1and J411 variety were analyzed by spass 16.0 (P < 0.05).

To investigate changes in the expression of the DNA repair-related genes *TaKu70* and *TaKu80* in response to gamma irradiation, we monitored the expression of these genes in IR-treated HY1 and the control variety J411 (Fig 3E and 3F). In HY1, under high doses of gamma irradiation, both genes were significantly induced, especially *TaKu70*; at 250 Gy, the expression level of this gene was more than three-times that of the control(0Gy). However, under 100 Gy treatment, both genes were only slightly induced. The phenotypes of HY-1 in response to 0, 100, 150, and 250 Gy gamma irradiation are shown in Fig 3B, Seedling height and root length significantly decreased with increasing gamma irradiation dosage. while in the control variety J411, *TaKu70* was significantly induced at dosages of 100 Gy and 150 Gy. At 100 Gy, the expression level of this gene was more than two-times that of the control dose (0 Gy). *Taku80* expression levels were consistent with the levels detected in HY1. The phenotypes of J411 in response to IR are shown in Fig 3A. Seedling height and root length decreased slightly with increasing gamma irradiation dosage. Histogram analysis of seedling height and root length in HY1 and the control variety J411 is shown in Fig 3C and 3D. Seedling height decreased significantly with increasing gamma irradiation dosage in both HY1 and J411, but the reduction was slower in J411 than in HY1 (Fig 3C and 3D)

### Intron retention in *TaKu70*

**Fig 4:**
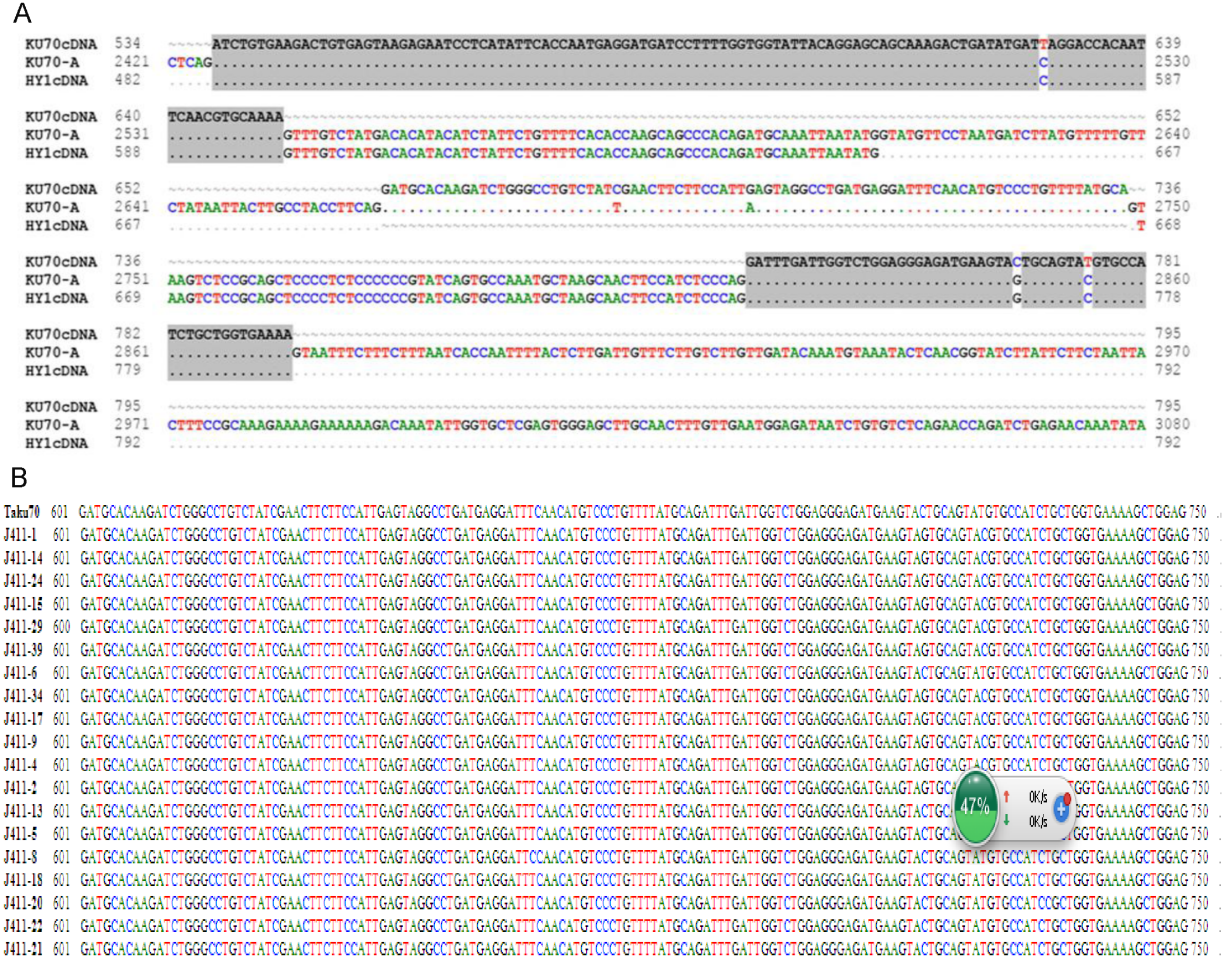
Analysis of the alternative splicing, namely, intron retention. (A) The first region highlighted in gray represents the sixth exon., and the second represents the eighth exon. Ku70-cDNA and the ku70 –A sequence were obtained from the Chinese spring variety cloned by our team as the standard comparison sequence. (B) J411-1, J411-14, J411-24 s equence, and so on represent 19 clone replicates from J411.

To explore the transcriptional regulation of *TaKu70*, which affects the radiosensitivity of HY1 and the control variety J411, we cloned *TaKu70* cDNA sequences (S2 Fig) from both varieties and subjected the mRNA sequences to alignment (Fig 4A). In HY1, cDNA sequence alignment (S2 Fig) showed that a 133 bp fragment located between 601 bp and 733 bp was retained in its mRNA sequence. A comparison between HY1 *TaKu70* cDNA and the wild-type *TaKu70* sequence (S1 Fig) showed that the retained 133 bp fragment was derived from the sixth intron (67 bp) located between 2,544 bp and 2,610 bp and the seventh intron (66 bp) located between 2,750 bp and 2,815 bp in the A genome (Fig 4A). In J411, 19 clone replicates were carried out, and is no retention was detected in its mRNA (Fig 4B, S4 Fig).

### Amino acid analysis of encoded *TaKu70* protein

**Fig 5:**
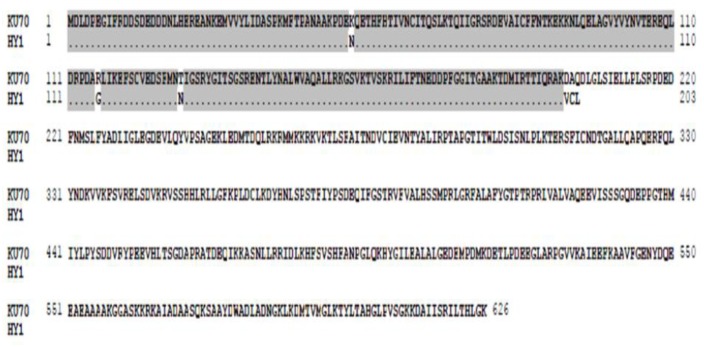
The effect of intron retention on the encoded protein. *TaKu70* encodes a 626 amino acid protein. Gray highlighting represents the protein encoded by the mRNA harboring intron retention in HY1.

As shown in Fig 5, normal *TaKu70* mRNA encodes a functional protein, TaKu70, containing 626 amino acid residues. However, *TaKu70* exhibiting intron retention encodes a non-functional TaKu70 protein of only 200 amino acid residues (S3 Fig). Whether this protein is actually produced and functional remains unclear.

### Integrated analysis of all data

**Fig 6:**
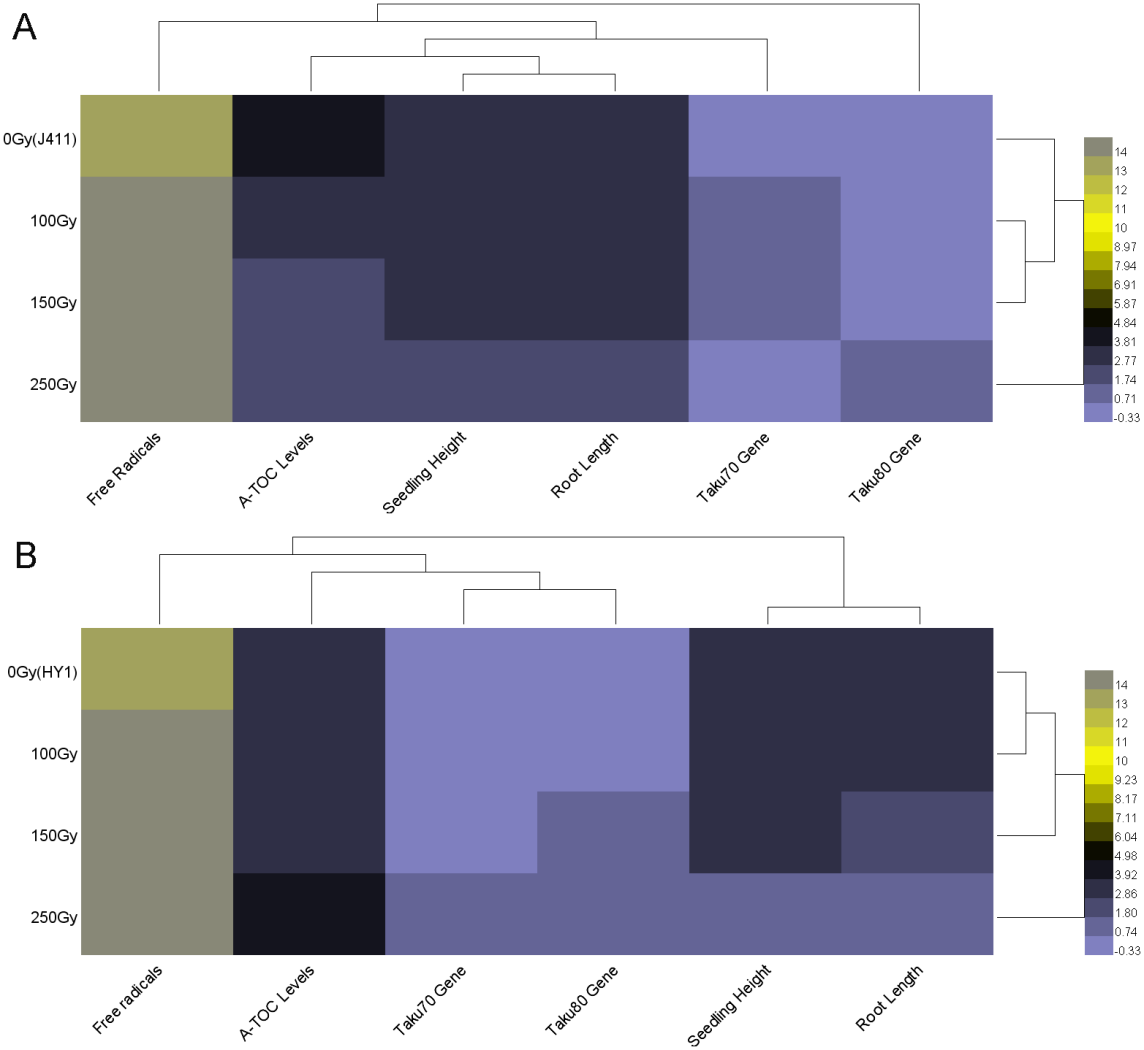
Heat map analysis of all data. The abscissa represents free radical levels, A-TOC levels, seedling height, root length, *TaKu70* and *TaKu80* expression levels. The ordinate represents different dosages of γ-irradiation. The primary data were LOG_2_ transformed using Heml1.0 software. The color variation represents different values.

Heml1.0 software was used to construct a heat map of all of the data. As shown in Fig 6, the clustering results suggest that the phenotypic variation is related to *TaKu70* expression and T-AOC levels. Phenotypic variation is a standard measure of radiosensitivity. In the J411 variety, the A-TOC levels, *TaKu70* expression levels, phenotypes and radicals levels were clustered together then *TaKu80* expression levels. On the Y-axis, 100 Gy and 150 Gy were clustered together (Fig 6A). In the HY1 variety, *TaKu70* and *TaKu80* expression levels, T-AOC and radicals levels were clustered together, which ultimately influence the phenotype. On the Y-axis, 0 Gy and 100 Gy were clustered then150 Gy (Fig 6B).

## Discussion

The molecular mechanisms responsible for radiosensitivity are currently unclear, making this topic a vital area of research. In the current study, we explored the molecular determinants of radiosensitivity. We chose the hexaploid wheat variety HY1 and the variety J411 as a control: the former was previously found to be the most susceptible variety to gamma irradiation and the latter is not radiosensitive to gamma irradiation among the 63 wheat genotypes examined [55–56]. To date, several studies examining the effects of gamma irradiation have been carried out in terrestrial organisms and some aquatic vertebrates [57–59]. Some studies suggest that different levels of susceptibility to radiation between organisms are likely due to differences in DNA content, repair processes, and cell cycle kinetics [60]. In the current study, we found that the high radiosensitivity of HY1 may be linked to the DNA repair process and A-TOC levels (Fig 6). However, the underlying mechanisms that function in organisms upon gamma irradiation have not been investigated in detail.

Free radicals play an important role in radiosensitivity. We therefore assessed the free radicals levels of the control variety J411 and HY1, finding that both of these levels increased upon exposure to radiation, but the increase was more rapid in HY1 than in J411 (Fig 1). This result suggests that under the same γ-irradiation conditions, J411 may produce fewer free radicals than HY1. The accumulation of free radicals can lead to DSBs and a variety of molecular effects, including preventing cell division, aging and apoptosis [61]. Organisms utilize enzymatic and non-enzymatic systems to counteract the effects of free radicals in an attempt to maintain cellular homeostasis [62]. We therefore examined the T-AOC of enzymatic and non-enzymatic antioxidants using a total antioxidant capacity kit. HY1 had lower basal A-TOC levels than J411. The HY1 cells exhibited lower T-AOC under lower doses of gamma irradiation (100 Gy and 150 Gy), but quickly increased at high doses (250 Gy). While in J411, the basal T-AOC level was higher than that of HY1, and the T-AOC levels decreased with increasing irradiation (Fig 2). The cause of this difference is currently unclear. Perhaps the high dose of γ-irradiation induced some enzymatic activities, and the primordial T-AOC substance can remove the induced T-AOC substance in the J411 variety. These results all suggest that enzymatic and non-enzymatic antioxidants play main role in resisting irradiation stress in the two varieties. Additionally, the enzymatic and non-enzymatic system comprises several components, such as SOD, GSH, CAT, GR, GST, GPX, and so on [63]. The total antioxidant enzyme activities displayed in Fig 2 may not represent the effect of each individual enzyme. However, interestingly, the expression of the DNA repair-related gene *TaKu70* (Fig 3E) dramatically increased under 100 Gy gamma irradiation and then began to decline in J411. In addition, the *TaKu70* mRNA sequence lacked a retention segment (Fig 4B), suggesting that J411 *TaKu70* mRNA encodes the normal protein and has completed the DNA repair progress, which may be an important cause of the low radiosensitivity of this variety. In HY1, *TaKu70* was significantly upregulated at 150 Gy and 250 Gy. However, segment retention occurred in the mRNA of HY1, which may encode a nonfunctional protein (Fig 4A). This segment retentionb may be an important cause of the radiosensitivity of HY1. These results suggest that the DNA repair system may greatly contribute to the varied radiosensitivity of J411 and HY1. The different A-TOC levels in the two varieties likely contributes to this difference as well. Comprehensive heat map analysis of all of the data (Fig 6A and 6B) helped confirm the above-mentioned results. Some researchers have proposed that gene transcription levels are a reliable early signal for detecting physiological changes under environmental stress [64–65]. Elucidating the expression patterns of specific genes would be helpful for better understanding the underlying molecular mechanisms of radiosensitivity upon γ-irradiation. *Ku70* and *Ku80* are induced by γ-irradiation in a dose-dependent manner in the marine copepod *Paracyclopina nana* [63]. In human, Ku70, and Ku80 are key components of the DSBs repair process, as they ligate the broken ends of DNA in the absence of homologous templates [66]. Thus, the increased expression of *TaKu70* and *TaKu80* in gamma-irradiated HY1 and J411 implies that these genes are closely related to the enhanced DNA repair process that functions to recover oxidative stress-induced cellular damage in these plants.

RNA sequencing confirmed that intron retention occurred in *TaKu70* mRNA (Fig 4A) in HY1, which may also help explain the increased radiosensitivity of this genotype. The 133 bp fragment retained in the mRNA would lead to the production of a 200 amino acid, non-functional TaKu70 protein (Fig 5). Thus, DNA repair of DSBs would be weakened or inhibited, which may contribute to the high radiosensitivity of the HY1 variety. Disruption of Ku70 in mouse embryonic stem cells results in severely increased sensitivity to ionizing radiation [50]. The aborted DNA repair process might cause the phenotypic variation observed in HY1 (Fig 3A), Ku70-deficient mice are approximately 50% the size of the control [51], the phenotypic effects of different doses of radiation were reconfirm in our experiments (Fig 3A and 3B). However, our knowledge of the physiological relevance of this important post-transcriptional regulatory mechanism in plants is quite limited. The current study provides functional evidence that alternative splicing plays a important role in plant responses to environmental stress [41]. Our data open up the possibility for further study of a probable link between alternative splicing and hypersensitivity to γ-ionizing radiation in plants,

In summary, our results suggest that there is a correlation between radiosensitivity and intron retention, as well as activation of the antioxidant and DNA repair systems, in HY1. No previous studies have investigated the radiosensitivity mechanism in other plants. In this study, we found that the antioxidant and DNA repair systems were induced by gamma irradiation to mitigate damage from free radicals. Additionally, alternative splicing, namely intron retention, might contribute to the radiosensitivity of HY1. Further evidence is needed to confirm the correlation between radiosensitivity and intron retention in hexaploid wheat.

## Supporting Information

S1 Fig The comparison of HY1 TaKu70 cDNA, *Ku70-A* and *TaKu70* cDNA. Ku70-A genome and Taku70cDNA was cloned from the Chinese spring. HY1Taku70 cDNA was cloned from HY1.
(PDF)

S2 Fig The comparsion of HY1 TaKu70 cDNA and *TaKu70* cDNA. 133 bp fragment located between 601 bp and 733 bp was retained in the mRNA sequence in HY1.
(PDF)

S3 Fig Amino acid analysis of encoded TaKu70 protein. **T**aku70 encodes a 626 amino acid residues protein. The detained mRNA encoded 200 amino acid residues protein.
(PDF)

S4 Fig The original sequences of the 19 clone repeats of *Taku70* gene mRNA from J411 variety. (PDF)

S1 Table primer Squence used in the Quantitative Real-time PCR. (PDF)
