# Correction: Factors Affecting the Radiosensitivity of Hexaploid Wheat to γ-Irradiation: Radiosensitivity of Hexaploid Wheat (*Triticum aestivum* L.)

**DOI:** 10.1371/journal.pone.0165187

**Published:** 2016-10-20

**Authors:** The PLOS ONE Staff

## Notice of Republication

This article was republished on September 6, 2016, to correct an error in the article title that was introduced during the typesetting process. The publisher apologizes for the error. Please download this article again to view the correct version. The originally published, uncorrected article and the republished, corrected article are provided here for reference.

## Supporting Information

S1 File Originally published, uncorrected article. (PDF)

S2 File Republished, corrected article. (PDF)
