# Supplementary material for: Factors Affecting the Radiosensitivity of Hexaploid Wheat to γ-Irradiation: Radiosensitivity of Hexaploid Wheat (Triticum aestivum L.)
Source: PLoS One. 2016 Aug 23;11(8):e0161700. doi: 10.1371/journal.pone.0161700 (PMC4995049; doi:10.1371/journal.pone.0161700)
Supplement: S1 Fig — Ku70-A genome and Taku70cDNA was cloned from the Chinese spring. HY1Taku70 cDNA was cloned from HY1. (PDF) [file pone.0161700.s001.pdf]

```
                  10        20        30        40        50        60        70        80
          ....|....|....|....|....|....|....|....|....|....|....|....|....|....|....|....|
KU70cDNA  TTCCCCACGCACCCCCTTCTTCCCCGCGCGAGTGCTAGTTCCGGTCGGCGCCATGGACCTGGACCCCGAGGGCATCTTCC 80
KU70-A    ................................................................................ 80
HY1 cDNA  ~~~~~~~~~~~~~~~~~~~~~~~~~~~~~~~~~~~~~~~~~~~~~~~~~~~~............................ 28

                  90       100       110       120       130       140       150       160
          ....|....|....|....|....|....|....|....|....|....|....|....|....|....|....|....|
KU70cDNA  GCGACGACAGCGACGAGGACGACGACAACCTCCAT~~~~~~~~~~~~~~~~~~~~~~~~~~~~~~~~~~~~~~~~~~~~~ 115
KU70-A    ...................................GTACGCTCCCTGCTCCGCCTTCCTTTTCCCCATTTTCCATCTTGG 160
HY1 cDNA  ...................................~~~~~~~~~~~~~~~~~~~~~~~~~~~~~~~~~~~~~~~~~~~~~ 63

                 170       180       190       200       210       220       230       240
          ....|....|....|....|....|....|....|....|....|....|....|....|....|....|....|....|
KU70cDNA  ~~~~~~~~~~~~~~~~~~~~~~~~~~~~~~~~~~~~~~~~~~~~~~~~~~~~~~~~~~~~~~GAGAGGGAGGCCAACAAG 133
KU70-A    GTTCGATTGCTGCGTGCGTCGCGATTTGTTCTGATGCTAGCGTGTGCTGGTTCTGGATGCAG.................. 240
HY1 cDNA  ~~~~~~~~~~~~~~~~~~~~~~~~~~~~~~~~~~~~~~~~~~~~~~~~~~~~~~~~~~~~~~.................. 81

                 250       260       270       280       290       300       310       320
          ....|....|....|....|....|....|....|....|....|....|....|....|....|....|....|....|
KU70cDNA  GAGATGGTCGTCTACCTCATAGACGCCTCGCCCAAGATGTTCACACCCGCCAACGCCGCCAAG~~~~~~~~~~~~~~~~~ 196
KU70-A    ............................................C..................GTGAAGCACAAGCAGCT 320
HY1 cDNA  ............................................C..................~~~~~~~~~~~~~~~~~ 144

                 330       340       350       360       370       380       390       400
          ....|....|....|....|....|....|....|....|....|....|....|....|....|....|....|....|
KU70cDNA  ~~~~~~~~~~~~~~~~~~~~~~~~~~~~~~~~~~~~~~~~~~~~~~~~~~~~~~~~~~~~~~~~~~~~~~~~~~~~~~~~ 196
KU70-A    TATATGCGCATAGTTTCACAGCCCCAGAGCATTGTTGAGTCTGCAATTGAGTTATATATAACATGTACTTTGACCATAAC 400
HY1 cDNA  ~~~~~~~~~~~~~~~~~~~~~~~~~~~~~~~~~~~~~~~~~~~~~~~~~~~~~~~~~~~~~~~~~~~~~~~~~~~~~~~~ 144

                 410       420       430       440       450       460       470       480
          ....|....|....|....|....|....|....|....|....|....|....|....|....|....|....|....|
KU70cDNA  ~~~~~~~~~~~~~~~~~~~~~~~~~~~~~~~~~~~~~~~~~~~~~~~~~~~~~~~~~~~~~~~~~~~~~~~~~~~~~~~~ 196
KU70-A    TTCTCCAAGTGGCACAACTTACAAGTAGTATGCCTGGTTTTCGGCAGAAGCAAAGTAGGTAGCTAGTCTTTCCAGGGTTT 480
HY1 cDNA  ~~~~~~~~~~~~~~~~~~~~~~~~~~~~~~~~~~~~~~~~~~~~~~~~~~~~~~~~~~~~~~~~~~~~~~~~~~~~~~~~ 144

                 490       500       510       520       530       540       550       560
          ....|....|....|....|....|....|....|....|....|....|....|....|....|....|....|....|
KU70cDNA  ~~~~~~~~~~~~~~~~~~~~~~~~~~~~~~~~~~~~~~~~~~~~~~~~~~~~~~~~~~~~~~~~~~~~~~~~~~~~~~~~ 196
KU70-A    GCATCTGATCTAGGACTCTAGGTTATTTTTTAGACTGAATGGTTTTGGGGAATACGATGGTAAGGGTAGGAGCTTGTTGT 560
HY1 cDNA  ~~~~~~~~~~~~~~~~~~~~~~~~~~~~~~~~~~~~~~~~~~~~~~~~~~~~~~~~~~~~~~~~~~~~~~~~~~~~~~~~ 144

                 570       580       590       600       610       620       630       640
          ....|....|....|....|....|....|....|....|....|....|....|....|....|....|....|....|
KU70cDNA  ~~~~~~~~~~~~~~~~~~~~~~~~~~~~~~~~~~~~~~~~~~~~~~~~~~~~~~~~~~~~~~~~~~~~~~~~~~~~~~~~ 196
KU70-A    AAATTTGCCGATATTTAGTTTGTGTGAAGTTCCATGGTTATAATCATTTACTAGGTAATGTAGCATTTTGGTTTTGTTGT 640
HY1 cDNA  ~~~~~~~~~~~~~~~~~~~~~~~~~~~~~~~~~~~~~~~~~~~~~~~~~~~~~~~~~~~~~~~~~~~~~~~~~~~~~~~~ 144

                 650       660       670       680       690       700       710       720
          ....|....|....|....|....|....|....|....|....|....|....|....|....|....|....|....|
KU70cDNA  ~~~~~~~~~CCAGATGAAAAGCAGGAGACACATTTCCATACCATAGTGAACTGCATCACGCAGTCTCTGAAGACGCAGAT 267
KU70-A    TTCTTGCAG........G..T.....................................................A..... 720
HY1 cDNA  ~~~~~~~~~........G..T.....................................................A..... 215

                 730       740       750       760       770       780       790       800
          ....|....|....|....|....|....|....|....|....|....|....|....|....|....|....|....|
KU70cDNA  TATCGGGAGATCCCGTGATGAAGTTGCAATATGCTTCTTTAACACC~~~~~~~~~~~~~~~~~~~~~~~~~~~~~~~~~~ 313
KU70-A    .......................................C......GTAAGTTGCTACTCATAGTTGTAAGCTTTAACTC 800
HY1 cDNA  .......................................C......~~~~~~~~~~~~~~~~~~~~~~~~~~~~~~~~~~ 261

                 810       820       830       840       850       860       870       880
          ....|....|....|....|....|....|....|....|....|....|....|....|....|....|....|....|
KU70cDNA  ~~~~~~~~~~~~~~~~~~~~~~~~~~~~~~~~~~~~~~~~~~~~~~~~~~~~~~~~~~~~~~~~~~~~~~~~~~~~~~~~ 313
KU70-A    GATGCTTGATTAGTTATTAGTTAATTTATTCGCTGGTTCAAGTCCTCTGATTTTACTGCAAGTGATTGTTCGGCAAAGAC 880
HY1 cDNA  ~~~~~~~~~~~~~~~~~~~~~~~~~~~~~~~~~~~~~~~~~~~~~~~~~~~~~~~~~~~~~~~~~~~~~~~~~~~~~~~~ 261

                 890       900       910       920       930       940       950       960
          ....|....|....|....|....|....|....|....|....|....|....|....|....|....|....|....|
KU70cDNA  ~~~~~~~~~~~~~~~~~~~~~~~~~~~~~~~~~~~~~~~~~~~~~~~~~~~~~~~~~~~~~~~~~~~~~~~~~~~~~~~~ 313
KU70-A    GAGGTTTTCGAGTTTCAACCTGTTAGTTGAGACCTTAATCTTCGCTGGTGTATTGCTCTCTGTGACAACTTTGCTTCCTA 960
HY1 cDNA  ~~~~~~~~~~~~~~~~~~~~~~~~~~~~~~~~~~~~~~~~~~~~~~~~~~~~~~~~~~~~~~~~~~~~~~~~~~~~~~~~ 261

                 970       980       990      1000      1010      1020      1030      1040
          ....|....|....|....|....|....|....|....|....|....|....|....|....|....|....|....|
KU70cDNA  ~~~~~~~~~~~~~~~~~~~~~~~~~~~~~~~~~~~~~~~~~~~~~~~~~~~~~~~~~~~~~~~~~~~~~~~~~~~~~~~~ 313
KU70-A    GTTTTACTTGATAGATCTGCTGTTATCAGGCCTGATGAGTGGACACTGAACTTGAAGATTTCAATTATCTCTTTGAAATC 1040
HY1 cDNA  ~~~~~~~~~~~~~~~~~~~~~~~~~~~~~~~~~~~~~~~~~~~~~~~~~~~~~~~~~~~~~~~~~~~~~~~~~~~~~~~~ 261
```

```
                1050      1060      1070      1080      1090      1100      1110      1120
           ....|....|....|....|....|....|....|....|....|....|....|....|....|....|....|....|
KU70cDNA   ~~~~~~~~~~~~~~~~~~~~~~~~~~~~~~~~~~~~~~~~~~~~~~~~~~~~~~~~~~~~~~~~~~~~~~~~~~~~~~~~ 313
KU70-A     CACTTTCATCCATCAGCAAATGAACTGATTAGAACTGCCGGGTGTTGAAAAAATGACATGTTGCTATGATTTGTTCTTTT 1120
HY1 cDNA   ~~~~~~~~~~~~~~~~~~~~~~~~~~~~~~~~~~~~~~~~~~~~~~~~~~~~~~~~~~~~~~~~~~~~~~~~~~~~~~~~ 261

                1130      1140      1150      1160      1170      1180      1190      1200
           ....|....|....|....|....|....|....|....|....|....|....|....|....|....|....|....|
KU70cDNA   ~~~~~~~~~~~~~~~~~~~~~~~~~~~~~~~~~~~~~~~~~~~~~~~~~~~~~~~~~~~~~~~~~~~~~~~~~~~~~~~~ 313
KU70-A     GTCTATCAAACAGTTAAATGTGTGAAAATTTGAGATGAACTGTTTGTCTTGCACTATGTAGATTACTAATATCTGATAAG 1200
HY1 cDNA   ~~~~~~~~~~~~~~~~~~~~~~~~~~~~~~~~~~~~~~~~~~~~~~~~~~~~~~~~~~~~~~~~~~~~~~~~~~~~~~~~ 261

                1210      1220      1230      1240      1250      1260      1270      1280
           ....|....|....|....|....|....|....|....|....|....|....|....|....|....|....|....|
KU70cDNA   ~~~~~~~~~~~~~~~~~~~~~~~~~~~~~~~~~~~~~~~~~~~~~~~~~~~~~~~~~~~~~~~~~~~~~~~~~~~~~~~~ 313
KU70-A     ATTCTTTTTATGATGTTCCTTACAAAGTGCAACTTCCTCGAACACTTACGAAGTGGTACTGATATGCTATTGTTACATGG 1280
HY1 cDNA   ~~~~~~~~~~~~~~~~~~~~~~~~~~~~~~~~~~~~~~~~~~~~~~~~~~~~~~~~~~~~~~~~~~~~~~~~~~~~~~~~ 261

                1290      1300      1310      1320      1330      1340      1350      1360
           ....|....|....|....|....|....|....|....|....|....|....|....|....|....|....|....|
KU70cDNA   ~~~~~~~~~~~~~~~~~~~~~~~~~~~~~~~~~~~~~~AAAGAAAAGAAAAATTTACAGGAGCTGGCTGGTGTATATGTT 355
KU70-A     TACTCTTCTTTGATATAGCTATCACATAACTCTTGCAG.......................................... 1360
HY1 cDNA   ~~~~~~~~~~~~~~~~~~~~~~~~~~~~~~~~~~~~~~.......................................... 303

                1370      1380      1390      1400      1410      1420      1430      1440
           ....|....|....|....|....|....|....|....|....|....|....|....|....|....|....|....|
KU70cDNA   TACAATGTCACAGAAAGAGAGCAACTTGATAGACCTGATGCAAGACTGATTAAAGAATTTTCTTGTGTAGAAG~~~~~~~ 428
KU70-A     ...............C................G........................................GTAATGC 1440
HY1 cDNA   ...............C................G.........G..............................~~~~~~~ 376

                1450      1460      1470      1480      1490      1500      1510      1520
           ....|....|....|....|....|....|....|....|....|....|....|....|....|....|....|....|
KU70cDNA   ~~~~~~~~~~~~~~~~~~~~~~~~~~~~~~~~~~~~~~~~~~~~~~~~~~~~~~~~~~~~~~~~~~~~~~~~~~~~~~~~ 428
KU70-A     TACTTAATGAATTGTTCTTTTTGCACAGCTGATCAGAGTTTGAGTGATACACATGCTGGGCTACTTCCATGTAGCAGATT 1520
HY1 cDNA   ~~~~~~~~~~~~~~~~~~~~~~~~~~~~~~~~~~~~~~~~~~~~~~~~~~~~~~~~~~~~~~~~~~~~~~~~~~~~~~~~ 376

                1530      1540      1550      1560      1570      1580      1590      1600
           ....|....|....|....|....|....|....|....|....|....|....|....|....|....|....|....|
KU70cDNA   ~~~~~~~~~~~~~~~~~~~~~~~~~~~~~~~~~~~~~~~~~~~~~~~~~~~~~~~~~~~~~~~~~~~~~~~~~~~~~~~~ 428
KU70-A     TGTATATAACACTTGCAGAAAATTTGAAATTGTACAGTTGGATGGTTTACTTATACCATATCACACATGCATAAAAATCA 1600
HY1 cDNA   ~~~~~~~~~~~~~~~~~~~~~~~~~~~~~~~~~~~~~~~~~~~~~~~~~~~~~~~~~~~~~~~~~~~~~~~~~~~~~~~~ 376

                1610      1620      1630      1640      1650      1660      1670      1680
           ....|....|....|....|....|....|....|....|....|....|....|....|....|....|....|....|
KU70cDNA   ~~~~~~~~~~~~~~~~~~~~~~~~~~~~~~~~~~~~~~~~~~~~~~~~~~~~~~~~~~~~~~~~~~~~~~~~~~~~~~~~ 428
KU70-A     TGCTACAAGACTGAAACTCAGACATTGTATTATCTCAACTGATTCTGGCATTTTACATCTCATGTTGATTTCATCTTTTG 1680
HY1 cDNA   ~~~~~~~~~~~~~~~~~~~~~~~~~~~~~~~~~~~~~~~~~~~~~~~~~~~~~~~~~~~~~~~~~~~~~~~~~~~~~~~~ 376

                1690      1700      1710      1720      1730      1740      1750      1760
           ....|....|....|....|....|....|....|....|....|....|....|....|....|....|....|....|
KU70cDNA   ~~~~~~~~~~~~~~~~~~~~~~~~~~~~~~~~~~~~~~~~~~~~~~~~~~~~~~~~~~~~~~~~~~~~~~~~~~~~~~~~ 428
KU70-A     TGTTCTCTTGGTCACTAAAAAATTGTAAGGCAACCATGCTTTTAGAAAGTTAGCTAGGAAATATCATTAGTAGCAGTACA 1760
HY1 cDNA   ~~~~~~~~~~~~~~~~~~~~~~~~~~~~~~~~~~~~~~~~~~~~~~~~~~~~~~~~~~~~~~~~~~~~~~~~~~~~~~~~ 376

                1770      1780      1790      1800      1810      1820      1830      1840
           ....|....|....|....|....|....|....|....|....|....|....|....|....|....|....|....|
KU70cDNA   ~~~~~~~~~~~~~~~~~~~~~~~~~~~~~~~~~~~~~~~~~~~~~~~~~~~~~~~~~~~~~~~~~~~~~~~~~~~~~~~~ 428
KU70-A     TGAACATTGTAAGCATGATTTTTATTTCATTTTGTTTTGGCCTGAATAGAAAGGCTCTATGGATAAAATTGGAGTAGGGT 1840
HY1 cDNA   ~~~~~~~~~~~~~~~~~~~~~~~~~~~~~~~~~~~~~~~~~~~~~~~~~~~~~~~~~~~~~~~~~~~~~~~~~~~~~~~~ 376

                1850      1860      1870      1880      1890      1900      1910      1920
           ....|....|....|....|....|....|....|....|....|....|....|....|....|....|....|....|
KU70cDNA   ~~~~~~~~~~~~~~~~~~~~~~~~~~~~~~~~~~~~~~~~~~~~~~~~~~~~~~~~~~~~~~~~ATTCTTTTATGAATAC 444
KU70-A     ACTTGTGTATGAGAGAACTCAGCAATTATGTTTTCATTATAATCTTTGTTTTCACTTGTGCAAG...............A 1920
HY1 cDNA   ~~~~~~~~~~~~~~~~~~~~~~~~~~~~~~~~~~~~~~~~~~~~~~~~~~~~~~~~~~~~~~~~...............A 392

                1930      1940      1950      1960      1970      1980      1990      2000
           ....|....|....|....|....|....|....|....|....|....|....|....|....|....|....|....|
KU70cDNA   CATTGGAAGCCGGTATGGAATAACCTCTGGATCTAGAGAGAATACCCTGTACAATGCTCTTTGGGTTGCACAGGCACTGC 524
KU70-A     ..................................C............................................. 2000
HY1 cDNA   ..................................C............................................. 472

                2010      2020      2030      2040      2050      2060      2070      2080
           ....|....|....|....|....|....|....|....|....|....|....|....|....|....|....|....|
KU70cDNA   TGCGTAAAGG~~~~~~~~~~~~~~~~~~~~~~~~~~~~~~~~~~~~~~~~~~~~~~~~~~~~~~~~~~~~~~~~~~~~~~ 534
KU70-A     ..........GTAACTTCACTTTTCGCCTTTGTTTCTCAGATGCCGTAGTTTATATTAATTAACACATCCCAGTAGCATT 2080
```

```
HY1 cDNA  ..........~~~~~~~~~~~~~~~~~~~~~~~~~~~~~~~~~~~~~~~~~~~~~~~~~~~~~~~~~~~~~~~~ 482

                   2090      2100      2110      2120      2130      2140      2150      2160
          ....|....|....|....|....|....|....|....|....|....|....|....|....|....|....|....|
KU70cDNA  ~~~~~~~~~~~~~~~~~~~~~~~~~~~~~~~~~~~~~~~~~~~~~~~~~~~~~~~~~~~~~~~~~~~~~~~~~~~~~~~~ 534
KU70-A    AAACATCTTTGTAGAGGTGTAGCCTGTATACATGACAAGTATTGTCTATAACTTTCTTGGGGGATACAGTTCAGCAGTAA 2160
HY1 cDNA  ~~~~~~~~~~~~~~~~~~~~~~~~~~~~~~~~~~~~~~~~~~~~~~~~~~~~~~~~~~~~~~~~~~~~~~~~~~~~~~~~ 482

                   2170      2180      2190      2200      2210      2220      2230      2240
          ....|....|....|....|....|....|....|....|....|....|....|....|....|....|....|....|
KU70cDNA  ~~~~~~~~~~~~~~~~~~~~~~~~~~~~~~~~~~~~~~~~~~~~~~~~~~~~~~~~~~~~~~~~~~~~~~~~~~~~~~~~ 534
KU70-A    AGCTTAAATTTCTATATATTATGCACAACGATCTAGTTTGCATCAGCTAGATCATTGTGCACATTATACTTATTAGCTAT 2240
HY1 cDNA  ~~~~~~~~~~~~~~~~~~~~~~~~~~~~~~~~~~~~~~~~~~~~~~~~~~~~~~~~~~~~~~~~~~~~~~~~~~~~~~~~ 482

                   2250      2260      2270      2280      2290      2300      2310      2320
          ....|....|....|....|....|....|....|....|....|....|....|....|....|....|....|....|
KU70cDNA  ~~~~~~~~~~~~~~~~~~~~~~~~~~~~~~~~~~~~~~~~~~~~~~~~~~~~~~~~~~~~~~~~~~~~~~~~~~~~~~~~ 534
KU70-A    AAGTGAATGTAATATACCTTAGGTGCTTTAACAGTGTTTTGCTGGATGTTGAAGCATAAATTTACGTGTTAGTGAATGCA 2320
HY1 cDNA  ~~~~~~~~~~~~~~~~~~~~~~~~~~~~~~~~~~~~~~~~~~~~~~~~~~~~~~~~~~~~~~~~~~~~~~~~~~~~~~~~ 482

                   2330      2340      2350      2360      2370      2380      2390      2400
          ....|....|....|....|....|....|....|....|....|....|....|....|....|....|....|....|
KU70cDNA  ~~~~~~~~~~~~~~~~~~~~~~~~~~~~~~~~~~~~~~~~~~~~~~~~~~~~~~~~~~~~~~~~~~~~~~~~~~~~~~~~ 534
KU70-A    GCATCCTTTAGGTGCTTTAGCTATCAACCTATCATCTAATGTAATGGTTAACACCTATGTGGTTCAACTTCAGTGATTCC 2400
HY1 cDNA  ~~~~~~~~~~~~~~~~~~~~~~~~~~~~~~~~~~~~~~~~~~~~~~~~~~~~~~~~~~~~~~~~~~~~~~~~~~~~~~~~ 482

                   2410      2420      2430      2440      2450      2460      2470      2480
          ....|....|....|....|....|....|....|....|....|....|....|....|....|....|....|....|
KU70cDNA  ~~~~~~~~~~~~~~~~~~~~~~~~~ATCTGTGAAGACTGTGAGTAAGAGAATCCTCATATTCACCAATGAGGATGATCCT 589
KU70-A    CCTTTCTTCTTTTTTCATTCCTCAG....................................................... 2480
HY1 cDNA  ~~~~~~~~~~~~~~~~~~~~~~~~~....................................................... 537

                   2490      2500      2510      2520      2530      2540      2550      2560
          ....|....|....|....|....|....|....|....|....|....|....|....|....|....|....|....|
KU70cDNA  TTTGGTGGTATTACAGGAGCAGCAAAGACTGATATGATTAGGACCACAATTCAACGTGCAAAA~~~~~~~~~~~~~~~~~ 652
KU70-A    .......................................C.......................GTTTGTCTATGACACAT 2560
HY1 cDNA  .......................................C.......................GTTTGTCTATGACACAT 617

                   2570      2580      2590      2600      2610      2620      2630      2640
          ....|....|....|....|....|....|....|....|....|....|....|....|....|....|....|....|
KU70cDNA  ~~~~~~~~~~~~~~~~~~~~~~~~~~~~~~~~~~~~~~~~~~~~~~~~~~~~~~~~~~~~~~~~~~~~~~~~~~~~~~~~ 652
KU70-A    ACATCTATTCTGTTTTCACACCAAGCAGCCCACAGATGCAAATTAATATGGTATGTTCCTAATGATCTTATGTTTTTGTT 2640
HY1 cDNA  ACATCTATTCTGTTTTCACACCAAGCAGCCCACAGATGCAAATTAATATG~~~~~~~~~~~~~~~~~~~~~~~~~~~~~~ 667

                   2650      2660      2670      2680      2690      2700      2710      2720
          ....|....|....|....|....|....|....|....|....|....|....|....|....|....|....|....|
KU70cDNA  ~~~~~~~~~~~~~~~~~~~~~~~~GATGCACAAGATCTGGGCCTGTCTATCGAACTTCTTCCATTGAGTAGGCCTGATGA 708
KU70-A    CTATAATTACTTGCCTACCTTCAG.........................T..............A............... 2720
HY1 cDNA  ~~~~~~~~~~~~~~~~~~~~~~~~~~~~~~~~~~~~~~~~~~~~~~~~~~~~~~~~~~~~~~~~~~~~~~~~~~~~~~~~ 667

                   2730      2740      2750      2760      2770      2780      2790      2800
          ....|....|....|....|....|....|....|....|....|....|....|....|....|....|....|....|
KU70cDNA  GGATTTCAACATGTCCCTGTTTTATGCA~~~~~~~~~~~~~~~~~~~~~~~~~~~~~~~~~~~~~~~~~~~~~~~~~~~~ 736
KU70-A    ............................GTAAGTCTCCGCAGCTCCCCTCTCCCCCCGTATCAGTGCCAAATGCTAAGCA 2800
HY1 cDNA  ~~~~~~~~~~~~~~~~~~~~~~~~~~~~~TAAGTCTCCGCAGCTCCCCTCTCCCCCCGTATCAGTGCCAAATGCTAAGCA 718

                   2810      2820      2830      2840      2850      2860      2870      2880
          ....|....|....|....|....|....|....|....|....|....|....|....|....|....|....|....|
KU70cDNA  ~~~~~~~~~~~~~~~GATTTGATTGGTCTGGAGGGAGATGAAGTACTGCAGTATGTGCCATCTGCTGGTGAAAA~~~~~~ 795
KU70-A    ACTTCCATCTCCCAG..............................G.......C....................GTAATT 2880
HY1 cDNA  ACTTCCATCTCCCAG..............................G.......C....................~~~~~~ 792

                   2890      2900      2910      2920      2930      2940      2950      2960
          ....|....|....|....|....|....|....|....|....|....|....|....|....|....|....|....|
KU70cDNA  ~~~~~~~~~~~~~~~~~~~~~~~~~~~~~~~~~~~~~~~~~~~~~~~~~~~~~~~~~~~~~~~~~~~~~~~~~~~~~~~~ 795
KU70-A    TCTTTCTTTAATCACCAATTTTACTCTTGATTGTTTCTTGTCTTGTTGATACAAATGTAAATACTCAACGGTATCTTATT 2960
HY1 cDNA  ~~~~~~~~~~~~~~~~~~~~~~~~~~~~~~~~~~~~~~~~~~~~~~~~~~~~~~~~~~~~~~~~~~~~~~~~~~~~~~~~ 792

                   2970      2980      2990      3000      3010      3020      3030      3040
          ....|....|....|....|....|....|....|....|....|....|....|....|....|....|....|....|
KU70cDNA  ~~~~~~~~~~~~~~~~~~~~~~~~~~~~~~~~~~~~~~~~~~~~~~~~~~~~~~~~~~~~~~~~~~~~~~~~~~~~~~~~ 795
KU70-A    CTTCTAATTACTTTCCGCAAAGAAAAGAAAAAAGACAAATATTGGTGCTCGAGTGGGAGCTTGCAACTTTGTTGAATGGA 3040
HY1 cDNA  ~~~~~~~~~~~~~~~~~~~~~~~~~~~~~~~~~~~~~~~~~~~~~~~~~~~~~~~~~~~~~~~~~~~~~~~~~~~~~~~~ 792

                   3050      3060      3070      3080      3090      3100      3110      3120
          ....|....|....|....|....|....|....|....|....|....|....|....|....|....|....|....|
KU70cDNA  ~~~~~~~~~~~~~~~~~~~~~~~~~~~~~~~~~~~~~~~~~~~~~~~~~~~~~~~~~~~~~~~~~~~~~~~~~~~~~~~~ 795
```

```
KU70-A     GATAATCTGTGTCTCAGAACCAGATCTGAGAACAAATATACAGTCTGTTGTTTCTTGTGACTATGTAGACTTGCTATTGT 3120
HY1 cDNA   ~~~~~~~~~~~~~~~~~~~~~~~~~~~~~~~~~~~~~~~~~~~~~~~~~~~~~~~~~~~~~~~~~~~~~~~~~~~~~~~~ 792

                    3130      3140      3150      3160      3170      3180      3190      3200
           ....|....|....|....|....|....|....|....|....|....|....|....|....|....|....|....|
KU70cDNA   ~~~~~~~~~~~~~~~~~~~~~~~~~~~~~~~~~~~~~~~~~~~~~~~~~~~~~~~~~~~~~~~~~~~~~~~~~~~~~~~~ 795
KU70-A     CCTCCCACGAATTTTGTGCGTAGTGGGTGTCAACTCTGTGCATTGTCCTCCACATGTTATAAGAACATAGGATAAAACAT 3200
HY1 cDNA   ~~~~~~~~~~~~~~~~~~~~~~~~~~~~~~~~~~~~~~~~~~~~~~~~~~~~~~~~~~~~~~~~~~~~~~~~~~~~~~~~ 792

                    3210      3220      3230      3240      3250      3260      3270      3280
           ....|....|....|....|....|....|....|....|....|....|....|....|....|....|....|....|
KU70cDNA   ~~~~~~~~~~~~~~~~~~~~~~~~~~~~~~~~~~~~~~~~~~~~~~~~~~~~~~~~~~~~~~~~~~~~~~~~~~~~~~~~ 795
KU70-A     CTGAAGTAATGGTTAACTTGTTATCTTTGTTCTCGCCAAAGAAAACTTGTTAATCTTTGTTTAAGCGTACAAATGCTTCC 3280
HY1 cDNA   ~~~~~~~~~~~~~~~~~~~~~~~~~~~~~~~~~~~~~~~~~~~~~~~~~~~~~~~~~~~~~~~~~~~~~~~~~~~~~~~~ 792

                    3290      3300      3310      3320      3330      3340      3350      3360
           ....|....|....|....|....|....|....|....|....|....|....|....|....|....|....|....|
KU70cDNA   ~~~~~~~~~~~~~~~~~~~~~~~~~~~~~~~~~~~~~~~~~~~~~~~~~~~~~~~~~~~~~~~~~~~~~~~~~~~~~~~~ 795
KU70-A     ATAAGGTTTTTTGCTGTTCTGCATGAAATTTAGGTTAAGTAACCTCAGGTGCACTTCTTGTGTGAACTAATCCAGCTGTA 3360
HY1 cDNA   ~~~~~~~~~~~~~~~~~~~~~~~~~~~~~~~~~~~~~~~~~~~~~~~~~~~~~~~~~~~~~~~~~~~~~~~~~~~~~~~~ 792

                    3370      3380      3390      3400      3410      3420      3430      3440
           ....|....|....|....|....|....|....|....|....|....|....|....|....|....|....|....|
KU70cDNA   ~~~~~~~~~~~~~~~~~~~~~~~~~~~~~~~~~~~~~~~~~~~~~~~~~~~~~~~~~~~~~~~~~~~~~~~~~~~~~~~~ 795
KU70-A     AAACCATTATGTGCCCGGGTGTAGGGAAGAGGTGGATGCATATGTAGGCGAGAATTGTGGTTTGAGCACAAGGAAGAGCG 3440
HY1 cDNA   ~~~~~~~~~~~~~~~~~~~~~~~~~~~~~~~~~~~~~~~~~~~~~~~~~~~~~~~~~~~~~~~~~~~~~~~~~~~~~~~~ 792

                    3450      3460      3470      3480      3490      3500      3510      3520
           ....|....|....|....|....|....|....|....|....|....|....|....|....|....|....|....|
KU70cDNA   ~~~~~~~~~~~~~~~~~~~~~~~~~~~~~~~~~~~~~~~~~~~~~~~~~~~~~~~~~~~~~~~~~~~~~~~~~~~~~~~~ 795
KU70-A     GTTCACTCTTGTACACGCGGCTCTTGTACACGCGGCATGGCTCTTCCCCAGCCGCGGAGCAGGGCTCTGAACACTTGTGA 3520
HY1 cDNA   ~~~~~~~~~~~~~~~~~~~~~~~~~~~~~~~~~~~~~~~~~~~~~~~~~~~~~~~~~~~~~~~~~~~~~~~~~~~~~~~~ 792

                    3530      3540      3550      3560      3570      3580      3590      3600
           ....|....|....|....|....|....|....|....|....|....|....|....|....|....|....|....|
KU70cDNA   ~~~~~~~~~~~~~~~~~~~~~~~~~~~~~~~~~~~~~~~~~~~~~~~~~~~~~~~~~~~~~~~~~~~~~~~~~~~~~~~~ 795
KU70-A     CTTGGAGAATCTATTTGTTGCTTAATTCAACTAGTTATACAGTTCACTTACATAGCAGACCCTAGAGAAAGGATACCATA 3600
HY1 cDNA   ~~~~~~~~~~~~~~~~~~~~~~~~~~~~~~~~~~~~~~~~~~~~~~~~~~~~~~~~~~~~~~~~~~~~~~~~~~~~~~~~ 792

                    3610      3620      3630      3640      3650      3660      3670      3680
           ....|....|....|....|....|....|....|....|....|....|....|....|....|....|....|....|
KU70cDNA   ~~~~~~~~~~~~~~~~~~~~~~~~~~~~~~~~~~~~~~~~~~~~~~~~~~~~~~~~~~~~~~~~~~~~~~~~~~~~~~~~ 795
KU70-A     CACAATTGGCATATAGCCAACTAGTAATATTCTAATATGTAGCTAACTGCTACTAGGACACAAACTCTTCTGGGCTGCAA 3680
HY1 cDNA   ~~~~~~~~~~~~~~~~~~~~~~~~~~~~~~~~~~~~~~~~~~~~~~~~~~~~~~~~~~~~~~~~~~~~~~~~~~~~~~~~ 792

                    3690      3700      3710      3720      3730      3740      3750      3760
           ....|....|....|....|....|....|....|....|....|....|....|....|....|....|....|....|
KU70cDNA   ~~~~~~~~~~~~~~~~~~~~~~~~~~~~~~~~~~~~~~~~~~~~~~~~~~~~~~~~~~~~~~~~~~~~~~~~~~~~~~~~ 795
KU70-A     TCAGGCGCCTCTCCTGATTGGGTCATTGAAACACGGACTAGTAGTCCTAGTCGATTATCACCTTACCGTCCAAAGTGGGT 3760
HY1 cDNA   ~~~~~~~~~~~~~~~~~~~~~~~~~~~~~~~~~~~~~~~~~~~~~~~~~~~~~~~~~~~~~~~~~~~~~~~~~~~~~~~~ 792

                    3770      3780      3790      3800      3810      3820      3830      3840
           ....|....|....|....|....|....|....|....|....|....|....|....|....|....|....|....|
KU70cDNA   ~~~~~~~~~~~~~~~~~~~~~~~~~~~~~~~~~~~~~~~~~~~~~~~~~~~~~~~~~~~~~~~~~~~~~~~~~~~~~~~~ 795
KU70-A     TCAGATGCTGCTGCTGTTGCTGTCTTGGATCTGCGCCTGTAGGTCTGCTGCGCCCGTAACAAAAACTTGAATGGATTTTT 3840
HY1 cDNA   ~~~~~~~~~~~~~~~~~~~~~~~~~~~~~~~~~~~~~~~~~~~~~~~~~~~~~~~~~~~~~~~~~~~~~~~~~~~~~~~~ 792

                    3850      3860      3870      3880      3890      3900      3910      3920
           ....|....|....|....|....|....|....|....|....|....|....|....|....|....|....|....|
KU70cDNA   ~~~~~~~~~~~~~~~~~~~~~~~~~~~~~~~~~~~~~~~~~~~~~~~~~~~~~~~~~~~~~~~~~~~~~~~~~~~~~~~~ 795
KU70-A     TGCTTGATCATCAACAAGAATCGTTAGCTAACTTTGTAGAGCTTTCAGAAGCTCTAGCTACTCCCTCCGTCCGGAAATAC 3920
HY1 cDNA   ~~~~~~~~~~~~~~~~~~~~~~~~~~~~~~~~~~~~~~~~~~~~~~~~~~~~~~~~~~~~~~~~~~~~~~~~~~~~~~~~ 792

                    3930      3940      3950      3960      3970      3980      3990      4000
           ....|....|....|....|....|....|....|....|....|....|....|....|....|....|....|....|
KU70cDNA   ~~~~~~~~~~~~~~~~~~~~~~~~~~~~~~~~~~~~~~~~~~~~~~~~~~~~~~~~~~~~~~~~~~~~~~~~~~~~~~~~ 795
KU70-A     TTGTCATCAAAATGGATAAAAGGAGATGTATCTAGATGTATTTTAGTTCTAGATACATCCCTTTTTGTCCATTTTGATGA 4000
HY1 cDNA   ~~~~~~~~~~~~~~~~~~~~~~~~~~~~~~~~~~~~~~~~~~~~~~~~~~~~~~~~~~~~~~~~~~~~~~~~~~~~~~~~ 792

                    4010      4020      4030      4040      4050      4060      4070      4080
           ....|....|....|....|....|....|....|....|....|....|....|....|....|....|....|....|
KU70cDNA   ~~~~~~~~~~~~~~~~~~~~~~~~~~~~~~~~~~~~~~~~~~~~~~~~~~~~~~~~~~~~~~~~~~~~~~~~~~~~~~~~ 795
KU70-A     CAAGTATTTTCGGACGGAGGGAGTATATCTTAGCATCTCTTGGTATGAATGATAATTTTTATGCTACCAGTCTGTTACCC 4080
HY1 cDNA   ~~~~~~~~~~~~~~~~~~~~~~~~~~~~~~~~~~~~~~~~~~~~~~~~~~~~~~~~~~~~~~~~~~~~~~~~~~~~~~~~ 792

                    4090      4100      4110      4120      4130      4140      4150      4160
           ....|....|....|....|....|....|....|....|....|....|....|....|....|....|....|....|
```

```
KU70cDNA  ~~~~~~~~~~~~~~~~~~~~~~~~~~~~~~~~~~~~~~~~~~~~~~~~~~~~~~~~~~~~~~~~~~~~~~~~~~~~~~~~ 795
KU70-A    TAATCATTATGGAAGGTTATGCTTATGCTTTACTTTAGTTAGATAGTTTGGTTTAGACCCACTTTGAGCCTCATTCTAGT 4160
HY1 cDNA  ~~~~~~~~~~~~~~~~~~~~~~~~~~~~~~~~~~~~~~~~~~~~~~~~~~~~~~~~~~~~~~~~~~~~~~~~~~~~~~~~ 792

                  4170      4180      4190      4200      4210      4220      4230      4240
          ....|....|....|....|....|....|....|....|....|....|....|....|....|....|....|....|
KU70cDNA  ~~~~~~~~~~~~~~GCTGGAGGATATGACTGATCAACTGAGAAAGCGAATGATGAAAAAGCGCAAAGTCAAAACTCTCTC 861
KU70-A    GTTGTTCTTTGAAG.................................................................. 4240
HY1 cDNA  ~~~~~~~~~~~~~~.................................................................. 858

                  4250      4260      4270      4280      4290      4300      4310      4320
          ....|....|....|....|....|....|....|....|....|....|....|....|....|....|....|....|
KU70cDNA  ATTTGCAATTACAAATGATGTTTGCATAGAGGTGAACACATATGCGTTAATCCGTCCAACTGCTCCAG~~~~~~~~~~~~ 929
KU70-A    ............G.................................C.....................GTATGCTCTGAC 4320
HY1 cDNA  ............G.................................C.....................~~~~~~~~~~~~ 926

                  4330      4340      4350      4360      4370      4380      4390      4400
          ....|....|....|....|....|....|....|....|....|....|....|....|....|....|....|....|
KU70cDNA  ~~~~~~~~~~~~~~~~~~~~~~~~~~~~~~~~~~~~~~~~~~~~~~~~~~~~~~~~~~~~~~~~~~~~~~GGACGATCAC 939
KU70-A    TCTGTCTTTCCAATGCTTGTTTACAAGTTGTTGTAACTTAAGCGTAAATGTGTTACTTCTATATTTGTAG.......T.. 4400
HY1 cDNA  ~~~~~~~~~~~~~~~~~~~~~~~~~~~~~~~~~~~~~~~~~~~~~~~~~~~~~~~~~~~~~~~~~~~~~~.......T.. 936

                  4410      4420      4430      4440      4450      4460      4470      4480
          ....|....|....|....|....|....|....|....|....|....|....|....|....|....|....|....|
KU70cDNA  GTGGCTTGACTCGATCAGTAACCTTCCATTAAAG~~~~~~~~~~~~~~~~~~~~~~~~~~~~~~~~~~~~~~~~~~~~~~ 973
KU70-A    ..................................GTAAAGTTATGCTCTATGAAAAATATATGCTCTTTGTTGCTTACTT 4480
HY1 cDNA  ..................................~~~~~~~~~~~~~~~~~~~~~~~~~~~~~~~~~~~~~~~~~~~~~~ 970

                  4490      4500      4510      4520      4530      4540      4550      4560
          ....|....|....|....|....|....|....|....|....|....|....|....|....|....|....|....|
KU70cDNA  ~~~~~~~~~~~~~~~~~~~~~~~~~~~~~~~~~~~~~~~~~~~~~~~~~~~~~~~~~~~~~~~~~~~~~~~~~~~~~~~~ 973
KU70-A    TTTTGGATTTTGCTGATGCACCTTCATAGTTTGTTATGCTATCATTACAAAAGTTGTGACGAGCTTATGGTAGGCTTAAG 4560
HY1 cDNA  ~~~~~~~~~~~~~~~~~~~~~~~~~~~~~~~~~~~~~~~~~~~~~~~~~~~~~~~~~~~~~~~~~~~~~~~~~~~~~~~~ 970

                  4570      4580      4590      4600      4610      4620      4630      4640
          ....|....|....|....|....|....|....|....|....|....|....|....|....|....|....|....|
KU70cDNA  ~~~~~~~~~~~~~~~~~~~~~~~~~~~~~~~~~~~~~~~~~~~~~~~~~~~~~~~~~~~~~~~~~~~~~~~~~~~~~~~~ 973
KU70-A    TCATACTCCCTCTGTATCACAAATACATGTCGTTTCGGATATTGACATGGTCTTCAAGATTTACCTTTGACCACTAATTT 4640
HY1 cDNA  ~~~~~~~~~~~~~~~~~~~~~~~~~~~~~~~~~~~~~~~~~~~~~~~~~~~~~~~~~~~~~~~~~~~~~~~~~~~~~~~~ 970

                  4650      4660      4670      4680      4690      4700      4710      4720
          ....|....|....|....|....|....|....|....|....|....|....|....|....|....|....|....|
KU70cDNA  ~~~~~~~~~~~~~~~~~~~~~~~~~~~~~~~~~~~~~~~~~~~~~~~~~~~~~~~~~~~~~~~~~~~~~~~~~~~~~~~~ 973
KU70-A    TACTATTGTAATAGTATGCATACATAACACAAGAAAATTATCATAGTATGAGTACTTCTCAAAACAAATATAAGCATATG 4720
HY1 cDNA  ~~~~~~~~~~~~~~~~~~~~~~~~~~~~~~~~~~~~~~~~~~~~~~~~~~~~~~~~~~~~~~~~~~~~~~~~~~~~~~~~ 970

                  4730      4740      4750      4760      4770      4780      4790      4800
          ....|....|....|....|....|....|....|....|....|....|....|....|....|....|....|....|
KU70cDNA  ~~~~~~~~~~~~~~~~~~~~~~~~~~~~~~~~~~~~~~~~~~~~~~~~~~~~~~~~~~~~~~~~~~~~~~~~~~~~~~~~ 973
KU70-A    GTTTGCAAGTTCTCAAACTAAATATTTGAAACAATATTTATAGTCGAAGTTTGAAAATTTTGACTGAAGGCATTTCCAGA 4800
HY1 cDNA  ~~~~~~~~~~~~~~~~~~~~~~~~~~~~~~~~~~~~~~~~~~~~~~~~~~~~~~~~~~~~~~~~~~~~~~~~~~~~~~~~ 970

                  4810      4820      4830      4840      4850      4860      4870      4880
          ....|....|....|....|....|....|....|....|....|....|....|....|....|....|....|....|
KU70cDNA  ~~~~~~~~~~~~~~~~~~~~~~~~~~~~~~~~~~~~~~~~~~~~~~~~~~~~~~~~~~~~~~~~~~~~~~~~~~~~~~~~ 973
KU70-A    ACAAATATTTGTAATATGGAGGGTAGCTAAGAATTGTGTAGATAACATCCTGGCTATGCAGTACCATCTCTTGGATAATG 4880
HY1 cDNA  ~~~~~~~~~~~~~~~~~~~~~~~~~~~~~~~~~~~~~~~~~~~~~~~~~~~~~~~~~~~~~~~~~~~~~~~~~~~~~~~~ 970

                  4890      4900      4910      4920      4930      4940      4950      4960
          ....|....|....|....|....|....|....|....|....|....|....|....|....|....|....|....|
KU70cDNA  ~~~~~~~~~~~~~~~~~~~~~~~~~~~~~~~~~~~~~~~~~~~~~~~~~~~~~~~~~~~~~~~~~~~~~~~~~~~~~~~~ 973
KU70-A    TTGAAAGAGCATCTAAGCAATTCTTTTCCTGCTCTAATTTTTAAGGATATTTGGTTAATTTGATTGGGAAGTGAAAATGT 4960
HY1 cDNA  ~~~~~~~~~~~~~~~~~~~~~~~~~~~~~~~~~~~~~~~~~~~~~~~~~~~~~~~~~~~~~~~~~~~~~~~~~~~~~~~~ 970

                  4970      4980      4990      5000      5010      5020      5030      5040
          ....|....|....|....|....|....|....|....|....|....|....|....|....|....|....|....|
KU70cDNA  ~~~~~~~~~~~~~~~~~~~~~~~~~~~~~~~~~~~~~~~~~~~~~~~~~~~~~~~~~~~~~~~~~~~~~~~~~~~~~~~~ 973
KU70-A    TTTCATGGTTGATGATGCAACATTCTATAGTCTGCCAGTGTGGTAACTGATTTATTGTAGACCCAGATTACGCAACGCCA 5040
HY1 cDNA  ~~~~~~~~~~~~~~~~~~~~~~~~~~~~~~~~~~~~~~~~~~~~~~~~~~~~~~~~~~~~~~~~~~~~~~~~~~~~~~~~ 970

                  5050      5060      5070      5080      5090      5100      5110      5120
          ....|....|....|....|....|....|....|....|....|....|....|....|....|....|....|....|
KU70cDNA  ~~~~~~~~~~~~~~~~~~~~~~~~~~~~~~~~~~~~~~~~~~~~~~~~~~~~~~~~~~~~~~~~~~~~~~~~~~~~~~~~ 973
KU70-A    ATCTTTTACCATGATAGGGCAAAAGATTGACGCAACCTGTGTATAGGATCAGTACCCAGCACTTCCACATGTTTTGAGTA 5120
HY1 cDNA  ~~~~~~~~~~~~~~~~~~~~~~~~~~~~~~~~~~~~~~~~~~~~~~~~~~~~~~~~~~~~~~~~~~~~~~~~~~~~~~~~ 970

                  5130      5140      5150      5160      5170      5180      5190      5200
```

```
          ....|....|....|....|....|....|....|....|....|....|....|....|....|....|....|....|
KU70cDNA  ~~~~~~~~~~~~~~~~~~~~~~~~~~~~~~~~~~~~~~~~~~~~~~~~~~~~~~~~~~~~~~~~~~~~~~~~~~~~~~~~ 973
KU70-A    TGCATTTACGTAAGCAGCTATTACTGTCTTGATCATAGAGCTACTATTTTATGGTCATGCATATCTTGACCTTCAAGGGA 5200
HY1 cDNA  ~~~~~~~~~~~~~~~~~~~~~~~~~~~~~~~~~~~~~~~~~~~~~~~~~~~~~~~~~~~~~~~~~~~~~~~~~~~~~~~~ 970

                5210      5220      5230      5240      5250      5260      5270      5280
          ....|....|....|....|....|....|....|....|....|....|....|....|....|....|....|....|
KU70cDNA  ~~~~~~~~~~~~~~~~~~~~~~~~~~~~~~~~~~~~~~~~~~~~~~~~~~~~~~~~~~~~~~~~~~~~~~~~~~~~~~~~ 973
KU70-A    GTTAAGGTTGAGATGCCATGTGTCTGCCACACAAAAAGGAAACGGATCAAGGACTAGTCACTGTGTAGAGGAGTACTATT 5280
HY1 cDNA  ~~~~~~~~~~~~~~~~~~~~~~~~~~~~~~~~~~~~~~~~~~~~~~~~~~~~~~~~~~~~~~~~~~~~~~~~~~~~~~~~ 970

                5290      5300      5310      5320      5330      5340      5350      5360
          ....|....|....|....|....|....|....|....|....|....|....|....|....|....|....|....|
KU70cDNA  ~~~~~~~~~~~~~~~~~~~~~~~~~~~~~~~~~~~~~~~~~~~~~~~~~~~~~~~~~~~~~~~~~~~~~~~~~~~~~~~~ 973
KU70-A    ATAGTGCCTCTTGGGCATAACAGAACATTCAACACTTTCACATATTGTACACAGTCTTTGGGCTTTTTTTTTTCTTCGGA 5360
HY1 cDNA  ~~~~~~~~~~~~~~~~~~~~~~~~~~~~~~~~~~~~~~~~~~~~~~~~~~~~~~~~~~~~~~~~~~~~~~~~~~~~~~~~ 970

                5370      5380      5390      5400      5410      5420      5430      5440
          ....|....|....|....|....|....|....|....|....|....|....|....|....|....|....|....|
KU70cDNA  ~~~~~~~~~~~~~~~~~~~~~~~~~~~~~~~~~~~~~~~~~~~~~~~~~~~~~~~~~~~~~~~~~~~~~~~~~~~~~~~~ 973
KU70-A    ATCTGTTTCTGAATGTACTCACATAATGAGATGTTGCATATCTTATGGAAGTGAGAGTTTTTGTTGTCATTAACTTCCTT 5440
HY1 cDNA  ~~~~~~~~~~~~~~~~~~~~~~~~~~~~~~~~~~~~~~~~~~~~~~~~~~~~~~~~~~~~~~~~~~~~~~~~~~~~~~~~ 970

                5450      5460      5470      5480      5490      5500      5510      5520
          ....|....|....|....|....|....|....|....|....|....|....|....|....|....|....|....|
KU70cDNA  ~~~~~~~~~~~~~~~~~~~~~~~~~~~~~~~~~~~~~~~~~~~~~~~~~~~~~~~~~~~~~~~~~~~~~~~~~~~~~~~~ 973
KU70-A    TTATTAGGGAAATTTTACAGTGCAACTCTTCTCTTACAGTTGCTTTTTGCTTACATTTCCTTAACACATAAAATGATGTT 5520
HY1 cDNA  ~~~~~~~~~~~~~~~~~~~~~~~~~~~~~~~~~~~~~~~~~~~~~~~~~~~~~~~~~~~~~~~~~~~~~~~~~~~~~~~~ 970

                5530      5540      5550      5560      5570      5580      5590      5600
          ....|....|....|....|....|....|....|....|....|....|....|....|....|....|....|....|
KU70cDNA  ~~~~~~~ACTGAAAGGTCATTCATATGCAATGACACCGGGGCCCTTCTTCAGGCTCCCCAAGAGCGCTTCCAGCTATACA 1046
KU70-A    TATACAG...........T............................................................. 5600
HY1 cDNA  ~~~~~~~...........T............................................................. 1043

                5610      5620      5630      5640      5650      5660      5670      5680
          ....|....|....|....|....|....|....|....|....|....|....|....|....|....|....|....|
KU70cDNA  ATGA~~~~~~~~~~~~~~~~~~~~~~~~~~~~~~~~~~~~~~~~~~~~~~~~~~~~~~~~~~~~~~~~~~~~~~~~~~~~ 1050
KU70-A    ....GTAAGTACACATATGTTTATACAGTGTCATCTAATAATGTATTTTTTTGGTTTCTAGATTTATTTGTGTTATACTT 5680
HY1 cDNA  ....~~~~~~~~~~~~~~~~~~~~~~~~~~~~~~~~~~~~~~~~~~~~~~~~~~~~~~~~~~~~~~~~~~~~~~~~~~~~ 1047

                5690      5700      5710      5720      5730      5740      5750      5760
          ....|....|....|....|....|....|....|....|....|....|....|....|....|....|....|....|
KU70cDNA  ~~~~~~~~~~~~~~~~~~~~~~~~~~~~~~~~~~~~~~~~~~~~~~~~~~~~~~~~~~~~~~~~~~~~~~~~~~~~~~~~ 1050
KU70-A    CCAGTATCTGCTTCCTGATTTCTTCACCAATCTTTCCATTATTTGTTTTCTTGTTTCAGTTTGATGCTTATATTTGTGAT 5760
HY1 cDNA  ~~~~~~~~~~~~~~~~~~~~~~~~~~~~~~~~~~~~~~~~~~~~~~~~~~~~~~~~~~~~~~~~~~~~~~~~~~~~~~~~ 1047

                5770      5780      5790      5800      5810      5820      5830      5840
          ....|....|....|....|....|....|....|....|....|....|....|....|....|....|....|....|
KU70cDNA  ~~~~~~~~~~~~~~~~~~~~~~~~~~~~~~~~~~TAAAGTTGTTAAATTCTCTGTTCGTGAATTGTCTGATGTGAAGAGG 1096
KU70-A    CTTAAAGCACTTTTATGTATTCATGTTTGTGCAG...............T.............................. 5840
HY1 cDNA  ~~~~~~~~~~~~~~~~~~~~~~~~~~~~~~~~~~...............T.............................. 1093

                5850      5860      5870      5880      5890      5900      5910      5920
          ....|....|....|....|....|....|....|....|....|....|....|....|....|....|....|....|
KU70cDNA  GTTTCAAGTCATCATCTTCGCCTTTTAGGGTTCAAGCCATTGGATTGCTTAAAAGATTATCATAACTTAAGTCCATCAAC 1176
KU70-A    ..........................................................................G..... 5920
HY1 cDNA  ..............C................................................................. 1173

                5930      5940      5950      5960      5970      5980      5990      6000
          ....|....|....|....|....|....|....|....|....|....|....|....|....|....|....|....|
KU70cDNA  ATTTATTTACCCCAGTGATGAG~~~~~~~~~~~~~~~~~~~~~~~~~~~~~~~~~~~~~~~~~~~~~~~~~~~~~~~~~~ 1198
KU70-A    ......................GTAATAACACTTCCTTATATTGGTCATCGCCAAGGGTACTTTGTTCTTTTGTGCATAA 6000
HY1 cDNA  ......................~~~~~~~~~~~~~~~~~~~~~~~~~~~~~~~~~~~~~~~~~~~~~~~~~~~~~~~~~~ 1195

                6010      6020      6030      6040      6050      6060      6070      6080
          ....|....|....|....|....|....|....|....|....|....|....|....|....|....|....|....|
KU70cDNA  ~~~~~~~~~~~~~~~~~~~~~~~~~~~~~~~~~~~~~~~~~~~~~~~~~~~~~~~~~~~~~~~~~~~~~~~~~~~~~~~~ 1198
KU70-A    CGTTCATCATTCGAATACTATTTTTATGTTGTTTCTACAGAGTCCAGATCATTTATGTCTCTACTCTCTGTCTTTAGACT 6080
HY1 cDNA  ~~~~~~~~~~~~~~~~~~~~~~~~~~~~~~~~~~~~~~~~~~~~~~~~~~~~~~~~~~~~~~~~~~~~~~~~~~~~~~~~ 1195

                6090      6100      6110      6120      6130      6140      6150      6160
          ....|....|....|....|....|....|....|....|....|....|....|....|....|....|....|....|
KU70cDNA  ~~~~~~~~~~~~~~~~~~~~~~~~~~~~~~~~~~~~~~~~~~~~~~~~~~~~~~~~~~~~~~~~~~~~~~~~~~~~~~~~ 1198
KU70-A    GATAATCTTGTTCATAAGCTTCCAATATTTTCTGTGATGAGAATGTTTTCATTATATGTGCTTCTCTAACTTTAGGTAGA 6160
HY1 cDNA  ~~~~~~~~~~~~~~~~~~~~~~~~~~~~~~~~~~~~~~~~~~~~~~~~~~~~~~~~~~~~~~~~~~~~~~~~~~~~~~~~ 1195
```

```
                     6170      6180      6190      6200      6210      6220      6230      6240
           ....|....|....|....|....|....|....|....|....|....|....|....|....|....|....|....|
KU70cDNA   ~~~~~~~~~~~~~~~~~~~~~~~~~~~~~~~~~~~~~~~~~~~~~~~~~~~~~~~~~~~~~~~~~~~~~~~~~~~~~~~~ 1198
KU70-A     TAATCAATAGTTGTAAACTTTCAATATTTTCCTTGATGGGAATGTTTTTCATTATATGTGAGGACACTTTTGTAGTTTGA 6240
HY1 cDNA   ~~~~~~~~~~~~~~~~~~~~~~~~~~~~~~~~~~~~~~~~~~~~~~~~~~~~~~~~~~~~~~~~~~~~~~~~~~~~~~~~ 1195

                     6250      6260      6270      6280      6290      6300      6310      6320
           ....|....|....|....|....|....|....|....|....|....|....|....|....|....|....|....|
KU70cDNA   ~~~~~~~~~~~~~~~~~~~~~~~~~~~~~~~~~~~~~~~~~~~~~~~~~~~~~~~~~~~~~~~~~~~~~~~~~~~~~~~~ 1198
KU70-A     CTTGGTGGTTCTAGTGCATACTACTTCTTGTTCAGCCTGAAGGAGTAGTTCTCTCAGTGAAAATATAATGCTTCGCTCAG 6320
HY1 cDNA   ~~~~~~~~~~~~~~~~~~~~~~~~~~~~~~~~~~~~~~~~~~~~~~~~~~~~~~~~~~~~~~~~~~~~~~~~~~~~~~~~ 1195

                     6330      6340      6350      6360      6370      6380      6390      6400
           ....|....|....|....|....|....|....|....|....|....|....|....|....|....|....|....|
KU70cDNA   ~~~~~~~~~~~~~~~~~~~~~~~~~~~~~~~~~~~~~~~~~~~~~~~~~~~~~~~~~~~~~~~~~~~~~~~~~~~~~~~~ 1198
KU70-A     TGATTAGTTACTTTGTCCTATTAGTAACTAAAATAGATCTCTTGAGAATATTTAGGGTTGTTGAATGATGGGTGCATGCT 6400
HY1 cDNA   ~~~~~~~~~~~~~~~~~~~~~~~~~~~~~~~~~~~~~~~~~~~~~~~~~~~~~~~~~~~~~~~~~~~~~~~~~~~~~~~~ 1195

                     6410      6420      6430      6440      6450      6460      6470      6480
           ....|....|....|....|....|....|....|....|....|....|....|....|....|....|....|....|
KU70cDNA   ~~~~~~~~~~~~~~~~~~~~~~~~~~~~~~~~~~~~~~~~~~~~~~~~~~~~~~~~~~~~~~~~~~~~~~~~~~~~~~~~ 1198
KU70-A     AGATTTACTTCCTTTGCTTTGATGCAGCAAATATTTGGAAGCACTCACATTTTCATGCTTTACATAGCTTGATGCTGAAC 6480
HY1 cDNA   ~~~~~~~~~~~~~~~~~~~~~~~~~~~~~~~~~~~~~~~~~~~~~~~~~~~~~~~~~~~~~~~~~~~~~~~~~~~~~~~~ 1195

                     6490      6500      6510      6520      6530      6540      6550      6560
           ....|....|....|....|....|....|....|....|....|....|....|....|....|....|....|....|
KU70cDNA   ~~~~~~~~~~~~~~~~~~~~~~~~~~~~~~~~~~~~~~~~~~~~~~~~~~~~~~~~~~CAAATATTCGGAAGCACTCGTG 1220
KU70-A     ATAACTAGGGCTGTTGAATAATGGGTGGATGCGATTTACCTCCTTTGCTTTGATGCAG.................T.... 6560
HY1 cDNA   ~~~~~~~~~~~~~~~~~~~~~~~~~~~~~~~~~~~~~~~~~~~~~~~~~~~~~~~~~~...................... 1217

                     6570      6580      6590      6600      6610      6620      6630      6640
           ....|....|....|....|....|....|....|....|....|....|....|....|....|....|....|....|
KU70cDNA   TGTTTGTTGCTTTACATAGCTCGATGCCGCGTCTTGGAAG~~~~~~~~~~~~~~~~~~~~~~~~~~~~~~~~~~~~~~~~ 1260
KU70-A     ....C......................T..........C.GTTAGTCACTATCGCTTATTCCCGATCCTGCCACAACTTC 6640
HY1 cDNA   ...........................T............~~~~~~~~~~~~~~~~~~~~~~~~~~~~~~~~~~~~~~~~ 1257

                     6650      6660      6670      6680      6690      6700      6710      6720
           ....|....|....|....|....|....|....|....|....|....|....|....|....|....|....|....|
KU70cDNA   ~~~~~~~~~~~~~~~~~~~~~~~~~~~~~~~~~~~~~~~~~~~~~~~~~~~~~~~~~~~~~~~~~~~~~~~~~~~~~~~~ 1260
KU70-A     TATGACCACTTCAGGAAGAAAGCTTGTCCGAGAACTGAAACCTATGAATGTGCATAAAGCTATCCTGAATCATAAGGATG 6720
HY1 cDNA   ~~~~~~~~~~~~~~~~~~~~~~~~~~~~~~~~~~~~~~~~~~~~~~~~~~~~~~~~~~~~~~~~~~~~~~~~~~~~~~~~ 1257

                     6730      6740      6750      6760      6770      6780      6790      6800
           ....|....|....|....|....|....|....|....|....|....|....|....|....|....|....|....|
KU70cDNA   ~~~~~~~~~~~~~~~~~~~~~~~~~~~~~~~~~~~~~~~~~~~~~~~~~~~~~~~~~~~~~~~~~~~~~~~~~~~~~~~~ 1260
KU70-A     CACTGAAAAGAACTAGTATTGGATTATTGGTTCTAATTAGTATGGACTATTGGTTCTCACATTACATTCTCTCGTTGTTT 6800
HY1 cDNA   ~~~~~~~~~~~~~~~~~~~~~~~~~~~~~~~~~~~~~~~~~~~~~~~~~~~~~~~~~~~~~~~~~~~~~~~~~~~~~~~~ 1257

                     6810      6820      6830      6840      6850      6860      6870      6880
           ....|....|....|....|....|....|....|....|....|....|....|....|....|....|....|....|
KU70cDNA   ~~~~~~~~~~~~~~~~~~~~~~~~~~~~~~~~~~~~~~~~~~~~~~~~~~~~~~~~~~~~~~~~~~~~~~~~~~~~~~~~ 1260
KU70-A     GGGATAGTTTCTGGGGATAACCACTTGGGTATGATGTTGAGGTATTATTCACGACAAGTAAATGAATTCGAATACACGTG 6880
HY1 cDNA   ~~~~~~~~~~~~~~~~~~~~~~~~~~~~~~~~~~~~~~~~~~~~~~~~~~~~~~~~~~~~~~~~~~~~~~~~~~~~~~~~ 1257

                     6890      6900      6910      6920      6930      6940      6950      6960
           ....|....|....|....|....|....|....|....|....|....|....|....|....|....|....|....|
KU70cDNA   ~~~~~~~~~~~~~~~~~~~~~~~~~~~~~~~~~~~~~~~~~~~~~~~~~~~~~~~~~~~~~~~~~~~~~~~~~~~~~~~~ 1260
KU70-A     GTTGGAACATTGTTTGTTGTAGTTTTGGGTTTGTTTGTCATGTCTTCTGTTGAAATAATTTTTGATGGTGTGATCTGTAG 6960
HY1 cDNA   ~~~~~~~~~~~~~~~~~~~~~~~~~~~~~~~~~~~~~~~~~~~~~~~~~~~~~~~~~~~~~~~~~~~~~~~~~~~~~~~~ 1257

                     6970      6980      6990      7000      7010      7020      7030      7040
           ....|....|....|....|....|....|....|....|....|....|....|....|....|....|....|....|
KU70cDNA   ~~~~~~~~~~~~~~~~~~~~~~~~~~~~~~~~~~~~~~~~~~~~~~~~~~~~~~~~~~~~~~~~~~~~~~~~~~~~~~~~ 1260
KU70-A     TCTGCTGGCTGTTTGGAAAATGAAAGGATAAAGGAGTTGTGGCTTATGATGTGAAAAGCATGGTAATGGGTGATACGGTA 7040
HY1 cDNA   ~~~~~~~~~~~~~~~~~~~~~~~~~~~~~~~~~~~~~~~~~~~~~~~~~~~~~~~~~~~~~~~~~~~~~~~~~~~~~~~~ 1257

                     7050      7060      7070      7080      7090      7100      7110      7120
           ....|....|....|....|....|....|....|....|....|....|....|....|....|....|....|....|
KU70cDNA   ~~~~~~~~~~~~~~~~~~~~~~~~~~~~~~~~~~~~~~~~~~~~~~~~~~~~~~~~~~~~~~~~~~~~~~~~~~~~~~~~ 1260
KU70-A     CACAACAGGGAAAGTTATTTCTTTTAGGTCACATATCTAAAATGGGTTTTTGGTTGGCACTCTTCTAAACTGTAGGATCC 7120
HY1 cDNA   ~~~~~~~~~~~~~~~~~~~~~~~~~~~~~~~~~~~~~~~~~~~~~~~~~~~~~~~~~~~~~~~~~~~~~~~~~~~~~~~~ 1257

                     7130      7140      7150      7160      7170      7180      7190      7200
           ....|....|....|....|....|....|....|....|....|....|....|....|....|....|....|....|
KU70cDNA   ~~~~~~~~~~~~~~~~~~~~~~~~~~~~~~~~~~~~~~~~~~~~~~~~~~~~~~~~~~~~~~~~~~~~~~~~~~~~~~~~ 1260
KU70-A     CAGTCAATGACATTTACCTGTTTTCCCCTTCTATGCCTAATTGGTTCCTTGTAACTTCTGCTGGGCTGTATATTTTTTTT 7200
HY1 cDNA   ~~~~~~~~~~~~~~~~~~~~~~~~~~~~~~~~~~~~~~~~~~~~~~~~~~~~~~~~~~~~~~~~~~~~~~~~~~~~~~~~ 1257
```

```
                7210      7220      7230      7240      7250      7260      7270      7280
          ....|....|....|....|....|....|....|....|....|....|....|....|....|....|....|....|
KU70cDNA  ~~~~~~~~~~~~~~~~~~~~~~~~~~~~~~~~~~~~~~~~~~~~~~~~~~~~~~~~~~~~~~~~~~~~~~~~~~~~~~~~ 1260
KU70-A    AGGTTGTCACTTTGGCATGTATGTTCAACTTACTCGGCTCTTTTTGTGTATGGGAGGTGCCAAAGTCAATAAGAGTTTGG 7280
HY1 cDNA  ~~~~~~~~~~~~~~~~~~~~~~~~~~~~~~~~~~~~~~~~~~~~~~~~~~~~~~~~~~~~~~~~~~~~~~~~~~~~~~~~ 1257

                7290      7300      7310      7320      7330      7340      7350      7360
          ....|....|....|....|....|....|....|....|....|....|....|....|....|....|....|....|
KU70cDNA  ~~~~~~~~~~~~~~~~~~~~~~~~~~~~~~~~~~~~~~~~~~~~~~~~~~~~~~~~~~~~~~~~~~~~~~~~~~~~~~~~ 1260
KU70-A    CCACACCAACAGATAATCACTTATCTTATTAACATAATGTTCTGTAGCAACCCATCATCTCAAACCTTTCTATGTTATTA 7360
HY1 cDNA  ~~~~~~~~~~~~~~~~~~~~~~~~~~~~~~~~~~~~~~~~~~~~~~~~~~~~~~~~~~~~~~~~~~~~~~~~~~~~~~~~ 1257

                7370      7380      7390      7400      7410      7420      7430      7440
          ....|....|....|....|....|....|....|....|....|....|....|....|....|....|....|....|
KU70cDNA  ~~~~~~~~~~~~~~~~~~~~~~~~~~~~~~~~~~~~~~~~~~~~~~~~~~~~~~~~~~~~~~~~~~~~~~~~~~~~~~~~ 1260
KU70-A    CTACTACTCCCTCTGTTCACTTTTGTAAGTCGTTTAAGACAGCTGAAATTGAACTGTTTTAGATGCTTTCCTAAATGTCT 7440
HY1 cDNA  ~~~~~~~~~~~~~~~~~~~~~~~~~~~~~~~~~~~~~~~~~~~~~~~~~~~~~~~~~~~~~~~~~~~~~~~~~~~~~~~~ 1257

                7450      7460      7470      7480      7490      7500      7510      7520
          ....|....|....|....|....|....|....|....|....|....|....|....|....|....|....|....|
KU70cDNA  ~~~~~~~~~~~~~~~~~~~~~~~~~~~~~~~~~~~~~~~~~~~~~~~~~~~~~~~~~~~~~~~~~~~~~~~~~~~~~~~~ 1260
KU70-A    AAAACGTCTTACAAAAGTGAACGGAGGGAGTAGCTATCATGTGTCATGTTGTCCCCCTGTTTTTGAGTCGAACGACTAGT 7520
HY1 cDNA  ~~~~~~~~~~~~~~~~~~~~~~~~~~~~~~~~~~~~~~~~~~~~~~~~~~~~~~~~~~~~~~~~~~~~~~~~~~~~~~~~ 1257

                7530      7540      7550      7560      7570      7580      7590      7600
          ....|....|....|....|....|....|....|....|....|....|....|....|....|....|....|....|
KU70cDNA  ~~~~~~~~~~~~~~~~~~~~~~~~~~~~~~~~~~~~~~~~~~~~~~~~~~~~~~~~~~~~~~~~~~~~~~~~~~~~~~~~ 1260
KU70-A    CGCGACTAGTCGATGAGTTGTAACTGGAAGTCGTCAAACACCTCTACTAGTCGATCGGCACGGCACGACTCGTCAAGAGT 7600
HY1 cDNA  ~~~~~~~~~~~~~~~~~~~~~~~~~~~~~~~~~~~~~~~~~~~~~~~~~~~~~~~~~~~~~~~~~~~~~~~~~~~~~~~~ 1257

                7610      7620      7630      7640      7650      7660      7670      7680
          ....|....|....|....|....|....|....|....|....|....|....|....|....|....|....|....|
KU70cDNA  ~~~~~~~~~~~~~~~~~~~~~~~~~~~~~~~~~~~~~~~~~~~~~~~~~~~~~~~~~~~~~~~~~~~~~~~~~~~~~~~~ 1260
KU70-A    CGCTACCCCAGAATGACTTGTCGACTCGAAAACCTTGCATGTTGTACATGTTCTCATGTGTTTATTCAAACAAGTGGCTG 7680
HY1 cDNA  ~~~~~~~~~~~~~~~~~~~~~~~~~~~~~~~~~~~~~~~~~~~~~~~~~~~~~~~~~~~~~~~~~~~~~~~~~~~~~~~~ 1257

                7690      7700      7710      7720      7730      7740      7750      7760
          ....|....|....|....|....|....|....|....|....|....|....|....|....|....|....|....|
KU70cDNA  ~~~~~~~~~~~~~~~~~~~~~~~~~~~~~~~~~~~~~~~~~~~~~~~~~~~~~~~~~~~~~~~~~~~~~~~~~~~~~~~~ 1260
KU70-A    GGCATAGACTAAGGTGTTCTGTAACAAGATTACAAGAGTACACATGCTTATAGCATCATAAATGTAGTGTTTCGTGTTTC 7760
HY1 cDNA  ~~~~~~~~~~~~~~~~~~~~~~~~~~~~~~~~~~~~~~~~~~~~~~~~~~~~~~~~~~~~~~~~~~~~~~~~~~~~~~~~ 1257

                7770      7780      7790      7800      7810      7820      7830      7840
          ....|....|....|....|....|....|....|....|....|....|....|....|....|....|....|....|
KU70cDNA  ~~~~~~~~~~~~~~~~~~~~~~~~~~~~~~~~~~~~~~~~~~~~~~~~~~~~~~~~~~GTTTGCACTTGCGTTTTACGGG 1282
KU70-A    TTTTTACGGTTATATGATCCGTGCTACAAACTTAGAATTATGATATGTATGATTTCAG...................... 7840
HY1 cDNA  ~~~~~~~~~~~~~~~~~~~~~~~~~~~~~~~~~~~~~~~~~~~~~~~~~~~~~~~~~~...................... 1279

                7850      7860      7870      7880      7890      7900      7910      7920
          ....|....|....|....|....|....|....|....|....|....|....|....|....|....|....|....|
KU70cDNA  ACCCCAACTCGCCCACGGCTTGTAGCCCTTGTTGCACAA~~~~~~~~~~~~~~~~~~~~~~~~~~~~~~~~~~~~~~~~~ 1321
KU70-A    .......................................GTAAGATGTATTTTCCATGTTTATGTTTGTTTTGCAATGAT 7920
HY1 cDNA  .......................................~~~~~~~~~~~~~~~~~~~~~~~~~~~~~~~~~~~~~~~~~ 1318

                7930      7940      7950      7960      7970      7980      7990      8000
          ....|....|....|....|....|....|....|....|....|....|....|....|....|....|....|....|
KU70cDNA  ~~~~~~~~~~~~~~~~~~~~~~~~~~~~~~~~~~~~~~~~~~~~~~~~~~~~~~~~~~~~~~~~~~~~~~~~~~~~~~~~ 1321
KU70-A    TACTATGGTAAACTTGTTAGTCTGGGTTACGTGTATGATATGAGCAACCTCTTTAAGGACAAATCTATTCAATGTAACAG 8000
HY1 cDNA  ~~~~~~~~~~~~~~~~~~~~~~~~~~~~~~~~~~~~~~~~~~~~~~~~~~~~~~~~~~~~~~~~~~~~~~~~~~~~~~~~ 1318

                8010      8020      8030      8040      8050      8060      8070      8080
          ....|....|....|....|....|....|....|....|....|....|....|....|....|....|....|....|
KU70cDNA  ~~~~~~~~~~~~~~~~~~~~~~~~~~~~~~~~~~~~~~~~~~~~~~~~~~~~~~~~~~~~~~~~~~~~~~~~~~~~~~~~ 1321
KU70-A    TGCAAATGGTGTTAATTGCACACAAAAACCCCTTCTTATTTTCCATAAAAAATGATTTCTGGCAAGTTGATGTATAACAT 8080
HY1 cDNA  ~~~~~~~~~~~~~~~~~~~~~~~~~~~~~~~~~~~~~~~~~~~~~~~~~~~~~~~~~~~~~~~~~~~~~~~~~~~~~~~~ 1318

                8090      8100      8110      8120      8130      8140      8150      8160
          ....|....|....|....|....|....|....|....|....|....|....|....|....|....|....|....|
KU70cDNA  ~~~~~~~~~~~~~~~~~~~~~~~~~~~~~~~~~~~~~~~~~~~~~~~~~~~~~~~~~~~~~~~~~~~~~~~~~~~~~~~~ 1321
KU70-A    GATTGTTCTATGCATCACACGATAGCTTATCTCTGGGCAACCACAGAACTATGTCAATATCTGGGTTTGATTACATGAAC 8160
HY1 cDNA  ~~~~~~~~~~~~~~~~~~~~~~~~~~~~~~~~~~~~~~~~~~~~~~~~~~~~~~~~~~~~~~~~~~~~~~~~~~~~~~~~ 1318

                8170      8180      8190      8200      8210      8220      8230      8240
          ....|....|....|....|....|....|....|....|....|....|....|....|....|....|....|....|
KU70cDNA  ~~~~~~~~~~~~~~~~~~~~~~~~~~~~~~~~~~~~~~~~~~~~~~~~~~~~~~~~~~~~~~~~~~~~~~~~~~~~~~~~ 1321
KU70-A    AAAAACGACTAATCTTCTTGCTTCGTTTCCCTGAAAATTTTCTACTTGCTTCATTCAACTAATTACTTGGATATTTCTCA 8240
```

```
HY1 cDNA  ~~~~~~~~~~~~~~~~~~~~~~~~~~~~~~~~~~~~~~~~~~~~~~~~~~~~~~~~~~~~~~~~~~~~~~~~~~~~~~~~ 1318

                   8250      8260      8270      8280      8290      8300      8310      8320
          ....|....|....|....|....|....|....|....|....|....|....|....|....|....|....|....|
KU70cDNA  ~~~~~~~~~~~~~~~~~~~~~~~~~~~~~~~~~~~~~~~~~~~~~~~~~~~~~~~~~~~~~~~~~~~~~~~~~~~~~~~~ 1321
KU70-A    GTGAGATAATGAGGTAAACTGTAATAAACCCGAAATGACTTGGCTCTACAAACACCTCTTGTAGTGATACTCAAGTTTCC 8320
HY1 cDNA  ~~~~~~~~~~~~~~~~~~~~~~~~~~~~~~~~~~~~~~~~~~~~~~~~~~~~~~~~~~~~~~~~~~~~~~~~~~~~~~~~ 1318

                   8330      8340      8350      8360      8370      8380      8390      8400
          ....|....|....|....|....|....|....|....|....|....|....|....|....|....|....|....|
KU70cDNA  ~~~~~~~~~~~~~~~~~~~~~~~~~~~~~~~~~~~~~~~~~~~~~~~~~~~~~~~~~~~~~~~~~~~~~~~~~~~~~~~~ 1321
KU70-A    AATCTTTCATGTTCCAGCTTCTCCCCATTTCTCACTTATAGGATAAGACTCGAGTGGATGGTGTGAAAATTATCTACTCC 8400
HY1 cDNA  ~~~~~~~~~~~~~~~~~~~~~~~~~~~~~~~~~~~~~~~~~~~~~~~~~~~~~~~~~~~~~~~~~~~~~~~~~~~~~~~~ 1318

                   8410      8420      8430      8440      8450      8460      8470      8480
          ....|....|....|....|....|....|....|....|....|....|....|....|....|....|....|....|
KU70cDNA  ~~~~~~~~~~~~~~~~~~~~~~~~~~~~~~~~~~~~~~~~~~~~~~~~~~~~~~~~~~~~~~~~~~~~~~~~~~~~~~~~ 1321
KU70-A    TGCATCACATTCTACTTTTTTGTATTATGCATCATGATTTTTCTGTCTGTGGCTCTGAACTTCTTGGATTTACAATTTCA 8480
HY1 cDNA  ~~~~~~~~~~~~~~~~~~~~~~~~~~~~~~~~~~~~~~~~~~~~~~~~~~~~~~~~~~~~~~~~~~~~~~~~~~~~~~~~ 1318

                   8490      8500      8510      8520      8530      8540      8550      8560
          ....|....|....|....|....|....|....|....|....|....|....|....|....|....|....|....|
KU70cDNA  ~~~~~~~~~~~~~~~~~~~~~~~~~~~~~~~~~~~~~~~~~~~~~~~~~~~GAAGAGGTTATTTCTTCGAGTGGTCAAGA 1350
KU70-A    TAACCCCCAACTATTTTCCTTCCCAAAATAAACACTGGGATTGAAAAACAG............................. 8560
HY1 cDNA  ~~~~~~~~~~~~~~~~~~~~~~~~~~~~~~~~~~~~~~~~~~~~~~~~~~~............................. 1347

                   8570      8580      8590      8600      8610      8620      8630      8640
          ....|....|....|....|....|....|....|....|....|....|....|....|....|....|....|....|
KU70cDNA  TGAGCCACCTGGAACGCACATGATCTATCTTCCATATTCGGATGATGTTAGATATCCTGAAGAG~~~~~~~~~~~~~~~~ 1414
KU70-A    ..............T.....................C...........................GTAACCACTGGTGCTG 8640
HY1 cDNA  ..............T.................................................~~~~~~~~~~~~~~~~ 1411

                   8650      8660      8670      8680      8690      8700      8710      8720
          ....|....|....|....|....|....|....|....|....|....|....|....|....|....|....|....|
KU70cDNA  ~~~~~~~~~~~~~~~~~~~~~~~~~~~~~~~~~~~~~~~~~~~~~~~~~~~~GTTCATCTGACTTCTGGGGATGCACCTC 1442
KU70-A    CATTTGTATTTTCCCTTCTTAAGCAACAGAATGATTTTACACTTATGTGCAG............................ 8720
HY1 cDNA  ~~~~~~~~~~~~~~~~~~~~~~~~~~~~~~~~~~~~~~~~~~~~~~~~~~~~............................ 1439

                   8730      8740      8750      8760      8770      8780      8790      8800
          ....|....|....|....|....|....|....|....|....|....|....|....|....|....|....|....|
KU70cDNA  GCGCTACAGATGAGCAAATAAAGAAAGCTTCGAATCTGTTGAGACGTATTGACCTGAAGCATTTCTCAGTAAGCCATTTT 1522
KU70-A    .......................................................................C........ 8800
HY1 cDNA  ................................................................................ 1519

                   8810      8820      8830      8840      8850      8860      8870      8880
          ....|....|....|....|....|....|....|....|....|....|....|....|....|....|....|....|
KU70cDNA  GCTAACCCAG~~~~~~~~~~~~~~~~~~~~~~~~~~~~~~~~~~~~~~~~~~~~~~~~~~~~~~~~~~~~~~~~~~~~ 1532
KU70-A    ..........GTAAATCACTACCTAGAATTGATTTTAGTTTTTGTTAGAACTACTCGTAACTTATGTGTGTGTTCCACTT 8880
HY1 cDNA  ..........~~~~~~~~~~~~~~~~~~~~~~~~~~~~~~~~~~~~~~~~~~~~~~~~~~~~~~~~~~~~~~~~~~~~~~ 1529

                   8890      8900      8910      8920      8930      8940      8950      8960
          ....|....|....|....|....|....|....|....|....|....|....|....|....|....|....|....|
KU70cDNA  ~~~~~~~~~~~~~~~~~~~~~~~~~~~~~~~~~~~~~~~~~~~~~~~~~~~~~~~~~~~~~~~~~~~~~~~~~~~~~~~~ 1532
KU70-A    GTAGATTTGCCTCTTATCTTACAATCTATATAGACCAGGATTGCTTGCTGATGTGTTGTAGCTCAATATCTTGTTGGGTT 8960
HY1 cDNA  ~~~~~~~~~~~~~~~~~~~~~~~~~~~~~~~~~~~~~~~~~~~~~~~~~~~~~~~~~~~~~~~~~~~~~~~~~~~~~~~~ 1529

                   8970      8980      8990      9000      9010      9020      9030      9040
          ....|....|....|....|....|....|....|....|....|....|....|....|....|....|....|....|
KU70cDNA  ~~~~~~~~~~~~~~~~~~~~~~~~~~~~~~~~~~~~~~~~~~~~~~~~~~~~~~~~~~~~~~~~~~~~~~~~~~~~~~~~ 1532
KU70-A    AGCATGGCAATGAGCATTAAGAACTAAGTTGATGCACAGACTCTAAATTCAGTGAATTTCTCATTATTCCGATTGTGGTT 9040
HY1 cDNA  ~~~~~~~~~~~~~~~~~~~~~~~~~~~~~~~~~~~~~~~~~~~~~~~~~~~~~~~~~~~~~~~~~~~~~~~~~~~~~~~~ 1529

                   9050      9060      9070      9080      9090      9100      9110      9120
          ....|....|....|....|....|....|....|....|....|....|....|....|....|....|....|....|
KU70cDNA  ~~~~~~~~~~~~~~~~~~~~GATTGCAAAAACACTATGGGATTTTGGAGGCCTTAGCTTTAGGTGAGGATGAGATGCCGG 1592
KU70-A    TCTGTTCTGTCATGGAACAG....A...........C........................................... 9120
HY1 cDNA  ~~~~~~~~~~~~~~~~~~~~............................................................ 1589

                   9130      9140      9150      9160      9170      9180      9190      9200
          ....|....|....|....|....|....|....|....|....|....|....|....|....|....|....|....|
KU70cDNA  ATATGAAGGATGAGACCCTGCCCGATGAAGAAGGCTTGGCTAG~~~~~~~~~~~~~~~~~~~~~~~~~~~~~~~~~~~~~ 1635
KU70-A    ....A......................................GTACATGTTTTCTTTTGTCAATTGTGTATGCTACATC 9200
HY1 cDNA  ....A......................................~~~~~~~~~~~~~~~~~~~~~~~~~~~~~~~~~~~~~ 1632

                   9210      9220      9230      9240      9250      9260      9270      9280
          ....|....|....|....|....|....|....|....|....|....|....|....|....|....|....|....|
KU70cDNA  ~~~~~~~~~~~~~~~~~~~~~~~~~~~~~~~~~~~~~~~~~~~~~~~~~~~~~~~~~~~~~~~~~~~~~~~~~~~~~~~~ 1635
```

```
KU70-A     CTAATTCCGCCATTTCTTTCTTGCAAATGCAACTTATTGAATAACTATAGTCAACCATCTTGGTGTTCCTGCGTCCAAAC 9280
HY1 cDNA   ~~~~~~~~~~~~~~~~~~~~~~~~~~~~~~~~~~~~~~~~~~~~~~~~~~~~~~~~~~~~~~~~~~~~~~~~~~~~~~~~ 1632

                    9290      9300      9310      9320      9330      9340      9350      9360
           ....|....|....|....|....|....|....|....|....|....|....|....|....|....|....|....|
KU70cDNA   ~~~~~~~~~~~~~~~~~~~~~~~~~~~~~~~~~~~~~~~~~~~~~~~~~~~~~~~~~~~~~~~~~~~~~~~~~~~~~~~~ 1635
KU70-A     AGGAACAGTGATGATGAAAACATAATTTAGTGCATGCTGAAATAAGCTTAAGATATTTTTATTCTTTATACGTATAAGAA 9360
HY1 cDNA   ~~~~~~~~~~~~~~~~~~~~~~~~~~~~~~~~~~~~~~~~~~~~~~~~~~~~~~~~~~~~~~~~~~~~~~~~~~~~~~~~ 1632

                    9370      9380      9390      9400      9410      9420      9430      9440
           ....|....|....|....|....|....|....|....|....|....|....|....|....|....|....|....|
KU70cDNA   ~~~~~~~~~~~~~~~~~~~~~~~~~~~~~~~~~~~~~~~~~~~~~~~~~~~~~~~~~~~~~~~~~~~~~~~~~~~~~~~~ 1635
KU70-A     AGAACAATCTAGGGCATGCTTAAATAAGCTGAAGGTTTACTTATCAAACGTATTGTTTGTAGTATAAAAGAAACATACAA 9440
HY1 cDNA   ~~~~~~~~~~~~~~~~~~~~~~~~~~~~~~~~~~~~~~~~~~~~~~~~~~~~~~~~~~~~~~~~~~~~~~~~~~~~~~~~ 1632

                    9450      9460      9470      9480      9490      9500      9510      9520
           ....|....|....|....|....|....|....|....|....|....|....|....|....|....|....|....|
KU70cDNA   ~~~~~~~~~~~~~~~~~~~~~~~~~~~~~~~~~~~~~~~~~~~~~~~~~GCCAGGAGTAGTTAAAGCTATAGAAGAATTT 1666
KU70-A     CAGTCGTCCCGTACCTTATCGTGGAATATTTCCTATACTCTTATCTTAG............................... 9520
HY1 cDNA   ~~~~~~~~~~~~~~~~~~~~~~~~~~~~~~~~~~~~~~~~~~~~~~~~~............................... 1663

                    9530      9540      9550      9560      9570      9580      9590      9600
           ....|....|....|....|....|....|....|....|....|....|....|....|....|....|....|....|
KU70cDNA   AAGGCTGCAGTGTTTGGTGAAAATTATGACCAAGAGGAAGCTGAGGCAGCAGCAGCAAAAGGTGGGGCCTCAAAGAAGAG 1746
KU70-A     ................................................................................ 9600
HY1 cDNA   ................................................................................ 1743

                    9610      9620      9630      9640      9650      9660      9670      9680
           ....|....|....|....|....|....|....|....|....|....|....|....|....|....|....|....|
KU70cDNA   GAAAGCAATTGCTGATGCAGCTTCACAGAAAAGCGCGGCCTATGATTGGGCAGACCTTGCAGATAATGGGAAG~~~~~~~ 1819
KU70-A     ............G.......................A..T.................................GTGAGTA 9680
HY1 cDNA   .........................................................................~~~~~~~ 1816

                    9690      9700      9710      9720      9730      9740      9750      9760
           ....|....|....|....|....|....|....|....|....|....|....|....|....|....|....|....|
KU70cDNA   ~~~~~~~~~~~~~~~~~~~~~~~~~~~~~~~~~~~~~~~~~~~~~~~~~~~~~~~~~~~~~~~~~~~~~~~~~~~~~~~~ 1819
KU70-A     GTCAGGTTATATTACAAAGATTATGTTTACATGAAAAAAAGTGCTCAATACGCAAACTGTTGCGATAAATTGGTCTTTTG 9760
HY1 cDNA   ~~~~~~~~~~~~~~~~~~~~~~~~~~~~~~~~~~~~~~~~~~~~~~~~~~~~~~~~~~~~~~~~~~~~~~~~~~~~~~~~ 1816

                    9770      9780      9790      9800      9810      9820      9830      9840
           ....|....|....|....|....|....|....|....|....|....|....|....|....|....|....|....|
KU70cDNA   ~~~~~~~~~~~~~~~~~~~~~~~~~~~~~~~~~~~~~~~~~~~~~~~~~~~~~~~~~~~~~~~~~~~~~~~~~~~~~~~~ 1819
KU70-A     ATAGTCACCCACTCTACAACCCATGCTGTAATTCCAAAAATAGTTAGCATAATATGCCAGATCTTGGCATTTTAGGAACT 9840
HY1 cDNA   ~~~~~~~~~~~~~~~~~~~~~~~~~~~~~~~~~~~~~~~~~~~~~~~~~~~~~~~~~~~~~~~~~~~~~~~~~~~~~~~~ 1816

                    9850      9860      9870      9880      9890      9900      9910      9920
           ....|....|....|....|....|....|....|....|....|....|....|....|....|....|....|....|
KU70cDNA   ~~~~~~~~~~~~~~~~~~~~~~~~~~~~~~~~~~~~~~~~~~~~~~~~~~~~~~~~~~~~~~~~~~~~~~~~~~~~~~~~ 1819
KU70-A     ATAGTTCCACCAAGAATCCGCAGAAAATGAGGTTATACATGTCAAACTATGCATGCACCTTTGAAACATGAATTACATTT 9920
HY1 cDNA   ~~~~~~~~~~~~~~~~~~~~~~~~~~~~~~~~~~~~~~~~~~~~~~~~~~~~~~~~~~~~~~~~~~~~~~~~~~~~~~~~ 1816

                    9930      9940      9950      9960      9970      9980      9990     10000
           ....|....|....|....|....|....|....|....|....|....|....|....|....|....|....|....|
KU70cDNA   ~~~~~~~~~~~~~~~~~~~~~~~~~~~~~~~~~~~~~~~~~~~~~~~~~~~~~~~~~~~~~~~~~~~~~~~~~~~~~~~~ 1819
KU70-A     GGATGTGTTGTTTCTTTGATATCCCTGAATAGCGCCATTCATCCAAACCTTTCTGAGTTAACCACACTGATGTTTGTTTT 10000
HY1 cDNA   ~~~~~~~~~~~~~~~~~~~~~~~~~~~~~~~~~~~~~~~~~~~~~~~~~~~~~~~~~~~~~~~~~~~~~~~~~~~~~~~~ 1816

                   10010     10020     10030     10040     10050     10060     10070     10080
           ....|....|....|....|....|....|....|....|....|....|....|....|....|....|....|....|
KU70cDNA   ~~~~~~~~~~~~~~~~~~~~~~~~~~~CTGAAGGACATGACGGTGATGGGTTTGAAAACTTACCTGACGGCGCATGGCCT 1872
KU70-A     GCAAAATTCTGAATCTTTAATTTGCAG..............A.......A......G..C.................... 10080
HY1 cDNA   ~~~~~~~~~~~~~~~~~~~~~~~~~~~......................A.............................. 1869

                   10090     10100     10110     10120     10130     10140     10150     10160
           ....|....|....|....|....|....|....|....|....|....|....|....|....|....|....|....|
KU70cDNA   CCCAGTTTCTGGCAAGAAAGACGCCATCATCAGCAGGATCTTGACTCATCTAGGCAAGTGAGAGACGAGAATCGTTGGCT 1952
KU70-A     ............T..................................................A..A......A...A.. 10160
HY1 cDNA   ..........................................................                       1930

                   10170     10180     10190     10200     10210     10220
           ....|....|....|....|....|....|....|....|....|....|....|....|....|....
KU70cDNA   GGAATATGCAAGACGCCTAGTGTGCATCCTACGTAGCCATGGATGATAGACCCACCCTCTCTGTTTGGG 2021
KU70-A     ...............T...............T..................................... 10229
HY1 cDNA
```
