# Supplementary material for: Factors Affecting the Radiosensitivity of Hexaploid Wheat to γ-Irradiation: Radiosensitivity of Hexaploid Wheat (Triticum aestivum L.)
Source: PLoS One. 2016 Aug 23;11(8):e0161700. doi: 10.1371/journal.pone.0161700 (PMC4995049; doi:10.1371/journal.pone.0161700)
Supplement: S2 Fig — 133 bp fragment located between 601 bp and 733 bp was retained in the mRNA sequence in HY1. (PDF) [file pone.0161700.s002.pdf]

```
                   10        20        30        40        50        60        70        80
           ....|....|....|....|....|....|....|....|....|....|....|....|....|....|....|....|
KU70cDNA   ATGGACCTGGACCCCGAGGGCATCTTCCGCGACGACAGCGACGAGGACGACGACAACCTCCATGAGAGGGAGGCCAACAA 80
HY1 cDNA   ................................................................................ 80

                   90       100       110       120       130       140       150       160
           ....|....|....|....|....|....|....|....|....|....|....|....|....|....|....|....|
KU70cDNA   GGAGATGGTCGTCTACCTCATAGACGCCTCGCCCAAGATGTTCACACCCGCCAACGCCGCCAAGCCAGATGAAAAGCAGG 160
HY1 cDNA   .............................................C..........................G..T.... 160

                  170       180       190       200       210       220       230       240
           ....|....|....|....|....|....|....|....|....|....|....|....|....|....|....|....|
KU70cDNA   AGACACATTTCCATACCATAGTGAACTGCATCACGCAGTCTCTGAAGACGCAGATTATCGGGAGATCCCGTGATGAAGTT 240
HY1 cDNA   .................................................A.............................. 240

                  250       260       270       280       290       300       310       320
           ....|....|....|....|....|....|....|....|....|....|....|....|....|....|....|....|
KU70cDNA   GCAATATGCTTCTTTAACACCAAAGAAAAGAAAAATTTACAGGAGCTGGCTGGTGTATATGTTTACAATGTCACAGAAAG 320
HY1 cDNA   ..............C...............................................................C. 320

                  330       340       350       360       370       380       390       400
           ....|....|....|....|....|....|....|....|....|....|....|....|....|....|....|....|
KU70cDNA   AGAGCAACTTGATAGACCTGATGCAAGACTGATTAAAGAATTTTCTTGTGTAGAAGATTCTTTTATGAATACCATTGGAA 400
HY1 cDNA   ...............G.........G.............................................A........ 400

                  410       420       430       440       450       460       470       480
           ....|....|....|....|....|....|....|....|....|....|....|....|....|....|....|....|
KU70cDNA   GCCGGTATGGAATAACCTCTGGATCTAGAGAGAATACCCTGTACAATGCTCTTTGGGTTGCACAGGCACTGCTGCGTAAA 480
HY1 cDNA   ..........................C..................................................... 480

                  490       500       510       520       530       540       550       560
           ....|....|....|....|....|....|....|....|....|....|....|....|....|....|....|....|
KU70cDNA   GGATCTGTGAAGACTGTGAGTAAGAGAATCCTCATATTCACCAATGAGGATGATCCTTTTGGTGGTATTACAGGAGCAGC 560
HY1 cDNA   ................................................................................ 560

                  570       580       590       600       610       620       630       640
           ....|....|....|....|....|....|....|....|....|....|....|....|....|....|....|....|
KU70cDNA   AAAGACTGATATGATTAGGACCACAATTCAACGTGCAAAAG~~~~~~~~~~~~~~~~~~~~~~~~~~~~~~~~~~~~~~~ 601
HY1 cDNA   ...............C.........................TTTGTCTATGACACATACATCTATTCTGTTTTCACACCA 640

                  650       660       670       680       690       700       710       720
           ....|....|....|....|....|....|....|....|....|....|....|....|....|....|....|....|
KU70cDNA   ~~~~~~~~~~ATGCACAAGATCTGGGCCTGTCTATCGAACTTCTTCCATTGAGTAGGCCTGATGAGGATTTCAACATGTC 671
HY1 cDNA   AGCAGCCCAC.GATG...AT.AATATGTAAGTCTC..C.GC..CC.TC.CCCCCC.TATCAG..CCA.A.G.T.AGCAA. 720

                  730       740       750       760       770       780       790       800
           ....|....|....|....|....|....|....|....|....|....|....|....|....|....|....|....|
KU70cDNA   CCTGTTTTATGCAGATTTGATTGGTCTGGAGGGAGATGAAGTACTGCAGTATGTGCCATCTGCTGGTGAAAAGCTGGAGG 751
HY1 cDNA   TTCCA.C.CCCAG..............................G.......C............................ 800

                  810       820       830       840       850       860       870       880
           ....|....|....|....|....|....|....|....|....|....|....|....|....|....|....|....|
KU70cDNA   ATATGACTGATCAACTGAGAAAGCGAATGATGAAAAAGCGCAAAGTCAAAACTCTCTCATTTGCAATTACAAATGATGTT 831
HY1 cDNA   ......................................................................G......... 880

                  890       900       910       920       930       940       950       960
           ....|....|....|....|....|....|....|....|....|....|....|....|....|....|....|....|
KU70cDNA   TGCATAGAGGTGAACACATATGCGTTAATCCGTCCAACTGCTCCAGGGACGATCACGTGGCTTGACTCGATCAGTAACCT 911
HY1 cDNA   ........................C............................T.......................... 960

                  970       980       990      1000      1010      1020      1030      1040
           ....|....|....|....|....|....|....|....|....|....|....|....|....|....|....|....|
KU70cDNA   TCCATTAAAGACTGAAAGGTCATTCATATGCAATGACACCGGGGCCCTTCTTCAGGCTCCCCAAGAGCGCTTCCAGCTAT 991
HY1 cDNA   .....................T.......................................................... 1040

                 1050      1060      1070      1080      1090      1100      1110      1120
           ....|....|....|....|....|....|....|....|....|....|....|....|....|....|....|....|
KU70cDNA   ACAATGATAAAGTTGTTAAATTCTCTGTTCGTGAATTGTCTGATGTGAAGAGGGTTTCAAGTCATCATCTTCGCCTTTTA 1071
HY1 cDNA   ......................T............................................C............ 1120

                 1130      1140      1150      1160      1170      1180      1190      1200
           ....|....|....|....|....|....|....|....|....|....|....|....|....|....|....|....|
KU70cDNA   GGGTTCAAGCCATTGGATTGCTTAAAAGATTATCATAACTTAAGTCCATCAACATTTATTTACCCCAGTGATGAGCAAAT 1151
HY1 cDNA   ................................................................................ 1200

                 1210      1220      1230      1240      1250      1260      1270      1280
           ....|....|....|....|....|....|....|....|....|....|....|....|....|....|....|....|
```

```
KU70cDNA  ATTCGGAAGCACTCGTGTGTTTGTTGCTTTACATAGCTCGATGCCGCGTCTTGGAAGGTTTGCACTTGCGTTTTACGGGA 1231
HY1 cDNA  ............................................T................................... 1280

                   1290      1300      1310      1320      1330      1340      1350      1360
          ....|....|....|....|....|....|....|....|....|....|....|....|....|....|....|....|
KU70cDNA  CCCCAACTCGCCCACGGCTTGTAGCCCTTGTTGCACAAGAAGAGGTTATTTCTTCGAGTGGTCAAGATGAGCCACCTGGA 1311
HY1 cDNA  ................................................................................ 1360

                   1370      1380      1390      1400      1410      1420      1430      1440
          ....|....|....|....|....|....|....|....|....|....|....|....|....|....|....|....|
KU70cDNA  ACGCACATGATCTATCTTCCATATTCGGATGATGTTAGATATCCTGAAGAGGTTCATCTGACTTCTGGGGATGCACCTCG 1391
HY1 cDNA  .T.............................................................................. 1440

                  1450      1460      1470      1480      1490      1500      1510      1520
          ....|....|....|....|....|....|....|....|....|....|....|....|....|....|....|....|
KU70cDNA  CGCTACAGATGAGCAAATAAAGAAAGCTTCGAATCTGTTGAGACGTATTGACCTGAAGCATTTCTCAGTAAGCCATTTTG 1471
HY1 cDNA  ................................................................................ 1520

                  1530      1540      1550      1560      1570      1580      1590      1600
          ....|....|....|....|....|....|....|....|....|....|....|....|....|....|....|....|
KU70cDNA  CTAACCCAGGATTGCAAAAACACTATGGGATTTTGGAGGCCTTAGCTTTAGGTGAGGATGAGATGCCGGATATGAAGGAT 1551
HY1 cDNA  .........................................................................A...... 1600

                   1610      1620      1630      1640      1650      1660      1670      1680
          ....|....|....|....|....|....|....|....|....|....|....|....|....|....|....|....|
KU70cDNA  GAGACCCTGCCCGATGAAGAAGGCTTGGCTAGGCCAGGAGTAGTTAAAGCTATAGAAGAATTTAAGGCTGCAGTGTTTGG 1631
HY1 cDNA  ................................................................................ 1680

                  1690      1700      1710      1720      1730      1740      1750      1760
          ....|....|....|....|....|....|....|....|....|....|....|....|....|....|....|....|
KU70cDNA  TGAAAATTATGACCAAGAGGAAGCTGAGGCAGCAGCAGCAAAAGGTGGGGCCTCAAAGAAGAGGAAAGCAATTGCTGATG 1711
HY1 cDNA  ................................................................................ 1760

                   1770      1780      1790      1800      1810      1820      1830      1840
          ....|....|....|....|....|....|....|....|....|....|....|....|....|....|....|....|
KU70cDNA  CAGCTTCACAGAAAAGCGCGGCCTATGATTGGGCAGACCTTGCAGATAATGGGAAGCTGAAGGACATGACGGTGATGGGT 1791
HY1 cDNA  ..............................................................................A. 1840

                  1850      1860      1870      1880      1890      1900      1910      1920
          ....|....|....|....|....|....|....|....|....|....|....|....|....|....|....|....|
KU70cDNA  TTGAAAACTTACCTGACGGCGCATGGCCTCCCAGTTTCTGGCAAGAAAGACGCCATCATCAGCAGGATCTTGACTCATCT 1871
HY1 cDNA  ................................................................................ 1920

                  1930
          ....|....|
KU70cDNA  AGGCAAGTGA 1881
HY1 cDNA  .......... 1930
```
