# Supplementary material for: Factors Affecting the Radiosensitivity of Hexaploid Wheat to γ-Irradiation: Radiosensitivity of Hexaploid Wheat (Triticum aestivum L.)
Source: PLoS One. 2016 Aug 23;11(8):e0161700. doi: 10.1371/journal.pone.0161700 (PMC4995049; doi:10.1371/journal.pone.0161700)
Supplement: S3 Fig — Taku70 encodes a 626 amino acid residues protein. The detained mRNA encoded 200 amino acid residues protein. (PDF) [file pone.0161700.s003.pdf]

```
                10        20        30        40        50        60        70        80
        ....|....|....|....|....|....|....|....|....|....|....|....|....|....|....|....|
KU70    MDLDPEGIFRDDSDEDDDNLHEREANKEMVVYLIDASPKMFTPANAAKPDEKQETHFHTIVNCITQSLKTQIIGRSRDEV 80
HY1     ...................................................N............................ 80

                90       100       110       120       130       140       150       160
        ....|....|....|....|....|....|....|....|....|....|....|....|....|....|....|....|
KU70    AICFFNTKEKKNLQELAGVYVYNVTEREQLDRPDARLIKEFSCVEDSFMNTIGSRYGITSGSRENTLYNALWVAQALLRK 160
HY1     ...................................G..............N............................. 160

               170       180       190       200       210       220       230       240
        ....|....|....|....|....|....|....|....|....|....|....|....|....|....|....|....|
KU70    GSVKTVSKRILIFTNEDDPFGGITGAAKTDMIRTTIQRAKDAQDLGLSIELLPLSRPDEDFNMSLFYADLIGLEGDEVLQ 240
HY1     ........................................VCL                                   203

               250       260       270       280       290       300       310       320
        ....|....|....|....|....|....|....|....|....|....|....|....|....|....|....|....|
KU70    YVPSAGEKLEDMTDQLRKRMMKKRKVKTLSFAITNDVCIEVNTYALIRPTAPGTITWLDSISNLPLKTERSFICNDTGAL 320
HY1                                                                           203

               330       340       350       360       370       380       390       400
        ....|....|....|....|....|....|....|....|....|....|....|....|....|....|....|....|
KU70    LQAPQERFQLYNDKVVKFSVRELSDVKRVSSHHLRLLGFKPLDCLKDYHNLSPSTFIYPSDEQIFGSTRVFVALHSSMPR 400
HY1                                                                           203

               410       420       430       440       450       460       470       480
        ....|....|....|....|....|....|....|....|....|....|....|....|....|....|....|....|
KU70    LGRFALAFYGTPTRPRLVALVAQEEVISSSGQDEPPGTHMIYLPYSDDVRYPEEVHLTSGDAPRATDEQIKKASNLLRRI 480
HY1                                                                           203

               490       500       510       520       530       540       550       560
        ....|....|....|....|....|....|....|....|....|....|....|....|....|....|....|....|
KU70    DLKHFSVSHFANPGLQKHYGILEALALGEDEMPDMKDETLPDEEGLARPGVVKAIEEFKAAVFGENYDQEEAEAAAAKGG 560
HY1                                                                           203

               570       580       590       600       610       620
        ....|....|....|....|....|....|....|....|....|....|....|....|.
KU70    ASKKRKAIADAASQKSAAYDWADLADNGKLKDMTVMGLKTYLTAHGLPVSGKKDAIISRILTHLGK 626
HY1                                                                203
```
