# Supplementary material for: Factors Affecting the Radiosensitivity of Hexaploid Wheat to γ-Irradiation: Radiosensitivity of Hexaploid Wheat (Triticum aestivum L.)
Source: PLoS One. 2016 Aug 23;11(8):e0161700. doi: 10.1371/journal.pone.0161700 (PMC4995049; doi:10.1371/journal.pone.0161700)
Supplement: S4 Fig — (PDF) [file pone.0161700.s004.pdf]

J411 mRNA sequence (total 1881bp)

>J411-1

ATGGACCTGGACCCCGAGGGCATCTTCCGCGACGACAGCGACGAGGACGACGACAAC
CTCCATGAGAGGGAGGCCAACAAGGAGATGGTCGTCTACCTCATAGACGCCTCGCCCA
GGATGTTCACCCCCGCCAACGCCGCCAAGCCAGATGAAAAGCAGGAGACACATTTCC
ATACCATAGTGAACTGCATCACGCAGTCTCTGAAGATGCAGATTATCGGGGGATCCCGT
GATGAAGTTGCAATATGCTTCTTTAACACCAAAGAAAAGAAAAATTTACAGGAGCTGG
CTGGTGTATATGTTTACAATGTCACAGAAAGAGAGCAACTTGATAGGCCTGATGCAAG
ACTGATTAAAGAATTTTCTTGTGTAGAAGATTCTTTTATGAATAACATTGGAAGCCGGTA
TGGAATAACCTCTGGATCTCGAGAGAATACCCTGTACAATGCTCTTTGGGTTGCACAGG
CACTGCTGCGTAAAGGATCTGTGAAGACTGTGAGTAAGAGAATCCTCATATTCACCAAT
GAGGACGATCCCTTTGGTGGTATTACAGGAGCAGCAAAGACTGATATGATTAGGACCA
CAATTCAACGTGCAAAAGATGCACAAGATCTGGGCCTGTCTATCGAACTTCTTCCATTG
AGTAGGCCTGATGAGGATTTCAACATGTCCCTGTTTTATGCAGATTTGATTGGTCTGGA
GGGAGATGAAGTAGTGCAGTACGTGCCATCTGCTGGTGAAAAGCTGGAGGATATGACC
GATCAACTGAAAAAGCGAATGATGAAAAAGCGCAAAGTCGAAACTCTCTCATTTGCAA
TTACAAATGATGTTTGCATAGAGGTGAACACATATGCGCTAATCCGTCCAACTGCTCCA
GGGACGATTACGTGGCTTGACTCGATCAGTAACCTTCCATTAAAGACTGAAAGGTCTTT
CATATGCAATGATACTGGGGCCCTTCTTCAGGCTCCTCAAGAGCGCTTCCAGCTATACA
ATGATAAAGTTGTGAAATTTTCTGTTCGTGAATTGTCTGATGTGAAGAGGGTTTCAAGT
CATCATCTTCGCCTTTTGGGGTTCAAGCCATTGGATTGCTTAAAAGATTATCATAACTTA
AGTCCATCAACATTTATTTACCCCAGTGATGAGCAAATATTCGGAAGCACTCGTGTTTTC
GTTGCTTTACATAGCTCGATGCTGCGTCTTGGACGGTTTGCACTTGCATTTTACGGGAC
CCCAACTCGCCCACGGCTTGTAGCCCTTGTTGCACAAGAAGAGGTTATTTCTTCGAGT
GGTCAAGATGAGCCACCTGGAATGCACATGATCTATCTTCCATACTCGGATGATGTTAG
ATATCCTGAAGAGGTTCATCTGACTTCTGGGGATGCACCTCGCGCTACAGATGAGCAAA
TAAAGAAAGCTTCGAATCTGTTGAGACGTATTGACCTGAAGCATTTCTCAGTAAGCCAT
TTTGCTAACCCAGGATTGCAAAAACACCATGGGATTTTGGAGGCCTTAGCTTTAGGTGA
GGATGAGATGCCGGATATAAAGGATGAGACCCTGCCCGATGAAGAAGGCTTGGCTAGG
CCAGGAGTAGTTAAAGCTATAGAAGAATTTAAGGCTGCAGTGTTTGGTGAAAACTATG
ACCAAGAGGAAGCTGAGGCAGCAGCAGCAAAAGGTGGGGCCTCAAAGAAGAGGAA
AGCAATTGCTGATGCAGCTTCACAGAAAGGCGCGGCTTATGACTGGGCAGACCTTGCT
GATAATGGGAAGCTGAAGGACATGACGGTGATGGATTTGAAAACTTACTTGACGGCGC
ATGGACTCCCAGTTTCTGGTAAGAAAGACGCCATCATCAGCAGGATCTTGACTCATCTA
GGCAAGTGA

>J411-14

ATGGACCTGGACCCCGAGGGCATCTTCCGCGACGACAGCGACGAGGACGACGACAAC
CTCCATGAGAGGGAGGCCAACAAGGAGATGGTCGTCTACCTCATAGACGCCTCGCCCA
GGATGTTCACCCCCGCCAACGCCGCCAAGCCAGATGAAAAGCAGGAGACACATTTCC
ATACCATAGTGAACTGCATCACGCAGTCTCTGAAGATGCAGATTATCGGGAGATCCCGT
GATGAAGTTGCAATATGCTTCTTTAACACCAAAGAAAAGAAAAATTTACAGGAGCTGG
CTGGTGTATATGTTTACAATGTCACAGAAAGAGAGCAACTTGATAGGCCTGATGCAAG
ACTGATTAAAGAATTTTCTTGTGTAGAAGATTCTTTTATGAATAACATTGGAAGCCGGTA

TGGAATAACCTCTGGATCTCGAGAGAATACCCTGTACAATGCTCTTTGGGTTGCACAGG
CACTGCTGCGTAAAGGATCTGTGAAGACTGTGAGTAAGAGAATCCTCATATTCACCAAT
GAGGACGATCCTTTTGGTGGTATTACAGGAGCAGCAAAGACTGATATGATTAGGACCA
CAATTCAACGTGCAAAAGATGCACAAGATCTGGGCCTGTCTATCGAACTTCTTCCATTG
AGTAGGCCTGATGAGGATTTCAACATGTCCCTGTTTTATGCAGATTTGATTGGTCTGGA
GGGAGATGAAGTAGTGCAGTACGTGCCATCTGCTGGTGAAAAGCTGGAGGATATGACC
GATCAACTGAAAAAGCGAATGATGAAAAAGCGCAAAGTCAAAACTCTCTCATTTGCAA
TTACAAATGATGTTTGCATAGAGGTGGACACATATGCGCTAATCCGTCCAACTGCTCCA
GGGACGATTACGTGGCTTGACTCGATCAGTAACCTTCCATTAAAGACTGAAAGGTCTTT
CATATGCAATGATACTGGGGCCCTTCTTCAGGCTCCTCAAGAGCGCTTCCAGCTATACA
ATGATAAAGTTGTGAAATTTTCTGTTCGTGAGTTGTCTGATGTGAAGAGGGTTTCAAGC
CATCATCTTCGCCTTTTAGGGTTCAAGCCATTGGATTGCTTAAAAGATTATCATAACTTA
AGTCCATCAACATTTATTTACCCCAGTGATGAGCAAATATTCGGAAGCACTCGTGTTTTC
GTTGCTTTACATAGCTCGATGCTGCGTCTTGGACGGTTTGCACTTGCATTTTACGGGAC
CCCAACTCGCCTACGGCTTGTAGCCCTTGTTGCACAAGAAGAGGTTATTTCTTCGAGTG
GTCAAGATGAGCCACCTGGAATGCACATGATCTATCTTCCATACTCGGATGATGTTAGAT
ATCCTGAAGAGGTTCATCTGACTTCTGGGGATGCACCTCGCGCTACAGATGAGCAAAT
AAAGAAAGCTTCGAATCTGTTGAGACGTATTGACCTGAAGCATTTCTCAGTAAGCCATT
TTGCTAACCCAGGATTGCAAAAACACTATGGGATTTTGGAGGCCTTAGCTTTAGGTGAG
GATGAGATGCCGGATATAAAGGATGAGACCCTGCCCGATGAAGAAGGCTTGGCCAGGC
CAGGAGTAGTTAAAGCTATAGAAGAATTTAAGGCTGCAGTGTTTGGTGAAAACTATGA
CCAAGAGGAAGCTGAGGCAGCAGCAGCAAAAGGTGGGGCCTCAAAGAAGAGGAAA
GCAGTTGCTGATGCAGCTTCACAGAAAAGCGCGGCTTATGACTGGGCAGACCTTGCTG
ATAATGGGAAGCTGAAGGACATGACGGTGATGGATTTAAAAACTTACTTGACGGCGCA
TGGACTCCCAGTTTCTGGTAAGAAAGACGCCATCATCAGCAGGATCTTGACTCATCTAG
GCAAGTGA

>J411-24

ATGGACCTGGACCCCGAGGGCATCTTCCGCGACGACAGCGACGAGGACGACGACAAC
CTCCATGAGAGGGAGGCCAACAAGGAGATGGTCGTCTACCTCATAGACGCCTCGCCCA
GGATGTTCACCCCCGCCAACGCCGCCAAGCCAGATGAAAAGCAGGAGACTCATTTCCA
TACCATAGTGAACTGCATCACGCAGTCTCTGAAGATGCAGATTATCGGGAGATCCCGTG
ATGAAGTTGCAATATGCTTCTTTAACACCAAAGAAAAGAAAAATTTACAGGAGCTGGC
TGGTGTATATGTTTACAATGTCACAGAAAGAGAGCAACTTGATAGGCCTGATGCAAGA
CTGATTAAAGAATTTTCCTGTGTAGAAGATTCTTTTATGAATAACATTGGAAGCCGGTAT
GGAATAACCTCTGGATCTCGAGAGAATACCCTGTACAATGCTCTTTGGGTTGCACAGGC
ACTGCTGCGTAAAGGATCTGTGAAGACTGTGAGTAAGAGAATCCTCATATTCACCAATG
AGGACGATCCTTTTGGTGGTATTACAGGAGCAGCAAAGACTGATATGATTAGGACCAC
AATTCAACGTGCAAAAGATGCACAAGATCTGGGCCTGTCTATCGAACTTCTTCCATTGA
GTAGGCCTGATGAGGATTTCAACATGTCCCTGTTTTATGCAGATTTGATTGGTCTGGAG
GGAGATGAAGTAGTGCAGTACGTGCCATCTGCTGGTGAAAAGCTGGAGGATATGACCG
ATCAACTGAGAAAGCGAATGATGAAAAAGCGCAAAGTCAAAACTCTCTCATTTGCAAT
TACAAATGATGTTTGCATAGAGGTGAACACATATGCGCTAATCCGTCCAACTGCTCCAG
GGACGATTACGTGGCTTGACTCGATCAGTAACCTTCCATTAAAGACTGAAAGGTCTTTC
ATATGCAATGATACTGGGGCCCTTCTTCAGGCTCCTCAAGAGCGCTTCCAGCTATACAA

TGATAAAGTTGTGAAATTTTCTGTTCGTGAATTGTCTGATGTGAAGAGGGTTTCAAGTC
ATCATCTTCGCCTTTTAGGGTTCAAGCCATTGGATTGCTTAAAAGATTATCATAACTTAA
GTCCATCAACATTTATTTACCCCAGTGATGAGCAAATATTCGGAAGCACTCGTGTTTTCG
TTGCTTTACATAGCTCGATGCTGCGTCTTGGACGGTTTGCACTTGCATTTTACGGGACC
CCAACTCGCCCACGGCTTGTAGCCCTTGTTGCACAAGAAGAGGTTATTTCTTCGAGTG
GTCAAGATGAGCCACCTGGAATGCACATGATCTATCTTCCGTACTCGGATGATGTTAGAT
ATCCTGAAGAGGTTCATCTGACTTCTGGGGATGCACCTCGCGCTACAGATGAGCAAAT
AAAGAAAGCTTCGAATCTGTTGAGACGTATTGACCTGAAGCATTTCTCAGTAAGCCATT
TTGCTAACCCAGGATTGCAAAAACACTATGGGATTTTGGAGGCCTTAGCTTTAGGTGAG
GATGAGATGCCGGATATAAAGGATGAGACCCTGCCCGATGAAGAAGGCTTGGCTAGGC
CAGGAGTAGTTAAAGCTATAGAAGAATTTAAGGCTGCAGTGTTTGGTGAAAACTATGA
CCAAGAGGAAGCTGAGGCAGCAGCAGCAAAAGGTGGGGCCTCAAAGAAGAGGAAA
GCAATTGCTGATGCAGCTTCACAGAAAAGCGCGGCTTATGACTGGGCAGACCTTGCTG
ATAATGGGAAGCAGAAGGACATGACGGTGATGGATTTGAAAACTTACTTGACGGCGCA
TGGACTCCCAGTTTCTGGTAAGAAAGACGCCATCATCAGCAGGATCTTGACTCATCTAG
GCAAGTGA

>J411-15

ATGGACCTGGACCCCGAGGGCATCTTCCGCGACGACAGCGACGAGGACGACGACAAC
CTCCATGAGAGGGAGGCCAACAAGGAGATGGTCGTCTACCTCATAGACGCCTCGCCCA
GGATGTTCACCCCCGCCAACGCCGCCAAGCCAGATGAAAAGCAGGAGACACATTTCC
ATACCATAGTGAACTGCATCACGCAGTCTCTGAAGATGCAGATTATCGGGAGATCCCGT
GATGAAGTTGCGATATGCTTCTTTAACACCAAAGAAAAGAAAAATTTACAGGAGCTGG
CTGGTGTATATGTTTACAATGTCACAGAAAGAGAGCAACTTGATAGGCCTGATGCAAG
ACTGATTAAAGAATTTTCTTGTGTAGAAGATTCTTTTATGAATAACATTGGAAGCCGGTA
TGGAATAACCTCTGGATCTCGAGAGAATACCCTGTACAATGCTCTTTGGGTTGCACAGG
CACTGCTGCGTAAAGGATCTGTGAAGACTGTGAGTAAGAGAATCCTCATATTCACCAAT
GAGGACGATCCTTTTGGTGGTATTACAGGAGCAGCAAAGACTGATATGATTAGGACCA
CAATTCAACGTGCAAAAGATGCACAAGATCTGGGCCTGTCTATCGAACTTCTTCCATTG
AGTAGGCCTGATGAGGATTTCAACATGTCCCTGTTTTATGCAGATTTGATTGGTCTGGA
GGGAGATGAAGTAGTGCAGTACGTGCCATCTGCTGGTGAAAAGCTGGAGGATATGACC
GACCAACTGAAAAAGCGAATGATGAAAAAGCGCAAAGTCAAAACTCTCTCATTTGCA
ATTACAAATGATGTTTGCATAGAGGTGAACACATATGCGCTAATCCGTCCAACTGCTCC
AGGGACGATTACGTGGCTTGACTCGATCAGTAACCTTCCATTAAAGACTGAAAGGTCT
TTCATATGCAATGATACTGGGGCCCTTCTTCAGGCTCCTCAAGAGCGCTTCCAGCTATA
CAATGATAAAGTTGTGAAATTTTCTGTTCGTGAATTGTCTGATGTGAAGAGGGTTTCAA
GTCATCATCTTCGCCTTTTAGGGTTCAAGCCATTGGATTGCTTAAAAGATTATCATAACT
TAAGTCCATCAACATTTATTTACCCCAGTGATGAGCAAATATTCGGAGGCACTCGTGTTT
TCGTTGCTTTACATAGCTCGATGCTGCGTCTTGGACGGTTTGCACTTGCATTTTACGGG
ACCCCAACTCGCCCACGGCTTGTAGCCCTTGTTGCACAAGAAGAGGTTATTTCTTCGA
GCGGTCAAGATGAGCCACCTGGAATGCACATGATCTATCTTCCATACTCGGATGATGTT
AGATATCCTGAAGAGGTTCATCTGACTTCTGGGGATGCACCTCGCGCTACAGATGAGC
AAATAAAGAAAGCTTCGAATCTGTTGAGACGTATTGACCTGAAGCATTTCTCAGTAAG
CCATTTTGCTAACCCAGGATTGCAAAAACACTATGGGATTTTGGAGGCCTTAGCTTTAG
GTGAGGATGAGATGCCGGATATAAAGGATGAGACCCTGCCCGATGAAGAAGGCTTGGC

TAGGCCAGGAGTAGTTAAAGCTATAGAAGAATTTAAGGCTGCAGTGTTTGGTGAAAAC
TATGACCAAGAGGAAGCTGAGGCAGCAGCAGCAAAAGGTGGGGCCTCAAAGAAGAG
GAAAGCAATTGCTGATGCAGCTTCACAGAAAAGCGCGGCTTATGACTGGGCAGACCTT
GCTGATAATGGGAAGCTGAAGGACATGACGGTGATGGATTTGAAAACTTACTTGACGG
CGCATGGACTCCCAGTTTCTGGTAAGAAAGACGCCATCATCAGCAGGATCTTGACTCAT
CTAGGCAAGTGA

>J411-29

TGGACCTGGACCCCGAGGGCATCTTCCGCGACGACAGCGACGAGGACGACGACAACC
TCCATGAGAGGGAGGCCAACAAGGAGATGGTCGTCTACCTCATAGACGCCTCGCCCAG
GATGTTCACCCCCGCCAACGCCGCCAAGCCAGATGAAAAGCAGGAGACACATTTCCAT
ACCATAGTGAACTGCATCACGCAGTCTCTGAAGATGCAGATTATCGGGAGATCCCGTGA
TGAAGTTGCAATATGCTTCTTTAACACCAAAGAAAAGAAAAATTTACAGGAGCTGGCT
GGTGTATATGTTTACAATGTCACAGAAAGAGAGCAACTTGATAGGCCTGATGCAAGAC
TGATTAAAGAATTTTCTTGTGTAGAAGATTCTTTTATGAATAACATTGGAAGCCGGTATG
GAATAACCTCTGGATCTCGAGAGAATACCCTGTACAATGCTCTTTGGGTTGCACAGGCA
CTGCTGCGTAAAGGATCTGTGAAGACTGTGAGTAAGAGAATCCTCATATTCACCAATGA
GGACGATCCTTTTGGTGGTATTACAGGAGCAGCAAAGACTGATATGATTAGGACCACAA
TTCAACGTGCAAAAGATGCACAAGATCTGGGCCTGTCTATCGAACTTCTTCCATTGAGT
AGGCCTGATGAGGATTTCAACATGTCCCTGTTTTATGCAGATTTGATTGGTCTGGAGGG
AGATGAAGTAGTGCAGTACGTGCCATCTGCTGGTGAAAAGCTGGAGGATATGACCGAT
CAACTGAGAAAGCGAATGACGAAAAAGCGCAAAGTCAAAACTCTCTCATTTGCAATT
ACAAATGATGTTTGCATAGAGGTGAACACATATGCGCTAATCCGTCCAACTGCTCCAGG
GACGATTACGTGGCTTGACTCGATCAGTAACCTTCCATTAAAGACTGAAAGGTCTTTCA
TATGCAATGATACTGGGGCCCTTCTTCAGGCTCCTCAAGAGCGCTTCCAGCTACACAAT
GATAAAGTTGTGAAATTTTCTGTTCGTGAATTGTCTGATGTGAAGAGGGTTTCAAGTCA
TCATCTTCGCCTTTTAGGGTTCAAGCCATTGGATTGCTTAGAAGAGTATCATAACTTAAG
TCCATCAACATTTATTTACCCCAGTGATGAGCAAATATTCGGAAGCACTCGTGTTTTCGT
TGCTTTACATAGCTCGATGCTGCGTCTTGGACGGTTTGCACTTGCATTTTACGGGACCC
CAACTCGCCCACGGCTTGTAGCCCTTGTTGCACAAGAAGAGGTTATTTCTTCGAGTGG
TCAAGATGAGCCACCTGGAATGCACATGATCTATCTTCCATACTCGGATGATGTTAGATA
TCCTGAAGAGGTTCATCTGACTTCTGGGGATGCACCTCGCGCTACAGATGAGCAAATA
AAGAAAGCTTCGAATCTGTTGAGACGTATTGACCTGAAGCATTTCTCAGTAAGCCATTT
TGCTAACCCAGGATTGCAAAAACACTATGGGATTTTGGAGGCCTTAGCTTTAGGTGAG
GATGAGATGCCGGATATAAAGGATGAGACCCTGCCCGATGAAGAAGGCTTGGCTAGGC
CAGGAGTAGTTAAAGCTATAGAAGAATTTAAGGCTGCAGTGTTTGGTGAAAACTATGA
CCAAGAGGAAGCTGAGGCAGCAGCAGCGAAAGGTGGGGCCTCAAAGAAGAGGAAA
GCAATTGCTGATGCAGCTTCACAGAAAAGCGCGGCTTATGACTGGGCAGACCTTGCTG
ATAATGGGAAGCTGAAGGACATGACGGTGATGGATTTGAAAACTTACTTGACGGCGCA
TGGACTCCCAGTTTCTGGTAAGAAAGACGCCATCATCAGCAGGATCTTGACTCATCTAG
GCAAGTGA

>J411-39

ATGGACCTGGACCCCGAGGGCATCTTCCGCGACGACAGCGACGAGGACGACGACAAC
CTCCATGAGAGGGAGGCCAACAAGGAGATGGTCGTCTACCTCATAGACGCCTCGCCCA
GGATGTTCACCCCCGCCAACGCCGCCAAGCCAGATGAAAAGCAGGAGACACATTTCC

ATACCATAGTGAACTGCATCACGCAGTCTCTGAAGATGCAGATTATCGGGAGATCCCGT
GATGAAGTTGCAATATGCTTCTTTAACACCAAAGAAAAGAAAAATTTACAGGAGCTGG
CTGGTGTATATGTTTACAATGTCACAGAAAGAGAGCAACTTGATAGGCCTGATGCAAG
ACTGATTAAAGAATTTTCTTGTGTAGAAGATTCTTTTATGAATAACATTGGAAGCCGGTA
TGGAATAACCTCTGGATCTCGAGAGAATACCCTGTACAATGCTCTTTGGGTTGCACAGG
CACTGCTGCGTAAAGGATCTGTGAAGACTGTGAGTAAGAGAATCCTCATATTCACCAAT
GAGGACGATCCTTTTGGTGGTATTACAGGAGCAGCAAAGACTGATATGATTAGGACCA
CAATTCAACGTGCAAAAGATGCACAAGATCTGGGCCTGTCTATCGAACTTCTTCCATTG
AGTAGGCCTGATGAGGATTTCAACATGTCCCTGTTTTATGCAGATTTGATTGGTCTGGA
GGGAGATGAAGTAGTGCAGTACGTGCCATCTGCTGGTGAAAAGCTGGAGGATATGACC
GATCAACTGAGAAAGCGAATGATGAAAAAGCGCAAAGTCAAAACTCTCTCATTTGCAA
TTACAAATGATGTTTGCATAGAGGTGAACACATATGCGCTAATCCGTCCAACTGCTCCA
GGGACGATTACGTGGCTTGACTCGATCAGTAACCTTCCATTAAAGACTGAAAGGTCTTT
CATATGCAATGATACTGGGGCCCTTCTTCAGGCTCCTCAAGAGCGCTTCCAGCTATACA
ATGATAAAGTTGTGAAATTTTCTGTTCGTGAATTGTCTGATGTGAAGAGGGTTTCAAGT
CATCATCTTCGCCTTTTAGGGTTCAAGCCATTGGATTGCTTAAAAGATTATCATAACTTA
AGTCCATCAACATTTATTTACCCCAGTGATGAGCAAATATTCGGAAGCACTCGTGTTTTC
GTTGCTTTACATAGCTCGATGCTGCGTCTTGGACGGTTTGCACTTGCATTTTACGGGAC
CCCAACTCGCCCACGGCTTGTAGCCCTTGTTGCACAAGAAGAGGTTATTTCTTCGAGT
GGTCAAGATGAGCCACCTGGAATGCACATGATCTATCTTCCATACTCGGATGATGTTAG
ATATCCTGAAGAGGTTCATCTGACTTCTGGGGATGCACCTCGCGCTACAGATGAGCAAA
TAAAGAAAGCTTCGAATCTGTTGAGACGTATTGACCTGAAGCATTTCTCAGTAAGCCAT
TTTGCTAACCCAGGATTGCAAAAACACTATGGGATTTTGGAGGCCTTAGCTTTAGGTGA
GGATGAGATGCCGGATATAAAGGATGAGACCCTGCCCGATGAAGAAGGCTTGGCTAGG
CCAGGAGTAGTTAAAGCTATAGAAGAATTTAAGGCTGCAGTGTTTGGTGAAAACTATG
ACCAAGAGGAAGCTGAGGCAGCAGCAGCAAAAGGTGGGGCCTCAAAGAAGAGGAA
AGCAATTGCTGATGCAGCTTCACAGAAAAGCGCGGCTTATGACTGGGCAGACCTTGCT
GATAATGGGAAGCTGAAGGACATGACGGTGATGGATTTGAAAACTTACTTGACGGCGC
ATGGACTCCCAGTTTCTGGTAAGAAAGACGCCATCATCAGCAGGATCTTGACTCATCTA
GGCAAGTGA

>J411-6

ATGGACCTGGACCCCGAGGGCATCTTCCGCGACGACAGCGACGAGGACGACGACAAC
CTCCATGAGAGGGAGGCCAACAAGGAGATGGTCGTCTACCTCATAGACGCCTCGCCCA
AGATGTTCACACCCGCCAACGCCGCCAAGCCAGATGAAAAGCAGGAGACACATTTCC
ATACCATAGTGAACTGCATCACGCAGTCTCTGAAGACGCAGATTATCGGGAGATCCCGT
GATGAAGTTGCAATATGCTTCTTTAACACCAAAGAAAAGAAAAATTTACAGGAGCTGG
CTGGTGTATATGTTTACAATGTCACAGAAAGAGAGCAACTTGATAGACCTGATGCAAG
ACTGATTAAAGAATTTTCTTGTGTAGAAGATTCTTTTATGAATACCATTGGAAGCCGGTA
TGGAATAACCTCTGGATCTAGAGAGAATACCCTGTACAATGCTCTTTGGGTTGCACAGG
CACTGCTGCGTAAAGGATCTGTGAAGACTGTGAGTAAGAGAATCCTCATATTCACCAAT
GAGGATGATCCTTTTGGTGGTATTACAGGAGCAGCAAAGACTGATATGATTAGGACCAC
AATTCAACGTGCAAAAGATGCACAAGATCTGGGCCTGTCTATCGAACTTCTTCCATTGA
GTAGGCCTGATGAGGATTTCAACATGTCCCTGTTTTATGCAGATTTGATTGGTCTGGAG
GGAGATGAAGTACTGCAGTATGTGCCATCTGCTGGTGAAAAGCTGGAGGATATGACTG

ATCAACGGAGAAAGCGAATGATGAAAAAGCGCAAAGTCAAAACTCTCTCATTTGCAAT
TACAAATGATGTTTGCATAGAGGTGAACACATATGCGTTAATCCGTCCAACTGCTCCAG
GGACGATCATGTGGCTTGACTCGATCAGTAACCTTCCATTAAAGACTGAAAGGTCATTC
ATATGCAATGACACCGGGGCCCTTCTTCAGGCTCCCAAGAGCGCTTCCAGCTATACAA
TGATAAAGTTGTTAAATTTTCTGTTCGTGAATTGTCTGATGTGAAGAGGGTTTCAAGTC
ATCATCTTCGCCTTTTAGGGTTCAAGCCATTGGATTGCTTAAAAGATTATCATAACTTAA
GTCCATCAACACTTATTTACCCCAGTGATGAGCAAATATTCGGAAGCACTCGTGTGTTT
GTTGCTTTACATAGCTCGATGCTGCGTCTTGGAAGGTTTGCACTTGCGTTTTACGGGAC
CCCAACTCGCCCACGGCTTGTAGCCCTTGTTGCACAAGAAGAGGTTATTTCTTCGAGT
GGTCAAGATGAGCCACCTGGAATGCACATGATCTATCTTCCATATTCGGATGATGTTAGA
TATCCTGAAGAGGTTCATCTGACTTCTGGGGATGCACCTCGCGCTACAGATGAGCAAAT
AAAGAAAGCTTCGAATCTGTTGAGACGTATTGACCTGAAGCATTTCTCAGTAAGCCATT
TTGCTAACCCAGGATTGCAAAAACACTATGGGATTTTGGAGGCCTTAGCTATAGGTGAG
GATGAGATGCCGGATATAAAGGATGAGACCCTGCCCGATGAAGAAGGCTTGGCTAGGC
CAGGAGTAGTTAAAGCTATAGAAGAATTTAAGGCTGCAGTGTTTGGTGAAAACTATGA
CCAAGAGGAAGCTGAGGCAGCAACAGCAAAAGGTGGGGCCTCAAAGAAGAGGAAA
GCAATTGCTGATGCAGCTTCACAGAAAAGCGCGGCTTATGACTGGGCAGACCTTGCTG
ATAATGGGAAGCTGAAGGACATGACGGTGATGGATTTGAAAACTTACTTGACGGCGCA
TGGACTCCCAGTTTCTGGTAAGAAAGACGCCATCATCAGCAGGATCTTGACTCATCTAG
GCAAGTGA
>J411-34
ATGGACCTGGACCCCGAGGGCATCTTCCGCGACGACAGCGACGAGGACGACGACAAC
CTCCATGAGAGGGAGGCCAACAAGGAGATGGTCGTCTACCTCATAGACGCCTCGCCCA
AGATGTTCACACCCGCCAACGCCGCCAAGCCAGATGAAAAGCAGGAGACACATTTCC
ATACCATAGTGAACTGCATCACGCAGTCTCTGAAGACGCAGATTATCGGGAGATCCCGT
GATGAAGTTGCAATATGCTTCTTTAACACCAAAGAAAAGAAAAATTTACAGGAGCTGG
CTGGTGTATATGTTTACAATGTCACAGAAAGAGAGCAACTTGATAGACCTGATGCAAG
ACTGATTAAAGAATTTTCTTGTGTAGAAGATTCTTTTATGAATACCATTGGAAGCCGGTA
TGGAATAACCTCTGGATCTAGAGAGAATACCCTGTACAATGCTCTTTGGGTTGCACAGG
CACTGCTGCGTAAAGGATCTGTGAAGACTGTGAGTAAGAGAATCCTCATATTCACCAAT
GAGGACGATCCTTTTGGTGGTATTACAGGAGCAGCAAAGACTGATATGATTAGGACCA
CAATTCAACGTGCAAAAGATGCACAAGATCTGGGCCTGTCTATCGAACTTCTTCCATTG
AGTAGGCCTGATGAGGATTTCAACATGTCCCTGTTTTATGCAGATTTGATTGGTCTGGA
GGGAGATGAAGTAGTGCAGTACGTGCCATCTGCTGGTGAAAAGCTGGAGGATATGACC
GATCAACTGAGAAAGCGAATGACGAAAAAGCGCAAAGTCAAAACTCTCTCATTTGCA
ATTACAAATGATGTTTGCATAGAGGTGAACACATATGCGCTAATCCGTCCAACTGCTCC
AGGGACGATTACGTGGCTTGACTCGATCAGTAACCTTCCATTAAAGACTGAAAGGTCT
TTCATATGCAATGATACTGGGGCCCTTCTTCAGGCTCCTCAAGAGCGCTTCCAGCTATA
CAACGATAAAGTTGTTAAATTTTCTGTTCGTGAATTGTCTGATGTGAAGAGGGTTTCAA
GTCATCATCTTCGCCTTTTAGGGTTCAAGCCATTGGATTGCTTAAAAGATTATCATAACT
TAAGTCCATCAACATTTATTTACCCCAGTGATGAGCAAATATTCGGAAGCACTCGTGTG
TTTGTTGCTTTACATAGCTCGATGCTGCGTCTTGGAAGGTTTGCACTTGCGTTTTACGG
GACCCCAACTCGCCCACGGCTTGTAGCCCTTGTTGCACAAGAAGAGGTTATTTCTTCG
AGTGGTCAAGATGAGCCACCTGGAATGCACATGATCTATCTTCCATATTCGGATGATGTT

AGATATCCTGAAGAGGTTCATCTGACTTCTGGGGATGCACCTCGCGCTACAGATGAGC
AAATAAAGAAAGCTTCGAATCTGTTGAGACGTATTGACCTGAAGCATTTCTCAGTAAG
CCATTTTGCTAACCCAGGATTGCAAAAACACTATGGGATTTTGGAGGCCTTAGCTTTAG
GTGAGGATGAGATGCCGGATATAAAGGATGAGACCCTGCCCGATGAAGAAGGCTTGGC
TAGGCCAGGAGTAGTTAAAGCTATAGAAGAATTTAAGGCTGCAGTGTTTGGTGAAAATT
ATGACCAAGAGGAAGCTGAGGCAGCAGCAGCAAAAGGTGGGGCCTCAAAGAAGAGG
AAAGCAATTGCTGATGCAGCTTCACAGAAAAGCGCGGCCTATGATTGGGCAGACCTTG
CAGATAATGGGAAGCTGAAGGACATGACGGTGATGGATTTGAAAACTTACCTGACGGC
GCATGGCCTCCCAGTTTCTGGCAAGAAAGACGCCACCATCAGCAGGATCTTGACTCAT
CTAGGCAAGTGA
>J411-17
ATGGACCTGGACCCCGAGGGCATCTTCCGCGACGACAGCGACGAGGACGACGACAAC
CTCCATGAGAGGGAGGCCAACAAGGAGATGGTCGTCTACCTCATAGACGCCTCGCCCA
GGATGTTCACCCCCGCCAACGCCGCCAAGCCAGATGAAAAGCAGGAGACACATTTCC
ATACCATAGTGAACTGCATCACGCAGTCTCTGAAGATGCAGATTATCGGGAGATCCCGT
GATGAAGTTGCAATATGCTTCTTTAACACCAAAGAAAAGAAAAATTTACAGGAGCTGG
CTGGTGTATATGTTTACAATGTCACAGAAAGAGAGCAACTTGATAGGCCTGATGCAAG
ACTGATTAAAGAATTTTCTTGTGTAGAAGATTCTTTTATGAATAACATTGGAAGCCGGTA
TGGAATAACCTCTGGATCTCGAGAGAATACCCTGTACAATGCTCTTTGGGTTGCACAGG
CACTGCTGCGTAAAGGATCTGTGAAGACTGTGAGTAAGAGAATCCTCATATTCACCAAT
GAGGACGATCCTTTTGGTGGTATTACAGGAGCAGCAAAGACTGATATGATCAGGACCA
CAATTCAACGTGCAAAAGATGCACAAGATCTGGGCCTGTCTATCGAACTTCTTCCATTG
AGTAGGCCTGATGAGGATTTCAACATGTCCCTGTTTTATGCAGATTTGATTGGTCTGGA
GGGAGATGAAGTAGTGCAGTACGTGCCATCTGCTGGTGAAAAGCTGGAGGATATGACC
GATCAACTGAAAAAGCGAATGATGAAAAAGCGCAAAGTCAAAACTCTCTCATTTGCAA
TTACAAATGATGTTTGCATAGAGGTGAACACATATGCGCTAATCCGTCCAACTGCTCCA
GGGACGATTACGTGGCTTGACTCGATCAGTAACCTTCCATTAAAGACTGAAAGGTCTTA
CATATGCAATGATACTGGGGCCCTTCTTCAGGCTCCTCAAGAGCGCTTCCAGCTATACA
ATGATAAAGTTGCGAAATTTTCTGTTCGTGAATTGTCTGATGTGAAGAGGGTTTCAAGT
CATCATCTTCGCCTTTTAGGGTTCAAGCCATTGGATTGCTTAAAAGATTATCATAACTTA
AGTCCATCAACATTTATTTACCCCAGTGATGAGCAAATATTCGGAAGCACTCGTGTTTTC
GTTGCTTTACATAGCTCGATGCTGCGTCTTGGACGGTTTGCACTTGCATTTTACGGGAC
CCCAACTCGCCCACGGCTTGTAGCCCTTGTTGCACAAGAAGAGGTTATTTCTTCGAGT
GGTCAAGATGAGCCACCTGGAATGCACATGATCTATCTTCCATACTCGGATGATGTTAG
ATATCCTGAAGAGGTTCATCTGACTTCTGGGGATGCACCTCGCGCTACAGATGAGCAAA
TAAAGAAAGCTTCGAATCTGTTGAGACGTATTGACCTGAAGCATTTCTCAGTAAGCCAT
TTTGCTAACCCAGGATTGCAAAAACACTATGGGATTTTGGAGGCCTTAGCTTTAGGTGA
GGATGAGATGCCGGATATAAAGGATGAGACCCTGCCCGATGAAGAAGGCTTGGTTAGG
CCAGGAGTAGTTAAAGCTATAGAAGAATTTAAGGCTGCAGTGTTTGGTGAAAACTATG
ACCAAGAGGAAGCTGAGGCAGCAGCAGCAAAAGGTGGGGCCTCAAAGAAGAGGAA
AGCATTTGCTGATGCAGCTTCACAGAAAAGCGCGGCTTATGACTGGGCAGACCTTGCT
GATAATGGGAAGCTGAAGGACATGACGGTGATGGATTTGAAAACTTACTTGACGGCGC
ATGGACTCCCAGTTTCTGGTAAGAAAGACGCCATCATCAGCAGGATCTTGACTCATCTA
GGCAAGTGA

>J411-9
ATGGACCTGGACCCCGAGGGCATCTTCCGCGACGACAGCGACGAGGACGACGACAAC
CTCCATGAGAGGGAGGCCAACAAGGAGATGGTCGTCTACCTCATAGACGCCTCGCCCA
GGATGTTCACCCCCGCCAACGCCGCCAAGCCAGATGAAAAGCAGGAGACACATTTCC
ATACCATAGTGAACTGCATCACGCAGTCTCTGAAGATGCAGATTATCGGGAGATCCCGT
GATGAAGTTGCAATATGCTTCTTTAACACCAAAGAAAAGAAAAATTTACAGGAGCTGG
CTGGTGTATATGTTTACAATGTCACAGAAAGAGAGCAACTTGATAGGTCTGATGCAAGA
CTGATTAAAGAATTTTCTTGTGTAGAAGATTCTTTTATGAATAACATTGGAAGCCGGTAT
GGAATAACCTCTGGATCTCGAGAGAATACCCTGTACAATGCTCTTTGGGTTGCACAGGC
ACTGCTGCGTAAAGGATCTGTGAAGACTGTGAGTAAGAGAATCCTCATATTCACCAATG
AGGACGATCCTTTTGGTGGTATTACAGGAGCAGCAAAGACTGATATGATTAGGACCAC
AATTCAACGTGCAAAAGATGCACAAGATCTGGGCCTGTCTATCGAACTTCTTCCATTGA
GTAGGCCTGATGAGGATTTCAACATGTCCCTGTTTTATGCAGATTTGATTGGTCTGGAG
GGAGATGAAGTAGTGCAGTACGTGCCATCTGCTGGTGAAAAGCTGGAGGATATGACCG
ATCAACTGAGGAAGCGAATGATGAAAAAGCGCAAAGTCAAAACTCTCTCATTTGCAAT
TACAAATGATGTTTGCATAGAGGTGAACACATACGCGCTAATCCGTCCAACTGCTCCAG
GGACGATTACGTGGCTTGACTCGATCAGTAACCTTCCATTAAAGACTGAAAGGTCTTTC
ATATGCAATGATACTGGGGCCCTTCTTCAGGCTCCTCAAGAGCGCTTCCAGCTATACAA
TGATAAAGTTGTGAAATTTTCTGTTCGTGAATTGTCTGATGTGAAGAGGGTTTCAAGTC
ATCATCTTCGCCTTTTAGGGTTCAAGCCATTGGATTGCTTAAAAGATTATCATAACTTAA
GTCCATCAACATTTATTTACCCCAGTGATGAGCAAATATTCGGAAGCACTCGTGTTTTCG
TTGCTTTACATAGCTCGATGCTGCGTCTTGGACGGTTTGCACTTGCACTTTACGGACC
CCAACTCGCCCACGGCTTGTAGCCCTTGTTGCACAAGAAGAGGTTATTTCTTCGAGTG
GTCAAGATGAGCCACCTGGAATGCACATGATCTATCTTCCATACTCGGATGATGTTAGAT
ATCCTGAAGAGGTTCATCTGACTTCTGGGGATGCACCTCGCGCTACAGATGAGCAAAT
AAAGAAAGCTTCGAATCTGTTGAGACGTATTGACCTGAAGCATTTCTCAGTAAGCCATT
TTGCTAACCCAGGATTGCAAAAACACTATGGGATTTTGGAGGCCTTAGCTTTAGGTGAG
GATGAGATGCCGGATATAAAGGATGAGACCCTGCCCGATGAAGAAGGCTTGGCTAGGC
CAGGAGTAGTTAAAGCTATAGAAGAATTTAAGGCTGCAGTGTTTGGTGAAAACTATGA
CCAAGAGGAAGCTGAGGCAGCAGCAGCAAAAGGTGGGGCCTCAAAGAAGAGGAAA
GCAATTGCTGATGCAGCTTCACAGAAAAGCGCGGCTTATGACTGGGCAGACCTTGCTG
ATAATGGGAAGCTGAAGGACATGACGGTGATGGATTTGAAAACTTACTTGACGGCGCA
TGGACTCCCAGTTTCTGGTAAGAAAGACGCCATCATCAGCAGGATCTTGACTCATCTAG
GCAAGTGA
>J411-4
ATGGACCTGGACCCCGAGGGCATCTTCCGCGACGACAGCGACGAGGACGACGACAAC
CTCCATGAGAGGGAGGCCAACAAGGAGATGGTCGTCTACCTCATAGACGCCTCGCCCA
GGATGTTCACCCCCGCCAACGCCGCCAAGCCAGATGAAAAGCAGGAGACACATTTCC
ATACCATAGTGAACTGCATCACGCAGTCTCTTAAGATGCAGATTATCGGGAGATCCCGT
GATGAAGTTGCAATATGCTTCTTTAACACCAAAGAAAGGAAAAATTTACAGGAGCTGG
CTGGTGTATATGTTTACAATGTCACAGAAAGAGAGCAACTTGATAGGCCTGATGCAAG
ACTGATTAAAGAATTTTCTTGTGTAGAAGATTCTTTTATGAATAACATTGGAAGCCGGTA
TGGAATAACCTCTGGATCTCGAGAGAATACCCTGTACAATGCTCTTTGGGTTGCACAGG
CACTGCTGCGTAAAGGATCTGTGAAGACTGTGAGTAAGAGAATCCTCATATTCACCAAT

GAGGACGATCCTTTTGGTGGTATTACAGGAGCAGCAAAGACTGATATGATTAGGACCA
CAATTCAACGTGCAAAAGATGCACAAGATCTGGGCCTGTCTATCGAACTTCTTCCATTG
AGTAGGCCTGATGAGGATTTCAACATGTCCCTGTTTTATGCAGATTTGATTGGTCTGGA
GGGAGATGAAGTAGTGCAGTACGTGCCATCTGCTGGTGAAAAGCTGGAGGATATGACC
GATCAACTGAGAAAGCGAGTGATGAAAAAGCGCAAAGTCAAAACTCTCTCATTTGCA
ATTACAAATGATGTTTGCATAGAGGTGAACACATATGCGCTAATCCGTCCAACTGCTCC
AGGGACGATTACGTGGCTTGACTCGATCAGTAACCTTCCATTAAAGACTGAAAGGTCT
TTCATATGCAATGATACTGGGGCCCTTCTTCAGGCTCCTCAAGAGCGCTTCCAGCTATA
CAATGATAAAGTTGTGAAATTTTCTGTTCGTGAATTGTCTGATGTGAAGAGGGTTTCAA
GTCATCATCTTCGCCTTTTAGGGTTCAAGCCATTGGATTGCTTAAAAGATTATCATAACT
TAAGTCCATCAACATTTATTTACCCCAGTGATGAGCAAATGTTCGGAAGCACTCGTGTT
TTCGTTGCTTTACATAGCTCGATGCTGCGTCTTGGACGGTTTGCACTTGCATTTTACGGG
ACCCCAACTCGCCCACGGCTTGTAGCCCTTGTTGCACAAGAAGAGGTTATTTCTTCGA
GCGGTCAAGATGAGCCACCTGGAATGCACATGATCTATCTTCCATACTCGGATGATGTT
AGATATCCTGAAGAGGTTCATCTGACTTCTGGGGATGCACCTCGCGCTACAGATGAGC
AAATAAAGAAAGCTTCGAATCTGTTGAGACGTATTGACCTGAAGCATTTCTCAGTAAG
CCATTTTGCTAACCCAGGATTGCAAAAACACTATGGGATTTTGGAGGCCTTAGCTTTAG
GTGAGGATGAGATGCCGGATATACAGGATGAGACCCTGCCCGATGAAGAAGGCTTGGC
TAGGCCAGGAGTAGTTAAAGCTATAGAAGAATTTAAGGCTGCAGTGTTTGGTGAAAAC
TATGACCAAGAGGAAGCTGAGGCAGCAGCAGCAAAAGGTGGGGCCTCAAAGAAGAG
GAAAGCAATTGCTGATGCAGCTTCACAGAAAAGCGCGGCTTATGACTGGGCAGACCTT
GCTGATAATGGGAAGCTGAAGGACATGACGGTGATGGATTTGAAAACTTACTTGACGG
CGCATGGACTCCCAGTTTCTGGTAAGAAAGACGCCATCATCAGCAGGATCTTGACTCAT
CTAGGCAAGTGA
>J411-2
ATGGACCTGGACCCCGAGGGCATCTTCCGCGACGACAGCGACGAGGACGACGACAAC
CTCCATGAGAGGGAGGCCAACAAGGAGATGGTCGTCTACCTCATAGACGCCTCGCCCA
GGATGTTCACCCCCGCCAACGCCGCCAAGCCAGATGAAAAGCAGGAGACACATTTCC
ATACCATAGTGAACTGCATCACGCAGTCTCTGAAGATGCAGATTATCGGGAGATCCCGT
GATGAAGTTGCAATATGCTTCTTTAACACCAAAGAAAAGAAAAATTTACAGGAGCTGG
CTGGTGTATATGTTTACAATGTCACAGAAAGAGAGCAACTTGATAGGCCTGATGCAAG
ACTGATTAAAGAATTTTCTTGTGTAGAAGATTCTTTTATGAATAACATTGGAAGCCGGTA
TGGAATAACCTCTGGATCTCGAGAGAATACCCTGTACAATGCTCTTTGGGTTGCACAGG
CACTGCTGCGTAAAGGATCTGTGAAGACTGTGAGTAAGAGAATCCTCATATTCACCAAT
GAGGACGATCCTTTTGGTGGTATTACAGGAGCAGCAAAGACTGATATGATTAGGACCA
CAATTCAACGTGCAAAAGATGCACAAGATCTGGGCCTGTCTATCGAACTTCTTCCATTG
AGTAGGCCTGATGAGGATTTCAACATGTCCCTGTTTTATGCAGATTTGATTGGTCTGGA
GGGAGATGAAGTAGTGCAGTACGTGCCATCTGCTGGTGAAAAGCTGGAGGATATGACC
GATCAACTGAGAAAACGAATGATGAAAAAGCGCAAAGTCAAAACTCTCTCATTTGCAA
TTACAAATGATGTTTGCATAGAGGTGAACACATATGCGCTAATCCGTCCAACTGCTCCA
GGGACGATTACGTGGCTTGACTCGATCAGTAACCTTCCATTAAAGACTGAAAGGTCTTT
CATATGCAATGGTACTGGGGCCCTTCTTCAGGCTCCTCAAGAGCGCTTCCAGCTATACA
ATGATAAAGTTGTGAAATTTTCTGTTCGTGAATTGTCTGATGTGAAGAGGGTTTCAAGT
CATCATCTTCGCCTTTTAGGGTTCAAGCCATTGGATTGCTTAAAAGATTATCATAACTTA

AGTCCATCAACATTTATTTACCCCAGTGATGAGCAAATATTCGGAAGCACTCGTGTTTTC
GTTGCTTTACATAGCTCGATGCTGCGTCTTGGACGGTTTGCACTTGCATTTTACGGGAC
CCCAACTCGCCCACGGCTTGTAGCCCTTGTTGCACAAGAAGAGGTTATTTCTTCGAGT
GGTCAGGATGAGCCACCTGGAATGCACATGATCTACCTTCCATACTCGGATGATGTTAG
ATATCCTGAAGAGGTTCATCTGACTTCTGGGGATGCACCTCGCGCTACAGATGAGCAAA
TAAAGAAAGCTTCGAATCTGTTGAGACGTATTGACCTGAAGCATTTCTCAGTAAGCCAT
TTTGCTAACCCAGGATTGCAAAAACACTATGGGATTTTGGAGGCCTTAGCTTTAGGTGA
GGATGAGATGCCGGATATAAAGGATGAGACCCTGCCCGATGAAGAAGGCTTGGCTAGG
CCAGGAGTAGTTAAAGCTATAGAAGAATTTAAGGCTGCAGTGTTTGGTGAAAACTATG
ACCAAGAGGAAGCTGAGGCAGCAGCAGCAAAAGGTGGGGCCTCAAAGAAGAGGAA
AGCAATTGCTGATGCAGCTTCACAGAAAAGCGCGGCTTATGACTGGGCAGACCTTGCT
GATAATGGGAAGCTGAAGGACATGCCGGTGATGGATTTGAAAACCTACTTGACGGCGC
ATGGACTCCCAGTTTCTGGTAAGAAAGACGCCATCATCAGCAGGATCTTGACTCATCTA
GGCAAGTGA
>J411-13
ATGGACCTGGACCCCGAGGGCATCTTCCGCGACGACAGCGACGAGGACGACGACAAC
CTCCATGAGAGGGAGGCCAACAAGGAGATGGTCGTCTACCTCATAGACGCCTCGCCCA
AGATGTTCACACCCGCCAACGCCGCCAAGCCAGATGAAAAGCAGGAGACACATTTCC
ATACCATAGTGAACTGCATCACGCAGTCTCTGAAGACGCAGATTATCGGGAGATCCCGT
GATGAAGTTGCAATATGCTTCTTTAACACCAAAGAAAAGAAAAATTTACAGGAGCTGG
CTGGTGTATATGTTTACAATGTCACAGAAAGAGAGCAACTTGATAGACCTGATGCAAG
ACTGATTAAAGAATTTTCTTGTGTAGAAGATTCTTTTATGAATACCATTGGAAGCCGGTA
TGGAATAACCTCTGGATCTAGAGAGAATACCCTGTACAATGCTCTTTGGGTTGCACAGG
CACTGCTGCGTAAAGGATCTGTGAAGACTGTGAGTAAGAGAATCCTCATATTCACCAAT
GAGGATGATCCTTTTGGTGGTATTACAGGAGCAGCAAAGACTGATATGATTAGGACCAC
AATTCAACGTGCAAAAGATGCACAAGATCTGGGCCTGTCTATCGAACTTCTTCCATTGA
GTAGGCCTGATGAGGATTTCAACATGTCCCTGTTTTATGCAGATTTGATTGGTCTGGAG
GGAGATGAAGTACTGCAGTATGTGCCATCTGCTGGTGAAAAGCTGGAGGATATGACTG
ATCAACTGAGAAAGCGAATGATGAAAAAGCGCAAAGTCAAAACTCTCTCATTTGCAAT
TACAAATGATGTTTGCATAGAGGTGAACACATATGCGTTAATCCGTCCAACTGCTCCAG
GGACGATCATGTGGCTTGACTCGATCAGTAACCTTCCATTAAAGACTGAAAGGTCATTC
GTATGCAATGACACCGGGGCCCTTCTTCAGGCTCCCCAAGAGCGCTTCCAGCTATACAA
TGATAAAGTTGTTAAATTTTCTGTTCGTGAATTGTCTGATATGAAGAGGGTTTCAAGTCA
TCATCTTCGCCTTTTAGGGTTCAAGCCATTGAATTGCTTAAAAGATTATCATAACTTAAG
TCCATCAACATTTATTTACCCCAGTGATGAGCAAATATTCGGAAGCACTCGTGTGTTTGT
TGCTTTACATAGCTCGATGCTGCGTCTTGGAAGGTTTGCACTTGCGTTTTACGGGACCC
CAACTCGCCCACGGCTTGTAGCCCTTGTTGCACAAGAAGAGGTTATTTCTTCGAGTGG
TCAAGATGAGCCACCTGGAATACACATGATCTATCTTCCATATTCGGATGATGTTAGATA
TCCTGAAGAGGTTCATCTGACTTCTGGGGATGCACCTCGCGCAACAGATGAGCAAATA
AAGAAAGCTTCGAATCTGTTGAGACGTATTGACCTGAAGCATTTCTCAGTAAGCCATTT
TGCTAACCCAGGATTGCAAAAACACTATGGGATTTTGGAGGCCTTAGCTTTAGGTGAG
GATGAGATGCCGGATATAAAGGATGAGACCCTGCCCGATGAAGAAGGCTTGGCTAGGC
CAGGAGTAGTTAAAGCTATAGAAGAATTTAAGGCTGCAGTGTTTGGTGAAAATTATGAC
CAAGAGGAAGCTGAGGCAGCAGCAGCAAAAGGTGGGGCCTCAAAGAAGAGGAAAG

CAATTGCTGATGCAGCTTCACAGAAAAGCGCGGCCTATGATTGGGCAGACCTTGCAGA
TAATGGGAAGCTGAAGGACATGACGGTGATGGATTTGAAAACTTACCTGACGGCGCAT
GGCCTCCCAGTTTCTGGCAAGAAAGACGCCATCATCAGCAGGATCTTGACTCATCTAG
GCAAGTGA

>J411-5

ATGGACCTGGACCCCGAGGGCATCTTCCGCGACGACAGCGACGAGGACGACGACAAC
CTCCATGAGAGGGAGGCCAACAAGGAGATGGTCGTCTACCTCATAGACGCCTCGCCCA
AGATGTTCACACCCGCCAACGCCGCCAAGCCAGATGAAAAGCAGGAGACACATTTCC
ATACCATAGTGAACTGCATCACGCAGTCTCTGAAGACGCAGATTATCGGGAGATCCCGT
GATGAAGTTGCAATATGCTTCTTTAACACCAAAGAAAAGAAAAATTTACAGGAGCTGG
CTGGTGTATATGTTTACAATGTCACAGAAAGAGAGCAACTTGATAGACCTGATGCAAG
ACTGATTAAAGAATTTTCTTGTGTAGAAGATTCTTTTATGAATACCATTGGAAGCCGGTA
TGGAATAACCTCTGGATCTAGAGAGAATACCCTGTACAATGCTCTTTGGGTTGCACAGG
CACTGCTGCGTAAAGGATCTGTGAAGACTGTGAGTAAGAGAATCCTCATATTCACCAAT
GAGGATGATCCTTTTGGTGGTATTACAGGAGCAGCAAAGACTGATATGATTAGGACCAC
AATTCAACGTGCAAAAGATGCACAAGATCTGGGCCTGTCTATCGAACTTCTTCCATTGA
GTAGGCCTGATGAGGATTTCAACATGTCCCTGTTTTATGCAGATTTGATTGGTCTGGAG
GGAGATGAAGTACTGCAGTATGTGCCATCTGCTGGTGAAAAGCTGGAGGATATGACTG
ATCAACTGAGAAAGCGAATGATGAAAAAGCGCAAAGTCAAAACTCTCTCATTTGCAAT
TACAAATGGTGTTTGCATAGAGGTGAACACATATGCGTTAATCCGTCCAACTGCTCCAG
GGACGATCATGTGGCTTGACTCGATCAGTAACCTTCCATTAAAGACTGAAAGGTCATTC
ATATGCAATGACACCGGGGCCCTTCTTCAGGCTCCCCAAGAGCGCTTCCAGCTATACAA
TGATAAAGTTGTTAAATTTTCTGTTCGTGAATTGTCTGATGTGAAGAGGGTTTCAAGTC
ATCATCTTCGCCTTTTAGGGTTCAAGCCATTGGATTGCTTAAAAGATTATCATAACTTAA
GTCCATCAACATTTATTTACCCCAGTGATGAGCAAATATTCGGAAGCACTCGTGTGTTT
GTTGCTTTACATAGCTCGATGCTGCGTCTTGGAAGGTTTGCACTTGCGTTTTACGGGAC
CCCAACTCGCCCACGGCTTGTAGCCCTTGTTGCACAAGAAGAGGTTATTTCTTCGAGT
GGTCAAGATGAGCCACCTGGAATGCACATGATCTATCTTCCATATTCGGATGATGTTAGA
TATCCTGAAGAGGTTCATCTGACTTCTGGGGATGCACCTCGCGCTACAGATGAGCAAAT
AAAGAAAGCTTCGAATCTGTTGAGACGTATTGACCTGAAGCATTTCTCAGTAAGCCATT
TTGCTAACCCAGGATTGCAAAAACACTATGGGATTTTGGAGGCCTTAGCTATAGGTGAG
GATGAGATGCCGGATATAAAGGATGAGACCCTGCCCGATGAAGAAGGCTTGGCTAGGC
CAGGAGTAGTTAAAGCTATAGAAGAATTTAAGGCTGCAGTGTTTGGTGAAAATTATGAC
CAGGAGGAAGCTGAGGCAGCAGCAGCAAAAGGTGGGGCCTCAAAGAAGAGGAAAG
CAATTGCTGATGCAGCTTCACAGAAAAGCGCGGCCTATGATTGGGCAGACCTTGCAGA
TAATGGGAAGCTGAAGGACATGACGGTGATGGATTTGAAAACTTACCTGACGGCGCAT
GGCCTCCCAGTTTCTGGCAAGAAAGACGCCATCATCAGCAGGATCTTGACTCATCTAG
GCAAGTGA

>J411-8

ATGGACCTGGACCCCGAGGGCATCTTCCGCGACGACAGCGACGAGGACGACGACAAC
CTCCATGAGAGGGAGGCCAACAAGGAGATGGTCGTCTACCTCATAGACGCCTCGCCCA
AGATGTTCACACCCGCCAACGCCGCCAAGCCAGATGAAAAGCAGGAGACACATTTCC
ATACCATAGTGAACTGCATCACGCAGTCTCTGAAGACGCAGATTATCGGGAGATCCCGT
GATGAAGTTGCAATATGCTTCTTTAACACCAAAGAAAAGAAAAATTTACAGGAGCTGG

CTGGTGTATATGTTTACAATGTCACAGAAAGAGAGCAACTTGATAGACCTGATGCAAG
ACTGATTAAAGAATTTTCTTGTGTAGAAGATTCTTTTATGAATACCATTGGAAGCCGGTA
TGGAATAACCTCTGGATCTAGAGAGAATACCCTGTACAATGCTCTTTGGGTTGCACAGG
CACTGCTGCGTAAAGGATCTGTGAAGACTGTGAGTAAGAGAATCCTCATATTCACCAAT
GAGGATGATCCTTTTGGTGGTATTACAGGAGCAGCAAAGACTGATATGATTAGGACCAC
AATTCAACGTGCAAAAGATGCACAAGATCTGGGCCTGTCTATCGAACTTCTTCCATTGA
GTAGGCCTGATGAGGATTCCAACATGTCCCTGTTTTATGCAGATTTGATTGGTCTGGAG
GGAGATGAAGTACTGCAGTATGTGCCATCTGCTGGTGAAAAGCTGGAGGATATGACTG
ATCAACTGAGAAAGCGAATGATGAAAGAGCGCAAAGTCAAAACTCTCTCATTTGCAAT
TACAAATGATGTTTGCATAGAGGTGAACACATATGCGTTAATCCGTCCAACTGCTCCAG
GGACGATCATGTGGCTTGACTCGATCAGTAACCTTCCATTAAAGACTGAAAGGTCATTC
ATATGCAATGACACCGGGGCCCTTCTTCAGGCTCCCCAAGAGCGCTTCCAGCTATACAA
TGATAAAGTTGTTAAATTTTCTGTTCATGAATTGTCTGATGTGAAGAGGGTTTCAAGTC
ATCATCTTCGCCTTTTAGGGTTCAAGCCATTGGATTGCTTAAAAGATTATCATAACTTAA
GTCCATCAACATTTATTTACCCCAGTGATGAGCAAATATTCGGAAGCACTCGTGTGTTT
GTTGCTTTACATAGCTCGATGCTGCGTCTTGGAAGGTTTGCACTTGCGTTTTACGGGAC
CCCAACTCGCCCACGGCTTGTAGCCCTTGTTGCACAAGAAGAGGTTATTTCTTCGAGT
GGTCAAGATGAGCCACCTGGAATGCACATGATCTATCTTCCATATTCGGATGATGTTAGA
TATCCTGAAGAGGTTCATCTGACTTCTGGGGATGCACCTCGCGCTACAGATGAGCAAAT
AAAGAAAGCTTCGAATCTGTTGAGACGTATTGACCTGAAGCATTTCTCAGTAAGCCATT
TTGCTAACCCAGGATTGCAAAAACACTATGGGATTTTGGAGGCCTTAGCTTTAGGTGAG
GATGAGATGCCGGATATAAAGGATGAGACCCTGCCCGATGAAGAAGGCTTGGCTAGGC
CAGGAGTAGTTAAAGCTATAGAAGAATTTAAGGCTGCAGTGTTTGGTGAAAATTATGAC
CAAGAGGAAGCTGAGGCAGCAGCAGCAAAAGGTGGGGCCTCAAAGAAGAGGAAAG
CAATTGCTGATGCAGCTTCACAGAAAAGCGCGGCCTATGATTGGGCAGACCTTGCAGA
TAATGGGAAGCTGAAGGACATGACGGTGATGGATTTGAAAACTTACCTGACAGCGCAT
GGCCTCCCAGTTTCTGGCAAGAAAGACGCCATCATCAGCAGGATCTTGACTCATCTAG
GCAAGTGA
>J411-18
ATGGACCTGGACCCCGAGGGCATCTTCCGCGACGACAGCGACGAGGACGACGACAAC
CTCCATGAGAGGGAGGCCAACAAGGAGATGGTCGTCTACCTCATAGACGCCTCGCCCA
AGATGTTCACACCCGCCAACGCCGCCAAGCCAGACGAAAAGCAGGAGACACATTTCC
ATACCATAGTGAACTGCATCACGCAGTCTCTGAAGACGCAGATTATCGGGAGATCCCGT
GATGAAGTTGCAATATGCTTCTTTAACACCAAAGAAAAGAAAAATTTACAGGAGCTGG
CTGGTGTATATGTTTACAATGTCACAGAAAGAGAGCAACTTGATAGACCTGATGCAAG
ACTGATTAAAGAATTTTCTTGTGTAGAAGATTCTTTTATGAATACCATTGGAAGCCGGTA
TGGAATAACCTCTGGATCTAGAGAGAATACCCTGTACAATGCTCTTTGGGCTGCACAGG
CACTGCTGCGTAAAGGATCTGTGAAGACTGTGAGTAAGAGAATCCTCATATTCTCCAAT
GAGGATGATCCTTTTGGTGGTATTACAGGAGCAGCAAAGACTGATATGATTAGGACCAC
AATTCAACGTGCAAAAGATGCACAAGATCTGGGCCTGTCTATCGAACTTCTTCCATTGA
GTAGGCCTGATGAGGATTTCAACATGTCCCTGTTTTATGCAGATTTGATTGGTCTGGAG
GGAGATGAAGTACTGCAGTATGTGCCATCTGCTGGTGAAAAGCTGGAGGATATGACTG
ATCAACTGAGAAAGCGAATGATGAAAAAGCGCAAAGTCAAAACTCTCTCATTTGCAAT
TACAAATGATGTCTGCATAGAGGTGAACACATATGCGTTAATCCGTCCAACTGCTCCAG

GGACGATCATGTGGCTTGACTCGATCAGTAACCTTCCATTAAAGACTGAAAGGTCATTC
ATATGCAATGACACCGGGGCCCTTCTTCAGGCTCCCCAAGAGCGCTTCCAGCTATACAA
TGATAAAGTTGTTAAATTTTCTGTCCGTGAATTGTCTGATGTGAAGAGGGTTTCAAGTC
ATCATCTTCGCCTTTTAGGGTTCAAGCCATTGGATTGCTTAAAAGATTATCATAACTTAA
GTCCATCAACATTTATTTACCCCAGTGATGAGCGAATATTCGGAAGCACTCGTGTGTTT
GTTGCTTTACATAGCTCGATGCTGCGTCTTGGAAGGTTTGCACTTGCGTTTTACGGGAC
CCCAACTCGCCCACGGCTTGTAGCCCTTGTTGCACAAGAAGAGGTTATTTCTTCGAGT
GGTCAAGATGAGCCACCTGGAATGCACATGATCTATCTTCCATATTCGGATGATGTTAGA
TATCCTGAAGAGGTTCATCTGACTTCTGGGGATGCACCTCGCGCTACAGATGAGCAAAT
AAAGAAAGCTTCGAATCTGTTGAGACGTATTGACCTGAAGCATTTCTCAGTAAGCCATT
TTGCTAACCCAGGATTGCAAAAACACTATGGGATTTTGGAGGCCTTAGCTTTAGGTGAG
GATGAGATGCCGGATATAAAGGATGAGACCCTGCCCGATGAAGAAGGCTTGGCTAGGC
CAGGAGTAGTTAAAGCTATAGAAGAATTTAAGGCTGCAGTGTTTGGTGAAAATTATGAC
CAAGAGGAAGCTGAGGCAGCAGCAGCAAAAGGTGGGGCCTCAAAGAAGAGGAAAG
CAATTGCTGATGCAGCTTCACAGAAAAGCGCGGCCTATGATTGGGCAGACCTTGCAGA
TAATGGGAAGCTGAAGGACATGACGGTGATGGATTTGAAAACTTACCTGACGGCGCAT
GGCCTCCCAGTTTCTGGCAAGAAAGACGCCATCATCAGCAGGATCTAGACTCATCTAG
GCAAGTGA

.>J411-20

ATGGACCTGGACCCCGAGGGCATCTTCCGCGACGACAGCGACGAGGACGACGACAAC
CTCCATGAGAGGGAGGCCAACAAGGAGATGGTCGTCTACCTCATAGACGCCTCGCCCA
AGATGTTCACACCCGCCAACGCCGCCAAGCCAGATGAAAAGCAGGAGACACATTTCC
ATACCATAGTGAACTGCATCACGCAGTCTCTGAAGACGCAGATTATCGGGAGATCCCGT
GATGAAGTTGCAATATGCTTCTTTAACACCAAAGAAAAGAAAAATTTACAGGAGCTGG
CTGGTGTATATGTTTACAATGTCACAGAAAGAGAGCAACTTGATAGACCTGATGCAAG
ACTGTTTAAAGAATTTTCTTGTGTAGAAGATTCTTTTATGAATACCATTGGAAGCCGGTA
TGGAATAACCTCTGGATCTAGAGAGAATACCCTGTACAATGCTCTTTGGGTTGCACAGG
CACTGCTGCGCAAAGGATCTGTGAAGACTGTGAGTAAGAGAATCCTCATATTCACCAA
TGAGGATGATCCTTTTGGTGGTATTACAGGAGCAGCAAAGACTGATATGATTAGGACCA
CAATTCAACGTGCAAAAGATGCACAAGATCTGGGCCTGTCTATCGAACTTCTTCCATTG
AGTAGGCCTGATGAGGATTTCAACATGTCCCTGTTTTATGCAGATTTGATTGGTCTGGA
GGGAGATGAAGTACTGCAGTATGTGCCATCCGCTGGTGAAAAGCTGGAGGATATGACT
GATCAACTGAGAAAGCGAATGATGAAAAAGCGCAAAGTCAAAACTCTCTCATTTGCAA
TTACAAATGATGTTTGCATAGAGGTGAACACATATGCGTTAATCCGTCCAACTGCTCCA
GGGACGATCATGTGGCTTGACTCGATCAGTAACCTTCCATTAAAGACTGAAAGGTCATT
CATATGCAATGACACCGGGGCCCTTCTTCAGGCTCCCCAAGAGCGCTTCCAGCTATACA
ATGATAAAGTTGTTAAATTTTCTGTTCGTGAATTGTCTGATGTGAAGAGGGTTTCAAGT
CATCATCTTCGCCTTTTAGGGTTCAAGCCATTGGATTGCTTAAAAGATTATCATAACTTA
AGTCCATCAACATTTATTTACCCCAGTGATGAGCAAATATTCGGAAGCACTCGTGTGTT
TGTTGCTTTACATAGCTCGATGCTGCGTCTTGGAAGGTTTGCACTTGCGTTTTACGGGA
CCCCAACTCGCCCACGGCTTGTAGCCCTTGTTGCACAAGAAGAGGTTATTTCTTCGAG
TGGTCAAGATGAGCCACCTGGAATGCACATGATCTATCTTCCATATTCGGATGATGTTAG
ATATCCTGAAGAGGTTCATCTGACTTCTGGGGATGCACCTCGCGCTACAGATGAGCAAA
TAAAGAAAGCTTCGAATCTGTTGAGACGTATTGACCTGAAGCATTTCTCAGTAAGCCAT

TTTGCTAACCCAGGATTGCAAAAACACTATGGGATTTTGGAGGCCTTAGCTATAGGTGA
GGATGAGATGCCGGATATAAAGGATGAGACCCTGCCCGATGAAGAAGGCTTGGCTAGG
CCAGGAGTAGTTAAAGCTATAGAAGAATTTAAGGCTGCAGTGTTTGGTGAAAATTATGA
CCAGGAGGAAGCTGAGGCAGCAGCAGCAAAAGGTGGGGCCTCAAAGAAGAGGAAA
GCAATTGCTGATGCAGCTTCACAGAAAAGCGCGGCCTATGATTGGGCAGACCTTGCAG
ATAATGGGAAGCTGAAGGACATGACGGTGATGGATTTGAAAACTTACCTGACGGCGCA
TGGCCTCCCAGTTTCTGGCAAGAAAGACGCCATCATCAGCAGGATCTTGACTCATCTA
GGCAAGTGA

>J411-22

ATGGACCTGGACCCCGAGGGCATCCTCCGCGACGACAGCGACGAGGACGACGACAAC
CTCCATGAGAGGGAGGCCAACAAGGAGATGGTCGTCTACCTCATAGACGCCTCGCCCA
AGATGTTCACACCCGCCAACGCCGCCAAGCCAGATGAAAAGCAGGAGACACATTTCC
ATACCATAGTGAACTGCATCACGCAGTCTCTGAAGACGCAGATTATCGGGAGATCCCGT
GATGAAGTTGCAATATGCTTCTTTAACACCAAAGAAAAGAAAAATTTACAGGAGCTGG
CTGGTGTATATGTTTACAATGTCACAGAAAGAGAGCAACTTGATAGACCTGATGCAAG
ACTGATTAAAGAATTTTCTTGTGTAGAAGATTCTTTTATGAATACCATTGGAAGCCGGTA
TGGAATAACCTCTGGATCTAGAGAGAATACCCTGTACAATGCTCTTTGGGTTGCACAGG
CACTGCTGCGTAAAGAATCTGTGAAGACTGTGAGTAAGAGAATCCTCATATTCACCAAT
GAGGATGATCCTTTTGGTGGTATTACAGGAGCAGCAAAGACTGATATGATTAGGACCAC
AATTCAACGTGCAAAAGATGCACAAGATCTGGGCCTGTCTATCGAACTTCTTCCATTGA
GTAGGCCTGATGAGGATTTCAACATGTCCCTGTTTTATGCAGATTTGATTGGTCTGGAG
GGAGATGAAGTACTGCAGTATGTGCCATCTGCTGGTGAAAAGCTGGAGGATATGACTG
ATCAACTGAGAAAGCGAATGATGAAAAAGCGCAAAGTCAAAACTCTCTCATTTGCAAT
TACAAATGATGTTTGCATAGAGGTGAACACATATGCGTTAATCCGTCCAACTGCTCCAG
GGACGATCATGTGGCTTGACTCGATCAGTAACCTTCCATTAAAGACTGAAAGGTCATTC
ATATGCAATGACACCGGGGCCCTTCTTCAGGCTCCCCAAGAGCGCTTCCAGCTATACAA
TGATAAAGTTGTAAAATTTTCTGTTCGTGAATTGTCTGATGTGAAGAGGGTTTCAAGTC
ATCATCTTCGCCTTTTAGGGTTCAAGCCATTGGATTGCTTAAAAGATTATCATAACTTAA
GTCCATCAACATTTATTTACCCCAGTGATGAGCAAATATTCGGAAGCACTCGTGTGTTT
GTTGCTTTACATAGCTCGATGCTGCGTCTTGGAAGGTTTGCACTTGCGTTTTACGGACC
CCAACTCGCCCACGGCTTGTAGCCCTTGTTGCACAAGAAGAGGTTATTTCTTCGAGTG
GTCAAGATGAGCCACCTGGAATGCACATGATCTATCTTCCATATTCGGATGATGTTAGAT
ATCCTGAAGAGGTTCATCTGACTTCTGGGGATGCACCTCGCGCTACAGATGAGCAAAT
AAAGAAAGCTTCGAATCTGTTGAGACGTATTGACCTGAAGCATTTCTCAGTAAGCCATT
TTGCTAACCCAGGATTGCAAAAACACTATGGGATTTTGGAGGCCTTAGCTTTAGGTGAG
GATGAGATGCCGGATATAAAGGATGAGACCCTGCCCGATGAAGAAGGCTTGGCTAGGC
CAGGAGTAGTTAAAGCTATAGAAGAATTTAAGGCTGCAGTGTTTGGTGAAAATTATGAC
CAAGAGGAAGCTGAGGCAGCAGCAGCAAAAGGTGGGGCCTCAAAGAAGAGGAAAG
CAATTGCTGATGCAGCTTCACAGAAAAGCGCGGCCTATGATTGGGCAGACCTTGCAGA
TAATGGGAAGCTGAGGGACATGACGGTGATGGATTTGAAAACTTACCTGACGGCGCAT
GGCCTCCCAGTTTCTGGCAAGAAAGACGCCATCATCAGCAGGATCTTGACTCATCTAG
GCAAGTGA

>J411-21

ATGGACCTGGACCCCGAGGGCATCTTCCGCGACGACAGCGACGAGGACGACGACAAC

CTCCATGAGAGGGAGGCCAACAAGGAGATGGTCGTCTACCTCATAGACGCCTCGCCCA
AGATGTTCACACCCGCCAACGCCGCCAAGCCAGATGAAAAGCAGGAGACACATTTCC
ATACCATAGTGAACTGCATCACGCAGTCTCTGAAGACGCAGATTATCGGGAGATCCCGT
GATGAAGTTGCAATATGCTTCTTTAACACCAAAGAAAAGAAAAATTTACAGGAGCTGG
CTGGTGTATATGTTTACAATGTCACAGAAAGAGAGCAACTTGATAGACCTGATGCAAG
ACTGATTAAAGAATTTTCTTGTGTAGAAGATTCTTTTATGAATACCATTGGAAGCCGGTA
TGGAATAACCTCTGGATCTAGAGAGAATACCCTGTACAATGCTCTTTGGGTTGCACAGG
CACTGCTGCGTAAAGGATCTGTGAAGACTGTGAGTAAGAGAATCCTCATATTCACCAAT
GAGGATGATCCTTTTGGTGGTATTACAGGAGCAGCAAAGACTGATATGATTAGGACCAC
AATTCAACGTGCAAAAGATGCACAAGATCTGGGCCTGTCTATCGAACTTCTTCCATTGA
GTAGGCCTGATGAGGATTTCAACATGTCCCTGTTTTATGCAGATTTGATTGGTCTGGAG
GGAGATGAAGTACTGCAGTATGTGCCATCTGCTGGTGAAAAGCTGGAGGATATGACTG
ATCAACTGAGAAAGCGAATGATGAAAAAGCGCAAAGTCAAAACTCTCTCATTTGCAAT
TACAAATGATGTTTGCATAGAGGTGAACACATATGCGTTAATCCGTCCAACTGCTCCAG
GGACGATCATGTGGCTTGACTCGATCAGTAACCTTCCATTAAAGACTGAAAGGTCATTC
ATATGCAATGACACCGGGGCCCTTCTTCAGGCTCCCCAAGAGCGCTTCCAGCTATACAA
TGAGTAAGTACACTAAAGTTGTTAAATTTTCTGTTCGTGAATTGTCTGATGTGAAGAGG
GTTTCAAGTCATCATCTTCGCCTTTTAGGGTTCAAGCCATTGGATTGCTTAAAAGATTAT
CATAACTTAAGTCCATCAACATTTATTTACCCCAGTGATGAGCAAATATTCGGAAGCACT
CGTGTGTTTGTTGCTTTACATAGCTCGATGCTGCGTCTTGGAAGGTTTGCACTTGCGTT
TTACGGGACCCCAACTCGCCCACGGCTTGTAGCCCTTGTTGCACAAGAAGAGGTTATT
TCTTCGAGTGGTCAAGATGAGCCACCTGGAATGCACATGATCTATCTTCCATATTCGGAT
GATGTTAGATATCCTGAAGAGGTTCATCTGACTTCTGGGGATGCACCTCGCGCTACAGA
TGAGCAAATAAAGAAAGCTTCGAATCTGTTGAGACGTATTGACCTGAAGCATTTCTCA
GTAAGCCATTTTGCTAACCCAGGATTGCAAAAACACTATGGGATTTTGGAGGCCTTAGC
TTTAGGTGAGGATGAGATGCCGGATATAAAGGATGAGACCCTGCCCGATGAAGAAGGC
TTGGCTAGGCCAGGAGTAGTTAAAGCTATAGAAGAATTTAAGGCTGCAGTGTCTGGTG
AAAATTATGACCAAGAGGAAGCTGAGGCAGCAGCAGCAAAAGGTGGGGCCTCAAAG
AAGAGGAAAGCAATTGCTGATGCAGCTTCACAGAAAAGCGCGGCCTATGATTGGGCA
GACCTTGCAGATAATGGGAAGCTGAAGGACATGACGGTGATGGATTCGAAAACTTACC
TGACGGCGCATGGCCTCCCAGTTTCTGGCAAGAAAGACGCCATCATCAGCAGGATCTT
GACTCATCTAGGCAAGTGA
