# Supplementary material for: Factors Affecting the Radiosensitivity of Hexaploid Wheat to γ-Irradiation: Radiosensitivity of Hexaploid Wheat (Triticum aestivum L.)
Source: PLoS One. 2016 Aug 23;11(8):e0161700. doi: 10.1371/journal.pone.0161700 (PMC4995049; doi:10.1371/journal.pone.0161700)
Supplement: S1 Table — (PDF) [file pone.0161700.s005.pdf]

Primer	Sequence（5’→3’）
TaKu70 QF	CTACCTCATAGACGCCTCGC
TaKu70 QR	GCAACTTCATCACGGGATCT
TaKu80 QF	GGCTGGTTCTGCTGCTGGATG
TaKu80 QR	GCCAACCTCGTCGCTCCTATG
TaActin QF	GTAGGAAATGGCTGACGGTG
TaActin QR	ATGCTAGGGAAAACAGCCCT
18s QF	CCATCCCTCCGTAGTTAGCTTCT
18s QR	CCTGTCGGCCAAGGCTATATAC
