# Supplementary material for: Correction: Factors Affecting the Radiosensitivity of Hexaploid Wheat to γ-Irradiation: Radiosensitivity of Hexaploid Wheat (Triticum aestivum L.)
Source: PLoS One. 2016 Oct 20;11(10):e0165187. doi: 10.1371/journal.pone.0165187 (PMC5072575; doi:10.1371/journal.pone.0165187)
Supplement: S1 File — (PDF) [file pone.0165187.s001.pdf]

PLOS ONE

RESEARCH ARTICLE

Factors Affecting the Radiosensitivity of Hexaploid Wheat to -Irradiation: Radiosensitivity of Hexaploid Wheat (*Triticum aestivum* L.)

Bing Han[1,2☯], **Jiayu Gu**[2☯], **Linshu Zhao**[2], **Huijun Guo**[2], **Yongdun Xie**[2], **Shirong Zhao**[2], **Xiyun Song**[1], **Longzhi Han**[2], **Luxiang Liu**[2]*

1 Academy of Life Science, Qingdao Agricultural University, Qingdao, China, **2** Institute of Crop Sciences, Chinese Academy of Agricultural Sciences /National Key Facility for Crop Gene Resources and Genetic Improvement /National Center of Space Mutagenesis for Crop Improvement, Beijing, China

☯ These authors contributed equally to this work.
* liuluxiang@caas.cn

CrossMark
click for updates

OPEN ACCESS

Citation: Han B, Gu J, Zhao L, Guo H, Xie Y, Zhao S, et al. (2016) Factors Affecting the Radiosensitivity of Hexaploid Wheat to -Irradiation: Radiosensitivity of Hexaploid Wheat (*Triticum aestivum* L.). PLoS ONE 11(8): e0161700. doi:10.1371/journal.pone.0161700

Editor: Arthur J. Lustig, Tulane University Health Sciences Center, UNITED STATES

Received: April 3, 2016

Accepted: August 10, 2016

Published: August 23, 2016

Copyright: © 2016 Han et al. This is an open access article distributed under the terms of the Creative Commons Attribution License, which permits unrestricted use, distribution, and reproduction in any medium, provided the original author and source are credited.

Data Availability Statement: All relevant data are within the paper and its Supporting Information files.

Funding: This work was supported by the National 973 Program: grant no. 2014CB138101, the National Key Technology R&D Program of China: grant no.2014BAA03B04, Core Research Budget of the Non-profit Governmental Research Institutions (ICS, CAAS), and the National Natural Science Foundation Research Program: grant no. 11305261.

Competing Interests: The authors have declared that no competing interests exist.

Abstract

Understanding the radiosensitivity of plants, an important factor in crop mutation breeding programs, requires a thorough investigation of the factors that contribute to this trait. In this study, we used the highly radiosensitive wheat (*Triticum aestivum* L.) variety HY1 and J411, a γ-irradiation-insensitive control, which were screened from a natural population, to examine the factors affecting radiosensitivity, including free radical content and total antioxidant capacity, as well as the expression of *TaKu70* and *TaKu80* (DNA repair-related genes) as measured by real-time PCR. We also investigated the alternative splicing of this gene in the wild-type wheat ecotype by sequence analysis. Free radical contents and total antioxidant capacity significantly increased upon exposure of HY1 wheat to γ-irradiation in a dose-dependent manner. By contrast, in J411, the free radical contents exhibited a similar trend, but the total antioxidant capacity exhibited a downward trend upon increasing γ-irradiation. Additionally, we detected dose-dependent increases in *TaKu70* and *TaKu80* expression levels in γ-irradiated HY1, while in J411, *TaKu70* expression levels increased, followed by a decline. We also detected alternative splicing of *TaKu70* mRNA, namely, intron retention, in HY1 but not in J411. Our findings indicate that γ-irradiation induces oxidative stress and DNA damage in hexaploid wheat, resulting in growth retardation of seedlings, and they suggest that *TaKu70* may play a causal role in radiosensitivity in HY1. Further studies are required to exploit these factors to improve radiosensitivity in other wheat varieties.

Introduction

The induced mutation technique, an important application of nuclear technology in agriculture, has significantly contributed to crop germplasm enhancement and new mutant variety

development. Studies investigating radiation sensitivity have played prominent roles in revealing the mechanism of induced mutations. Differences in the radiosensitivity of various crops have been investigated [1–3]. However, the molecular mechanisms responsible for radiosensitivity are currently unclear, making research in this area vital. Investigations in animals and human have revealed a link between cell radiosensitivity and variations in free radicals, oxidative stress, and DNA repair mechanisms [4–10]. Therefore, it is important to further explore radiosensitivity and its molecular determinants. Such information would be useful for predicting and modulating radiosensitivity.

Organisms exposed to irradiation are induced to produce reactive oxygen species (ROS), which can give rise to DNA double-strand breaks (DSBs), which in turn can affect proteins. However, some plants, during the long process of evolution, have developed the ability to withstand ionizing radiation (IR) stress. Under IR stress, such plants can activate ROS removal systems and DNA repair systems. The former includes enzyme and non-enzyme based systems. The enzyme systems utilize amongst others, SOD, GPX and catalase to quickly remove ROS. Non-enzymatic systems utilize, amongst others, the surfhydryl of glutathione (GSH), ascorbic acid and the carotenoqesls to quench oxyradicals [10–16]. Currently, it is widely accepted that oxidative stress is involved in the pathogenesis of many diseases, including various cancers and degenerative disorders in animals [17]. In our study, we assess the effects of ROS on hexaploid wheat, as well as its total antioxidative capacity (T-AOC). DSBs caused by IR is disastrous to both cells and organisms. It can trigger chromosomal rearrangements, aneuploidy and the loss of segments of chromosomes. If not followed by rapid and successful repair, cell and whole organism death can be infamous result [18–21]. In eukaryotes, Non-homologous end-joining (NHEJ) is the most important DSB repair pathway [22–25]. ku70/ku80 heterodimers recognize DSBs and bind to them, forming complexes. These recruit DNA-dependent protein kinase catalytic subunits which initiate the NHEJ repair process [26–29], Errors in or DNA mutations involving this process can lead to failure of repair and so increase the sensitivity of the organism to ionizing radiation [30]. In our study, defects in the *TaKu70* gene may be one reason for the high sensitivity of our HY1 strain. Under normal circumstances, alternative splicing of premature RNA is an important process utilized by eukaryotes to produce all kinds of protein forms from a single gene [31–34]. This can enhance protein diversity and regulates some process in plants [35–37]. Some studies have suggested that the alternative splicing of single genes can create small amounts of protein isoforms in plants. In rice, approximately 68.3% of genes create only one isoform. However, among all splicing types, intron retention is common [38–40]. In wheat, investigations are ongoing. However, with regards mechanisms and function, there seem to be few surprises [41]. Evidence suggests that weak splice sites, shorter introns and lower density splicing enhancers intron retention [42–43]. Therefore, their retention suggests the occurrence of missplicing, caused by problems with the splicing machinery [44]. In addition, alternative splicing regulates the expression of certain critical genes [45–48].

Any defect of alternative splicing can cause severe problems to the organism as a result of changes in their protein composition. Frame shifts are one consequence of miss-splicing [36]. In Arabidopsis 42% of fame shift events create a premature stop codon; in rice, the frequency is 36% [32]. In our study, intron retention was found in the *TaKu70* gene. This may reflect splicing errors in the pre-mRNA splicing process, prematurely stopping the expression of *TaKu70*. Ultimately, defective TaKu70 protein might be produced.

Phenotype studies of ku70-defects in DNA repair mechanisms have been carried out in fungi, yeasts and animals [49]. Disruption of Ku70 in mouse embryonic stem cells results in markedly increased sensitivity to ionizing radiation [50]. Ku70-deficient mice are approximately half the size of control mice, and their fibroblasts are sensitive to ionizing radiation and display premature senescence associated with the accumulation of nondividing cells [51]. In

Arabidopsis, *ku* mutants can be associated with telomere elongation [52]. In our study, *TaKu70* defective wheat was highly sensitivity to γ radiation. This produced severe phenotypic changes compared to the controls.

We previously cloned *Ku70* and *Ku80* in wheat, which were designated *TaKu70* and *TaKu80*, respectively [53–55]. The functions of the two genes, as well as the encoded protein, have been investigated [55]. We previously selected the hexaploid wheat variety HY1, which exhibited the highest sensitivity to γ-irradiation among the 63 wheat genotypes examined, whereas wheat variety J411 exhibited insensitivity to γ-irradiation[56]. In this study, the increased radiosensitivity of this variety allowed us to analyze the combined effects of an exogenous agent and IR on plants[14]. The results of this study may help shed light on the mechanism underlying radiosensitivity in wheat.

Materials and Methods

γ-irradiation and free radical contents assay

The moisture content of the dry seeds of HY1 and J411 was balanced with glycerin and water ratio of 1:1 to up to 13% and they were then irradiated by gamma rays at dosages of 100, 150 and 250 Gy (7 Gy/min; The Department of Radiation at Peking University, Beijing, China). The free radical contents under each dosage were immediately examined using electron spin resonance apparatus (ESR, E-Scan, BRUKER, SC0340, Germany), and various parameters were obtained, namely Food, Marker, g1-value, g2-value, Frequency. The seeds under each dosage were randomly examined five times. The Food parameters values, which represent variations in free radicals, were normalized using the formula: Foodn = Food × 400000 / Marker. Foodn values can take the place of relative free radical contents values.

Plant materials and cultivation

All wheat seeds were kindly provided by the Chinese Academy of Agricultural Sciences. Prior to placing them on a hydroponics shelf for seedling growth, the dried seeds were immersed in distilled water for germination for 16 hours with a rate of 50 seeds/15 mL water (using three replicates per dosage). Germinated seeds were placed on a hydroponics shelf under controlled conditions (16 h light/12 h dark, temperature: 25°C, relative humidity: 78%). Leaves were harvested on the day 5 for DNA and RNA extraction.

Total antioxidative capacity (T-AOC) assay

The irradiated seeds were soaked in distilled water for 16 h (using three replicates per dosage) and transferred to the germination apparatus under constant conditions. Leaf samples (0.05 g) were collected on day 5, instantly placed into 2 mL EP tubes containing 500 μL 0.9% normal saline and ground into a powder using a tissue grinder apparatus (30 Hz/s, 60 s). The samples were centrifuged for 300 s at 5,000 rpm, and 300 μL of supernatant was transferred to a fresh 1.5 mL centrifuge tube (on ice). The T-AOC assay was carried out using a T-AOC reagent kit, and the OD value at 520 nm was measured following the manufacturer's instructions using the formula$T - AOC = (ODu - ODc) / 0.01 / 30 * N / CProt$.

DNA isolation, PCR amplification and sequencing

Genomic DNA was obtained from a pool of DNA extracted from ten HY1 and J411 leaves using a Caliper workstation and a DNA Secure Plant Kit. To determine the source of the retained fragment in the cDNA, specific primers were designed (25AER: 5′ GGCACTGCTG CGTAAAGG 3′ , 25AEF: 5′ TCACCAGCAGATGGCACG 3′) based on the A, B and D genome

sequences [55]. For amplification and sequencing of the 879 bp gene containing the 133 bp retention fragment, primers were designed approximately 20 to 50 bp upstream and downstream of the coding region. For analysis of the region, the fragments of accessions were amplified using the proofreading polymerase Phusion (Finnzymes) and subjected to TA clone prior to sequencing. The positive clones were directly used for sequencing.

RNA isolation, cDNA synthesis and real-time PCR

RNA was isolated with TRNzol^{A+}solution. For each accession, three biological replicates were performed, and 1 μg RNA was reversely transcribed using a Transcriptor High Fidelity First-strand cDNA Synthesis Kit (Roche, Version 6, Germany). For the *TaKu70* gene, 10 accessions were selected for RT-PCR and TA cloning. For each accession, 72 positive clones were selected for sequencing and analyses. Primary analyses revealed alternative splicing in the cDNA fragment from the HY1 accessions, which was confirmed using a validation protocol. Data analysis, sequence alignment and were performed with BioEdit version 7.05 for each accession, and all variable sites were checked manually during the construction of a sequence contig. All sequences were manually aligned to the reference sequence. RNA was reversely transcribed and used at 1 μg per real-time-PCR run in a 10 μL reaction volume using SsoFast™ EvaGreen Supermix and a C1000™ thermal cycler; each biological replicate included three technical replicates. Expression was normalized to the *Actin* and *18s* genes. The primer sequences are shown in S1 Table. A CFx96™ Real-time System was used for analysis.

Data analysis

Statistical analysis was performed using SPSS 16.0, with one-way ANOVA performed to test the significance of differences when more than two groups were involved. Values were considered significantly different if ($P < 0.05$). Comprehensive data analysis was performed using Heml1.0 software.

Results

Variation in free radical contents

To investigate the oxidative stress caused by gamma irradiation, we measured the free radical contents in HY1 wheat and the control variety J411 in response to various dosages of IR (Fig 1). The relative free radical content increased significantly in a dose-dependent manner. At 250 Gy, the relative free radical levels were significant increased in these plants, reaching more than 3.0-fold control dosage levels (under 0 Gy treatment). The free radical contents were more than 2.0-fold control levels under 100 Gy treatment and 2.5-fold control levels under 150 Gy treatment. These levels were significantly higher in the treatment groups than in the control dosage group ($p < 0.05$). In the control variety J411, the relative free radical contents increased in a dose-dependent manner, but the upward trend was slower. The basal levels of free radicals in HY1 and J411 were similar.

Total antioxidant capacity (T-AOC)

To analyze the roles of enzymatic and non-enzymatic components in HY1 and the control variety J411, namely, the T-AOC of these plants, we used a total antioxidant capacity kit to measure OD values at 520 nm, which revealed T-AOC values under each dosage of IR (Fig 2). In HY1, the T-AOC values increased significantly with increasing gamma irradiation dosage. Under 250 Gy treatment, T-AOC reached more than two-times the levels measured under the control dosage levels. At lower doses, however, increases in T-AOC values were drastically

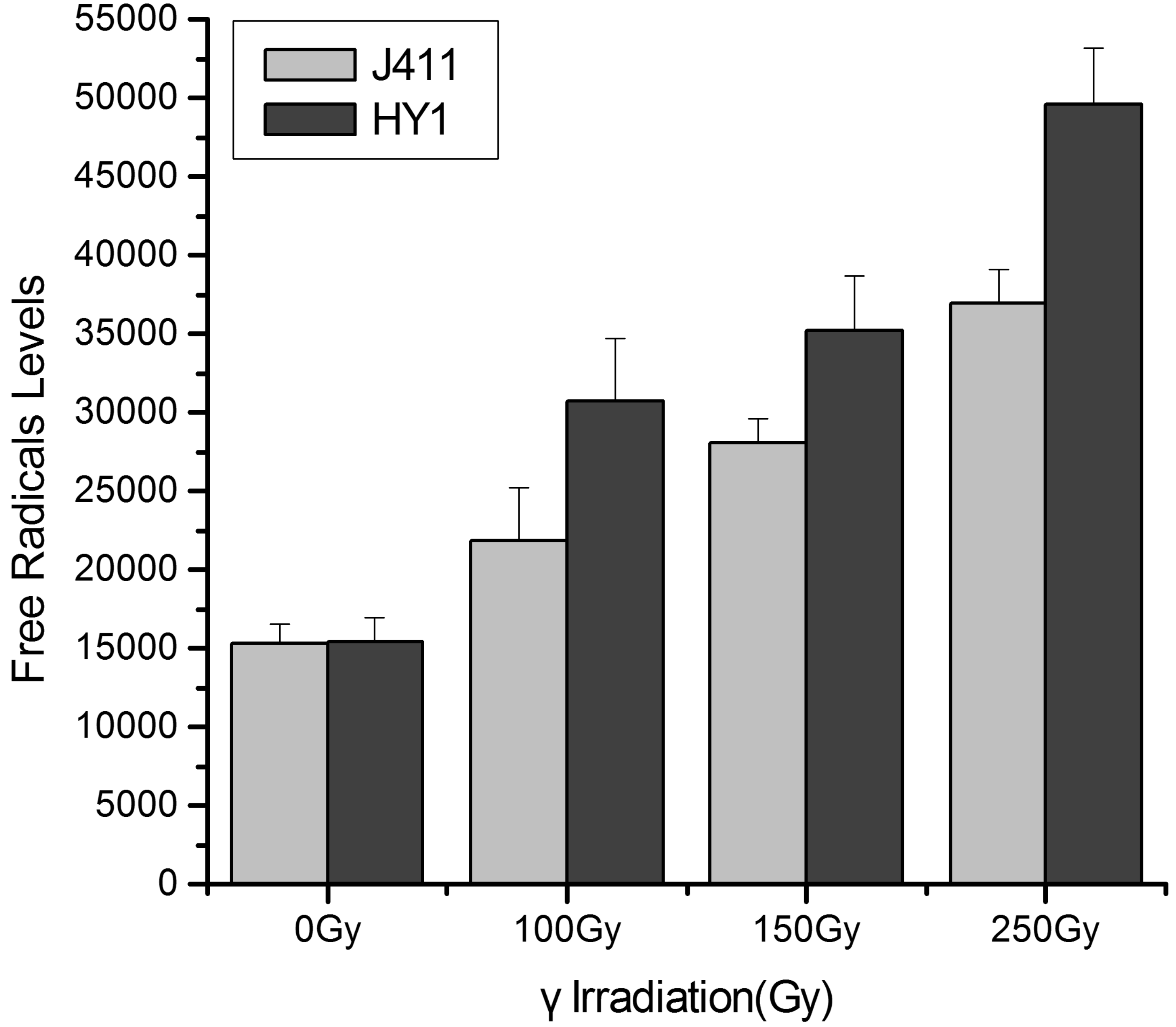

Fig 1. Effect of gamma irradiation on free radical levels. The X-axis represents the treatment dosage, including 0 Gy, 100 Gy, 150 Gy and 250 Gy. The Y-axis represents the free radical levels. Dark gray bars indicate free radical contents in HY1, light gray bars represent free radical contents in the J411 variety. Significant differences between treatment groups and the control groups in the HY1and J411 variety were analyzed by spass 16.0 ($P < 0.05$).

doi:10.1371/journal.pone.0161700.g001

lower, approaching the values of the control dosage group, The basal T-AOC was higher in the control variety J411 than in HY1, whereas at 100 Gy and 150 Gy, these values were consistent in the two varieties, and at 250 Gy, this value was lower in J411 than the HY1.

DNA repair-related gene expression

To investigate changes in the expression of the DNA repair-related genes *TaKu70* and *TaKu80* in response to gamma irradiation, we monitored the expression of these genes in IR-treated

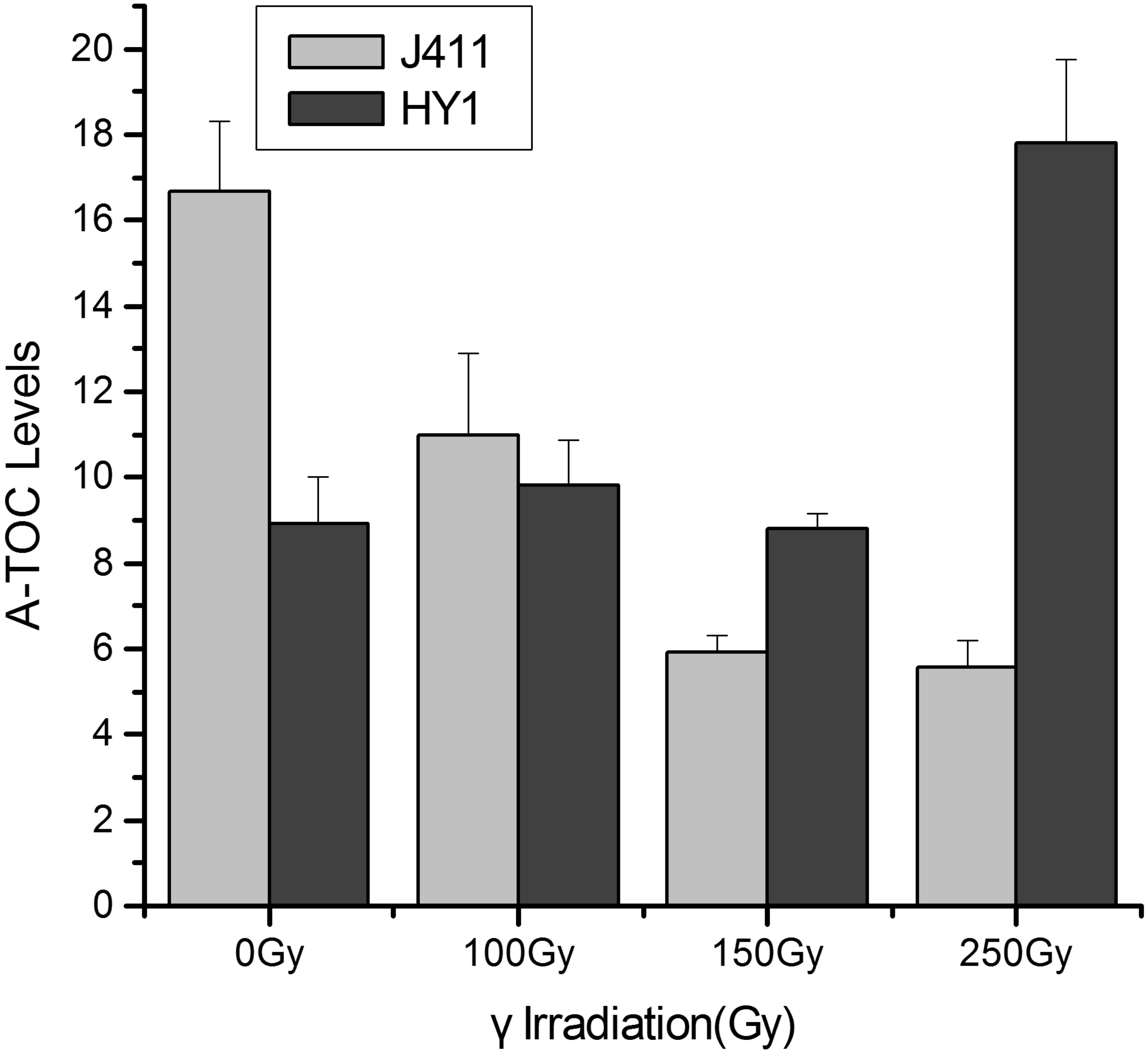

Fig 2. Effect of gamma irradiation on T-AOC in HY1 and J411 wheat. The X-axis represents the treatment dosage, including 0 Gy, 100 Gy, 150 Gy and 250 Gy. The Y-axis represents T-AOC levels. Dark gray bars represent T-AOC values in HY1, and light gray bars represent those in J411. Significant differences between treatment groups and the control groups in the HY1and J411 variety were analyzed by spass 16.0 ($P < 0.05$).

doi:10.1371/journal.pone.0161700.g002

HY1 and the control variety J411 (Fig 3E and 3F). In HY1, under high doses of gamma irradiation, both genes were significantly induced, especially *TaKu70*; at 250 Gy, the expression level of this gene was more than three-times that of the control(0Gy). However, under 100 Gy treatment, both genes were only slightly induced. The phenotypes of HY-1 in response to 0, 100, 150, and 250 Gy gamma irradiation are shown in Fig 3B, Seedling height and root length significantly decreased with increasing gamma irradiation dosage. while in the control variety J411, *TaKu70* was significantly induced at dosages of 100 Gy and 150 Gy. At 100 Gy, the expression level of this gene was more than two-times that of the control dose (0 Gy). *Taku80* expression

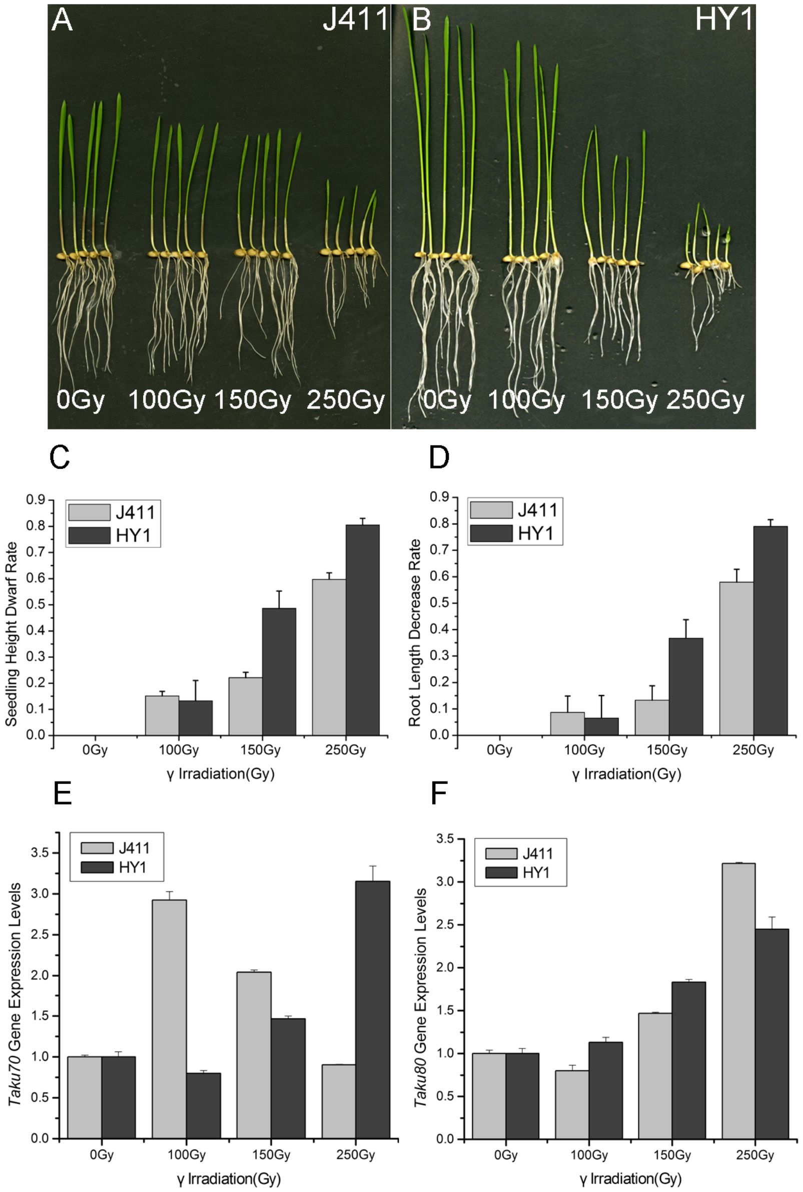

Fig 3. Effect of gamma irradiation on DNA repair-related genes *TaKu70* and *TaKu80 and plant phenotypes.* (A, B) Photographs of HY1 and J411 plants under different dosages of γ-irradiation. Seedling height and root length decreased significantly with increasing gamma irradiation dosage more quickly in HY1 than in J411. (C, D) Histogram analysis of variation rate of root length and seedling height in HY1 and J411. The X-axis represents the treatment dosage, including 0 Gy, 100 Gy, 150 Gy and 250 Gy. The Y-axis represents the variation rate of root length and seedling in HY1 and J411. Significant differences were analyzed by spass 16.0 ($P < 0.05$) (E, F)The X-axis represents the treatment dosage, including 0 Gy, 100 Gy, 150 Gy and 250 Gy. The Y-axis represents *Taku70* gene expression level. Dark gray bars indicate *Taku70* and *Taku80* gene expression values in HY1, and light gray bars indicate those in J411. Significant differences between treatment groups and the control groups in the HY1and J411 variety were analyzed by spass 16.0 ($P < 0.05$).

doi:10.1371/journal.pone.0161700.g003

levels were consistent with the levels detected in HY1. The phenotypes of J411 in response to IR are shown in Fig 3A. Seedling height and root length decreased slightly with increasing gamma irradiation dosage. Histogram analysis of seedling height and root length in HY1 and the control variety J411 is shown in Fig 3C and 3D. Seedling height decreased significantly with

increasing gamma irradiation dosage in both HY1 and J411, but the reduction was slower in J411 than in HY1 (Fig 3C and 3D)

Intron retention in *TaKu70*

To explore the transcriptional regulation of *TaKu70*, which affects the radiosensitivity of HY1 and the control variety J411, we cloned *TaKu70* cDNA sequences (S2 Fig) from both varieties and subjected the mRNA sequences to alignment (Fig 4A). In HY1, cDNA sequence alignment (S2 Fig) showed that a 133 bp fragment located between 601 bp and 733 bp was retained in its mRNA sequence. A comparison between HY1 *TaKu70* cDNA and the wild-type *TaKu70* sequence (S1 Fig) showed that the retained 133 bp fragment was derived from the sixth intron (67 bp) located between 2,544 bp and 2,610 bp and the seventh intron (66 bp) located between

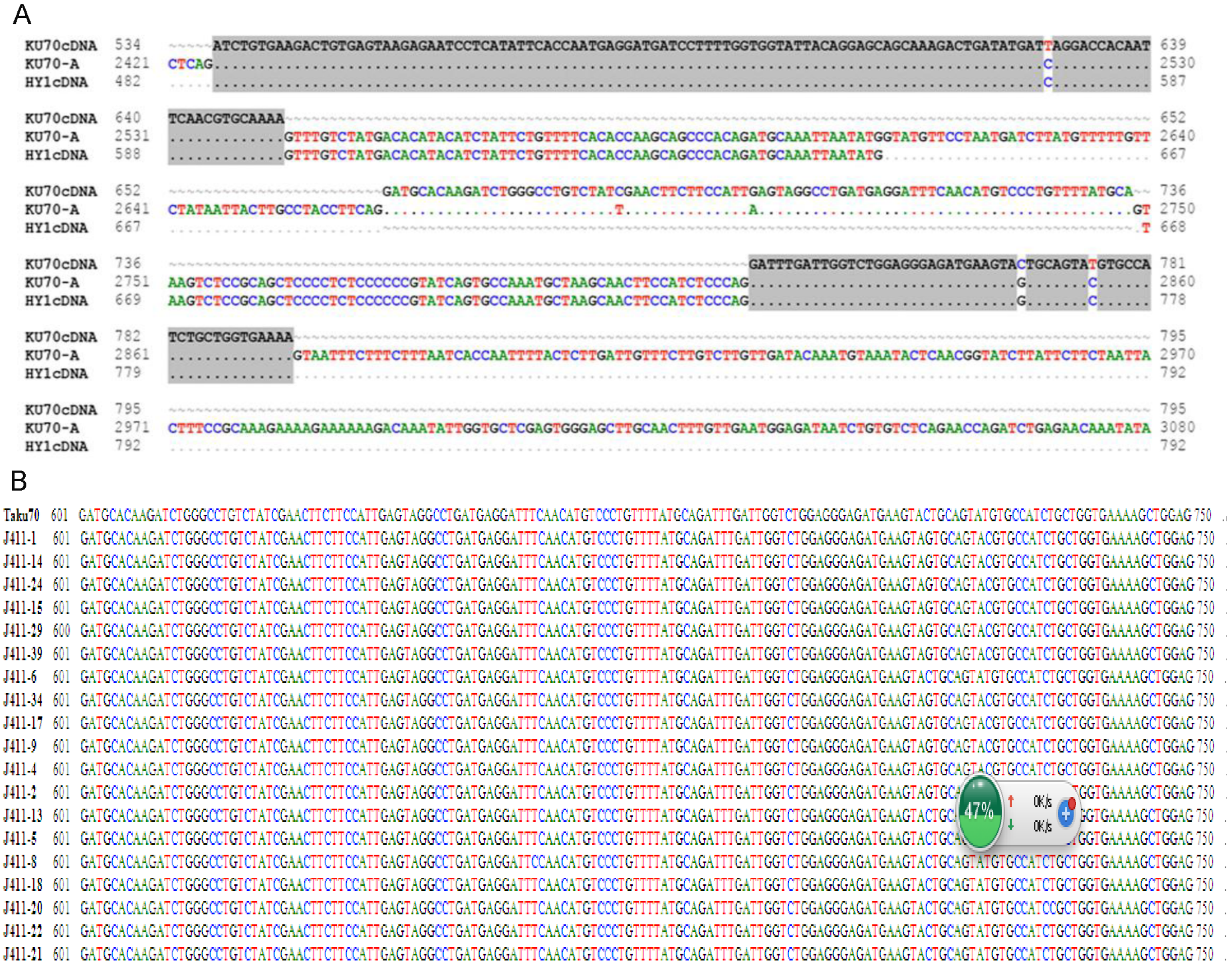

Fig 4. Analysis of the alternative splicing, namely, intron retention. (A) The first region highlighted in gray represents the sixth exon., and the second represents the eighth exon. Ku70-cDNA and the ku70 –A sequence were obtained from the Chinese spring variety cloned by our team as the standard comparison sequence. (B) J411-1, J411-14, J411-24 s equence, and so on represent 19 clone replicates from J411.

doi:10.1371/journal.pone.0161700.g004

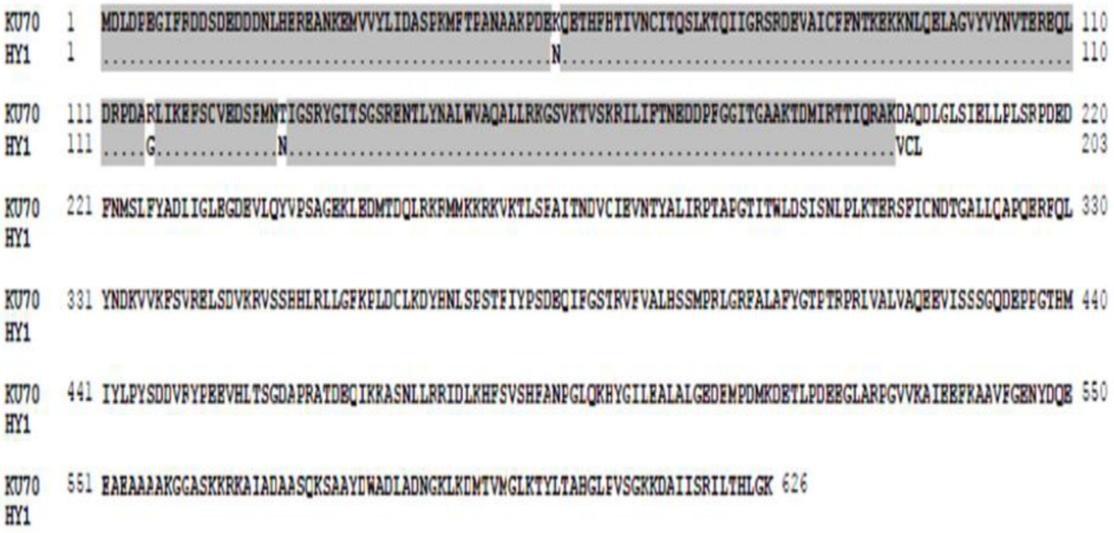

Fig 5. The effect of intron retention on the encoded protein. *TaKu70* encodes a 626 amino acid protein. Gray highlighting represents the protein encoded by the mRNA harboring intron retention in HY1.

doi:10.1371/journal.pone.0161700.g005

2,750 bp and 2,815 bp in the A genome (Fig 4A). In J411, 19 clone replicates were carried out, and is no retention was detected in its mRNA (Fig 4B, S4 Fig).

Amino acid analysis of encoded *TaKu70* protein

As shown in Fig 5, normal *TaKu70* mRNA encodes a functional protein, TaKu70, containing 626 amino acid residues. However, *TaKu70* exhibiting intron retention encodes a non-functional TaKu70 protein of only 200 amino acid residues (S3 Fig). Whether this protein is actually produced and functional remains unclear.

Integrated analysis of all data

Heml1.0 software was used to construct a heat map of all of the data. As shown in Fig 6, the clustering results suggest that the phenotypic variation is related to *TaKu70* expression and T-AOC levels. Phenotypic variation is a standard measure of radiosensitivity. In the J411 variety, the A-TOC levels, *TaKu70* expression levels, phenotypes and radicals levels were clustered together then *TaKu80* expression levels. On the Y-axis, 100 Gy and 150 Gy were clustered together (Fig 6A). In the HY1 variety, *TaKu70* and *TaKu80* expression levels, T-AOC and radicals levels were clustered together, which ultimately influence the phenotype. On the Y-axis, 0 Gy and 100 Gy were clustered then150 Gy (Fig 6B).

Discussion

The molecular mechanisms responsible for radiosensitivity are currently unclear, making this topic a vital area of research. In the current study, we explored the molecular determinants of radiosensitivity. We chose the hexaploid wheat variety HY1 and the variety J411 as a control: the former was previously found to be the most susceptible variety to gamma irradiation and the latter is not radiosensitive to gamma irradiation among the 63 wheat genotypes examined [55–56]. To date, several studies examining the effects of gamma irradiation have been carried out in terrestrial organisms and some aquatic vertebrates [57–59]. Some studies suggest that

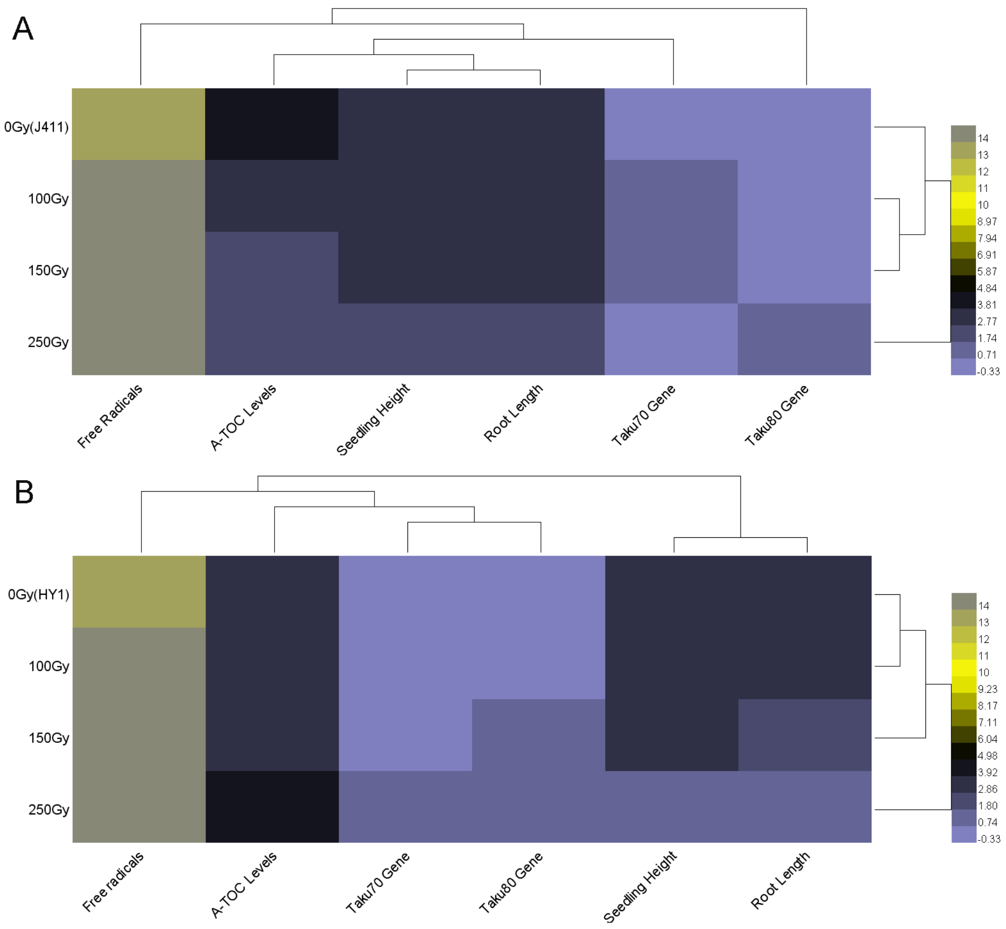

Fig 6. Heat map analysis of all data. The abscissa represents free radical levels, A-TOC levels, seedling height, root length, *TaKu70* and *TaKu80* expression levels. The ordinate represents different dosages of γ-irradiation. The primary data were LOG_2 transformed using Heml1.0 software. The color variation represents different values.

doi:10.1371/journal.pone.0161700.g006

different levels of susceptibility to radiation between organisms are likely due to differences in DNA content, repair processes, and cell cycle kinetics [60]. In the current study, we found that the high radiosensitivity of HY1 may be linked to the DNA repair process and A-TOC levels (Fig 6). However, the underlying mechanisms that function in organisms upon gamma irradiation have not been investigated in detail.

Free radicals play an important role in radiosensitivity. We therefore assessed the free radicals levels of the control variety J411 and HY1, finding that both of these levels increased upon exposure to radiation, but the increase was more rapid in HY1 than in J411 (Fig 1). This result suggests that under the same γ-irradiation conditions, J411 may produce fewer free radicals than HY1. The accumulation of free radicals can lead to DSBs and a variety of molecular effects, including preventing cell division, aging and apoptosis [61]. Organisms utilize enzymatic and non-enzymatic systems to counteract the effects of free radicals in an attempt to maintain cellular homeostasis [62]. We therefore examined the T-AOC of enzymatic and non-enzymatic antioxidants using a total antioxidant capacity kit. HY1 had lower basal A-TOC levels than J411. The HY1 cells exhibited lower T-AOC under lower doses of gamma irradiation (100 Gy and 150 Gy), but quickly increased at high doses (250 Gy). While in J411, the basal T-AOC level was

higher than that of HY1, and the T-AOC levels decreased with increasing irradiation (Fig 2). The cause of this difference is currently unclear. Perhaps the high dose of γ-irradiation induced some enzymatic activities, and the primordial T-AOC substance can remove the induced T-AOC substance in the J411 variety. These results all suggest that enzymatic and non-enzymatic antioxidants play main role in resisting irradiation stress in the two varieties. Additionally, the enzymatic and non-enzymatic system comprises several components, such as SOD, GSH, CAT, GR, GST, GPX, and so on [63]. The total antioxidant enzyme activities displayed in Fig 2 may not represent the effect of each individual enzyme. However, interestingly, the expression of the DNA repair-related gene *TaKu70* (Fig 3E) dramatically increased under 100 Gy gamma irradiation and then began to decline in J411. In addition, the *TaKu70* mRNA sequence lacked a retention segment (Fig 4B), suggesting that J411 *TaKu70* mRNA encodes the normal protein and has completed the DNA repair progress, which may be an important cause of the low radiosensitivity of this variety. In HY1, *TaKu70* was significantly upregulated at 150 Gy and 250 Gy. However, segment retention occurred in the mRNA of HY1, which may encode a nonfunctional protein (Fig 4A). This segment retentionb may be an important cause of the radiosensitivity of HY1. These results suggest that the DNA repair system may greatly contribute to the varied radiosensitivity of J411 and HY1. The different A-TOC levels in the two varieties likely contributes to this difference as well. Comprehensive heat map analysis of all of the data (Fig 6A and 6B) helped confirm the above-mentioned results. Some researchers have proposed that gene transcription levels are a reliable early signal for detecting physiological changes under environmental stress [64–65]. Elucidating the expression patterns of specific genes would be helpful for better understanding the underlying molecular mechanisms of radiosensitivity upon γ-irradiation. *Ku70* and *Ku80* are induced by γ-irradiation in a dose-dependent manner in the marine copepod *Paracyclopina nana* [63]. In human, Ku70, and Ku80 are key components of the DSBs repair process, as they ligate the broken ends of DNA in the absence of homologous templates [66]. Thus, the increased expression of *TaKu70* and *TaKu80* in gamma-irradiated HY1 and J411 implies that these genes are closely related to the enhanced DNA repair process that functions to recover oxidative stress-induced cellular damage in these plants.

RNA sequencing confirmed that intron retention occurred in *TaKu70* mRNA (Fig 4A) in HY1, which may also help explain the increased radiosensitivity of this genotype. The 133 bp fragment retained in the mRNA would lead to the production of a 200 amino acid, non-functional TaKu70 protein (Fig 5). Thus, DNA repair of DSBs would be weakened or inhibited, which may contribute to the high radiosensitivity of the HY1 variety. Disruption of Ku70 in mouse embryonic stem cells results in severely increased sensitivity to ionizing radiation [50]. The aborted DNA repair process might cause the phenotypic variation observed in HY1 (Fig 3A), Ku70-deficient mice are approximately 50% the size of the control [51], the phenotypic effects of different doses of radiation were reconfirm in our experiments (Fig 3A and 3B). However, our knowledge of the physiological relevance of this important post-transcriptional regulatory mechanism in plants is quite limited. The current study provides functional evidence that alternative splicing plays a important role in plant responses to environmental stress [41]. Our data open up the possibility for further study of a probable link between alternative splicing and hypersensitivity to γ-ionizing radiation in plants,

In summary, our results suggest that there is a correlation between radiosensitivity and intron retention, as well as activation of the antioxidant and DNA repair systems, in HY1. No previous studies have investigated the radiosensitivity mechanism in other plants. In this study, we found that the antioxidant and DNA repair systems were induced by gamma irradiation to mitigate damage from free radicals. Additionally, alternative splicing, namely intron retention, might contribute to the radiosensitivity of HY1. Further evidence is needed to confirm the correlation between radiosensitivity and intron retention in hexaploid wheat.

Supporting Information

S1 Fig. The comparison of HY1 TaKu70 cDNA, *Ku70-A* and *TaKu70* cDNA. Ku70-A genome and Taku70cDNA was cloned from the Chinese spring. HY1Taku70 cDNA was cloned from HY1.
(PDF)

S2 Fig. The comparsion of HY1 TaKu70 cDNA and *TaKu70* cDNA. 133 bp fragment located between 601 bp and 733 bp was retained in the mRNA sequence in HY1.
(PDF)

S3 Fig. Amino acid analysis of encoded TaKu70 protein. Taku70 encodes a 626 amino acid residues protein. The detained mRNA encoded 200 amino acid residues protein.
(PDF)

S4 Fig. The original sequences of the 19 clone repeats of *Taku70* gene mRNA from J411 variety.
(PDF)

S1 Table. primer Squence used in the Quantitative Real-time PCR.
(PDF)

Acknowledgments

We would like to thank all the members who involved in the experiment in this study, The authors deeply appreciate all the technicians for their assitance in experiments. We particularly thank the reviewers to give us the constructive suggestions about the manuscript.

Author Contributions

Conceptualization: LL XS BH JG LH.

Data curation: LL BH.

Formal analysis: BH.

Funding acquisition: LL JG.

Investigation: BH JG.

Methodology: BH JG HG.

Project administration: BH LZ.

Resources: LL LZ HG YX SZ.

Software: JG.

Supervision: LL XS YX SZ LH.

Validation: LL XS.

Visualization: BH JG.

Writing – original draft: BH.

Writing – review & editing: YS BH.

References

1. Soriano JD. The response of several rice varieties to fast neutrons. Radiation Botany.1971; 11(5):341–346.
2. Inoue M, Ito R, Tabata T, Hasegawa H. Varietal difference in the repair of gamma irradiation induced Lesions in barley. Environmental and Experimental Botany.1980; 20(2):161–168.
3. Baldik R, Aytekin H, Erer M. Radioactivity measurements and radiation dose assessments due to natural radiation in Karabük (Turkey). Journal of Radioanalytical and Nuclear Chemistry. 2011; 289(2): 297–302.
4. Rhee JS, Kim BM, Kang CM, Lee YM, Lee JS. Gamma irradiation-induced oxidative stress and developmental impairment in the hermaphroditic fish, Kryptolebias marmoratus embryo. Environ Toxicol Chem. 2012; 31(8):1745–53. doi: 10.1002/etc.1873 PMID: 22553164
5. Aquino KAS. Gamma radiation:Sterilization by gamma irradiation.p171. Croatia: In-Tech Press,2012
6. Grygoryev D, Moskalenko O, Hinton TG, Zimbrick JD. DNA damage caused by chronic transgenerational exposure to low dose gamma irradiation in Medaka fish(Oryzias latipes). Radiation Research. 2013; 180(3):235–46. doi: 10.1667/RR3190.1 PMID: 23919310
7. Mothersill C, Smith R, Lariviere D, Seymour C. Chronic exposure by ingestion of environmentally relevant doses of (226)Ra leads to transient growth perturbations in fathead minnow(Pimephales promelas, Rafinesque,1820). Int J Radiat Biol. 2013; 89(11):950–64. doi: 10.3109/09553002.2013.809817 PMID: 23724911
8. Smith RW, Seymour CB, Moccia RD, Hinton TG, Mothersill CE. The induction of a radiation-induced bystander effect in fish transcends taxonomic group and trophic level. Int J Radiat Biol. 2013; 89 (4):225–33. doi: 10.3109/09553002.2013.754558 PMID: 23206292
9. Sundarraj S, Thangam R, Sujitha MV, Kannan S. Ligand-conjugated mesoporous silica nanorattles based on enzyme targeted prodrug delivery system for effective lung cancer therapy. Toxicol Appl Pharmacol. 2014; 275(3):232. doi: 10.1016/j.taap.2014.01.012 PMID: 24467950
10. Jayakumar S, Kunwar A, Sandur SK, Pandey BN, Chaubey RC. Differential response of DU145 and PC3 prostate cancer cells to ionizing radiation: role of reactive oxygen species, GSH and Nrf2 in radiosensitivity. Biochim Biophys Acta.2014; 1840(1):485–94. doi: 10.1016/j.bbagen.2013.10.006 PMID: 24121106
11. Yu BP. Cellular defenses against damage from reactive oxygen species. Physiol Rev.1994; 74 (1):139–162. PMID: 8295932
12. Krogh BO, Symington LS. Recombination proteins in yeast. Annu Rev Genet.2004; 38: 233–271. PMID: 15568977
13. Jamieson DJ. Oxidative stress responses of the yeast Saccharomyces cerevisiae. Yeast.1998; 14 (16):1511–27. PMID: 9885153
14. Kim JK, Park J, Ryu TH, Nili M. Effect of N-acetyl-l-cysteine on Saccharomyces cerevisiae irradiated with gamma-rays.Chemosphere.2013; 92(5):512–6. doi: 10.1016/j.chemosphere.2013.02.035 PMID: 23623538
15. Esnault M, Legue F, Chenal C. Ionizing radiation: advances in plant response. Environmental and Experimental Botany. 2010; 68(3): 231–237
16. Winston DW, Di Giulio RT. Prooxidant and antioxidant mechanisms in aquatic organisms. Aquat Toxicol.1991; 19:137–161.
17. Bai H, Liu R, Chen HL, Zhang W, Wang X, Zhang X D, et al. Enhanced antioxidant effect of caffeic acid phenethyl ester and Trolox in combination against radiation induced-oxidative stress. Chem Biol Interact.2014; 207:7–15. doi: 10.1016/j.cbi.2013.10.022 PMID: 24211618
18. Khanna KK, Jackson SP. DNA double-strand breaks: signaling, repair and the cancer connection. Nat Gene.2001; 27(3):247–54.
19. Postow L, Ghenoiu C, Woo EM, Krutchinsky AN, Chait BT, Funabiki H. Ku80 removal from DNA through double strand break-induced ubiquitylation. J Cell Biol. 2008; 182(3):467–79. doi: 10.1083/jcb.200802146 PMID: 18678709
20. Arlt MF, Rajendran S, Birkeland SR, Wilson TE, Glover TW, John HJ, et al. De novo CNV formation in mouse embryonic stem cells occurs in the absence of Xrcc4-dependent nonhomologous end joining. PLoSGenet. 2012; 8(9):e1002981.
21. Li H, Vogel H, Holcomb VB, Gu Y, Hasty P. Deletion of *Ku70*, *Ku80*, or both causes early aging without substantially increased cancer. Mol cell Biol. 2007; 27(23):8205–14. PMID: 17875923
22. Gullo C, Au M, Feng G, Teoh G. The biology of Ku and its potential oncogenic role in cancer. Biochim Biophys Acta,2006; 1765(2):223–34. PMID: 16480833

23. Yan CT, Boboila C, Souza EK, Franco S, Hicknell TR, Murphy M, et al. IgH class switching and translocations use a robust non-classical end-joining pathway. Nature. 2007; 449(7161): 478–82. PMID: 17713479
24. Lieber MR, Ma Y, Pannicke U, Schwarz K. The mechanism of vertebrate nonhomologous DNA end joining and its role in V(D)J recombination.DNA repair(Amst).2004; 3(8–9):817–26.
25. Lin IT, Chao JL, Yao MC. An essential role for the DNA breakage-repair protein Ku80 in programmed DNA rearrangements in Tetrahymena thermophila. Mol Biol Cell.2012; 23(11):2213–25. doi: 10.1091/mbc.E11-11-0952 PMID: 22513090
26. Muller C, Rodrigo G, Calsou P, Salles B. DNA-dependent protein kinase: a major protein involved in the cellular response to ionizing radiation]. Bull cancer.1999; 86(12):977–83. PMID: 10660692
27. Takahashi T, Masuda T, Koyama Y. Enhanced gene targeting frequency in ku70 and ku80 disruption mutants of Aspergillus sojae and Aspergillus oryzae. Mol Genet Genomics. 2006; 275(5):460–70. PMID: 16470383
28. Korabiowska M, Konig F, Schlott T. Uantitative analysis of Ku70 and Ku80 mRNA gene expressionin melanoma brain metastases. Correlation with immunohisto chemistry and in situ hybridization. Cancer Genomics Proteomics.2004; 1:225–230
29. Walker JR, Corpina RA, Goldberg J. Structure of the Ku heterodimer bound to DNA and its implications for double-strand break repair. Nature. 2001; 412(6847):607–14. PMID: 11493912
30. Kurimasa A, Ouyang H, Dong LJ, Wang S, Li X, Cordon-Cardo C, et al. Catalytic subunit of DNA-dependent protein kinase: impact on lymphocyte development and tumorigenesis. Proc Natl Sci U S A.1999; 96(4):1403–8.
31. Venables JP. Aberrant and alternative splicing in cancer. Cancer Res. 2004; 64(21):7647–54. PMID: 15520162
32. Wang GS, Cooper TA. Splicing in disease: disruption of the splicing code and the decoding machinery. Nat Rev Genet. 2007; 8(10):749–61. PMID: 17726481
33. Chen M, Manley JL. Mechanisms of alternative splicing regulation: insights from molecular and genomics approaches. Nat Rev Mol Cell Biol. 2009; 10(11):741–54 doi: 10.1038/nrm2777 PMID: 19773805
34. NunoL BM, Irimia M, Pan Q, Xiong HY, Gueroussov S, et al. The Evolutionary Land scape of Alternative Splicing in Vertebrate Species. Science.2012; 338:1587–1593. doi: 10.1126/science.1230612 PMID: 23258890
35. Matlin AJ, Clark F, Smith CW. Understanding alternative splicing: towards a cellular code. Nat Rev Mol Cell Biol.2005; 6(5):386–98. PMID: 15956978
36. Keren H, Lev-Maor G, Ast G. Alternative splicing and evolution: diversification, exon definition and function. Nat Rev Genet.2010; 11(5):345–55. doi: 10.1038/nrg2776 PMID: 20376054
37. Zhang C, Yang H, Yang H. Evolutionary character of alternative Splicing in plants. Bioinform Biol Insights. 2016; 9(S1) 47–52.
38. Kim E, Goren A, Ast G. Alternative splicing: current perspectives. Bioessays.2008; 30(1):38–47. PMID: 18081010
39. May GE, Olson S, McManus CJ, Graveley BR. Competing RNA secondary structures are required for mutually exclusive splicing of the Dscam exon 6 cluster. RNA. 2011; 17(2):222–9. doi: 10.1261/rna.2521311 PMID: 21159795
40. Campbell MA, Haas BJ, Hamilton JP, Mount SM, Buell CR. Comprehensive analysis of alternative splicing in rice and comparative analyses with Arabidopsis BMC Genomics.2006; 7: 327. PMID: 17194304
41. Filichkin S, Priest HD, Megraw M, Mockler TC. Alternative splicing in plants: directing traffic at the crossroads of adaptation and environmental stress. Curr Opin Plant Biol,2015; 24:125–35. doi: 10.1016/j.pbi.2015.02.008 PMID: 25835141
42. Sakabe NJ, de Souza SJ. Sequence features responsible for intron retention in human. BMC genomics. 2007; 8:59. PMID: 17324281
43. Keren H, Lev-Maor G, Ast G. Alternative splicing and evolution: diversification, exon definition and function. Nature reviews Genetics.2010; 11(5):345–55 doi: 10.1038/nrg2776 PMID: 20376054
44. Ast G. How did alternative splicing evolve? Nat Rev Genet.2004; 5(10):773–82. PMID: 15510168
45. Tazi J, Bakkour N, Stamm S. Alternative splicing and disease. Biochimica et Biophyssica Acta1, 2009; 792: 14–26
46. Pillmann H, Hatije K, Odronitz F, Hammesfahr B, Kollmar M. Predicting mutually exclusive spliced exons based on exon length, splice site and reading frame conservation, and exon sequence homology. BMC Bioinformatics.2011; 12:270. doi: 10.1186/1471-2105-12-270 PMID: 21718515

47. Yeoh LM, Goodman CD, Hall NE, Van Dooren GG, Mc Fadden GI, Ralph SA. A serine–arginine-rich (SR) splicing factor modulates alternative splicing of over a thousand genes in *Toxoplasma gondii*. Nucleic Acids Res. 2015; 43(9): 4661–4675. doi: 10.1093/nar/gkv311 PMID: 25870410

48. Li X, Huang L, Zhang Y, Ouyang Z, Hong Y, Zhang H, et al. Tomato SR/CAMTA transcription factors SlSR1 and SlSR3L negatively regulate disease resistance response and SlSR1L positively modulates drought stress tolerance. BMC Plant Bio. 2014; l14:286.

49. Caceres JF, Kornblihtt AR. Alternative splicing: multiple control mechanisms and involvement in human disease. Trends Genet. 2002; 18(4):186–93. PMID: 11932019

50. Koh CM, Liu Y, Moehninsi, Du M, Ji L. Moehninsi Molecular characterization of KU70 and KU80 homologues and exploitation of a KU70-deficient mutant for improving gene deletion frequency in Rhodosporidium toruloides.BMC Microbiol. 2014; 27; 14:50. doi: 10.1186/1471-2180-14-50 PMID: 25188820

51. Rodriguez-Suarez C, Atienza SG, Piston F. Allelic variation, alternative splicing and expression analysis of Psy1 gene in Hordeum chilense Roem.et Schult. PloS One.2011; 6(5):e19885. doi: 10.1371/journal.pone.0019885 PMID: 21603624

52. Pillmann H, Hatje K, Odronitz F, Hammesfahr B, Kollmar M. Predicting mutually exclusive spliced exons based on exon length, splice site and reading frame conservation, and exon sequence homology. BMC bioinformatics.2011; 12:270 doi: 10.1186/1471-2105-12-270 PMID: 21718515

53. Zhu CX, Gu JY, Guo HJ, Zhao LS, Liu LX. Cloning and analysis of TaKu70 and TaKu80 inwheat. Journal of Nuclear Agricultural Sciences.2009; 23: 917–922

54. Hiyama A, Nohara C, Kinjo S, Taira W, Gima S, Tanahara A, et al. The biological impacts of the Fukushima nuclear accident on the pale grass blue butterfly. Sci Rep.2012; 2:570. doi: 10.1038/srep00570 PMID: 22880161

55. Gu JY, Wang Q, Cui M. Han B, Zhao LS, Liu LX, et al. Cloning and characterization of Ku70and Ku80 homologues involved in DNA repair process in wheat. Plant Genetic Resources.2014; 12:S99–S103.

56. Han B, Gu JY, Zhao LS, Guo HJ, Xie YD, Liu LX, et al. Molecular characterization of radiation Sensitivity of different wheat enotypes irradiated by γ rays. Journal of Plant Genetic Resources.2014; 15 (6): 1342–1347.

57. Garnier-Laplace J, Beaugelin-Seiller K, Hinton TG. Fukushima wildlife dose reconstruction signals ecological consequences. Environ Sci Technolo.2011; 45(12):5077–8.

58. Hiyama A, Nohara C, Taira W, Kinjo S, Iwata M, Otaki JM. The Fukushima nuclear accident and the pale grass blue butterfly: evaluating biological effects of long-term low-dose exposures. BMC Evol Biol. 2013; 13:168. doi: 10.1186/1471-2148-13-168 PMID: 23937355

59. Kryshev II, Kryshev AI, Sazykina TG. Dynamics of radiation exposure to marine biota in the area of the Fukushima NPP in March-May. J Enviro Radioact.2011: 114:157–61.

60. Cassidy CL, Lemon JA, Boreham DR. Impacts of low-dose gamma-radiation on genotoxic risk in aquatic ecosystems. Dose Response.2007; 5(4):323–32. doi: 10.2203/dose-response.07-026.Cassidy PMID: 18648569

61. Lehnert BE, Iyer R. Exposure to low-level chemicals and ionizing radiation: reactive oxygen species and cellular pathways. Hum Exp Toxicol.2002; 21(2):65–9. PMID: 12102498

62. Sun J, Chen Y, Li M, Ge Z. Role of antioxidant enzymes on ionizing radiation resistance. Free Radic Biol Med.1998; 24(4):586–93. PMID: 9559871

63. Won EJ, Lee JS. Gamma irradiation induces growth retardation, impaired egg production, and oxidative stress in the marine copepod Paracyclopina nana. Aquat Toxicol. 2014; 150:17–26. doi: 10.1016/j.aquatox.2014.02.010 PMID: 24632311

64. Depledge MH. The ecotoxicological significance of genotoxicity in marine invertebrates. Mutat Res.1998; 399(1):109–22. PMID: 9635493

65. Morgan MB, Vogelien DL, Snell TW. Assessing coral stress responses using molecular biomarkers of gene transcription. Environ Toxicol Chem 2001; 20(3):537–43. PMID: 11349854

66. Mahaney BL, Meek K, Lees-Miller SP. Repair of ionizing radiation-induced DNA double-strand breaks by non-homologous end-joining. Biochem J.2009; 417(3):639–50. doi: 10.1042/BJ20080413 PMID: 19133841
